# Identification of early quassinoid biosynthesis in the invasive tree of heaven (*Ailanthus altissima*) confirms evolutionary origin from protolimonoids

**DOI:** 10.3389/fpls.2022.958138

**Published:** 2022-08-23

**Authors:** Ling Chuang, Shenyu Liu, Dave Biedermann, Jakob Franke

**Affiliations:** ^1^Centre of Biomolecular Drug Research, Leibniz University Hannover, Hanover, Germany; ^2^Institute of Botany, Leibniz University Hannover, Hanover, Germany

**Keywords:** quassinoids, tree of heaven, *Ailanthus altissima*, triterpene, plant biochemistry, specialised metabolism, transcriptomics, protolimonoids

## Abstract

The tree of heaven, *Ailanthus altissima* (MILL.) SWINGLE, is a globally invasive plant known to secrete allelopathic metabolites called quassinoids. Quassinoids are highly modified triterpenoids. So far, nothing has been known about the biochemical basis of quassinoid biosynthesis. Here, based on transcriptome and metabolome data of *Ailanthus altissima*, we present the first three steps of quassinoid biosynthesis, which are catalysed by an oxidosqualene cyclase and two cytochrome P450 monooxygenases, resulting in the formation of the protolimonoid melianol. Strikingly, these steps are identical to the first steps of the biosynthesis of limonoids, structurally different triterpenoids from sister plant families within the same order Sapindales. Our results are therefore not only important to fully understand the biosynthesis of complex triterpenoids in plants, but also confirm the long-standing hypothesis that quassinoids and limonoids share an evolutionary origin. In addition, our transcriptome data for *Ailanthus altissima* will be beneficial to other researchers investigating the physiology and ecology of this invasive tree.

## Introduction

**FIGURE 1:**
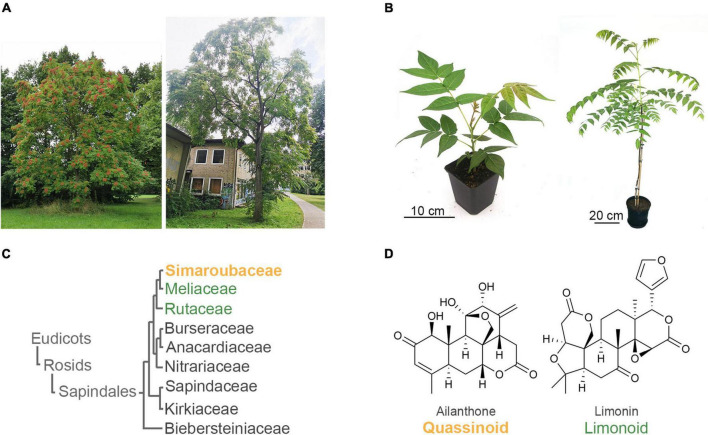
The invasive tree of heaven (*Ailanthus altissima*, Simaroubaceae) produces allelochemical triterpenoids called quassinoids with structural similarities to limonoids, which occur in the sister families Meliaceae and Rutaceae of the order Sapindales. **(A)** Tree of heaven in Hannover, Germany. **(B)** 8-week-old seedling and 3-year-old tree growing in the laboratory. **(C)** Relationship of families in the order Sapindales (The Angiosperm Phylogeny Group et al., 2016). **(D)** Structures of the representative quassinoid ailanthone and limonoid limonin.

Invasive species pose a great threat to biodiversity (Sladonja et al., 2015). *Ailanthus altissima* (MILL.) SWINGLE (Simaroubaceae), also known as the tree of heaven, is a particularly problematic invasive tree (Figures 1A,B). Originally from China and North Vietnam, *A. altissima* was introduced as an ornamental plant to Europe in the 1740s and is now invasive in all continents except Antarctica (Kowarik and Säumel, 2007). Indeed, the excessive and uncontrolled spread of *A. altissima* is recognised by many governments as problematic (Kowarik and Säumel, 2007); in the European Union, *A. altissima* was included in the list of Invasive Alien Species of Union concern in 2019. A key factor for the ecological success of *A. altissima* is the secretion of allelopathic compounds (Heisey, 1990a,b; Lawrence et al., 1991; Gómez-Aparicio and Canham, 2008), which were identified as a class of triterpenoids called quassinoids (Heisey, 1996; Martino, 2008; Li et al., 2021). Quassinoids are highly modified triterpenoids. Whereas triterpenoids, by definition, are derived from C_30_ precursors, typical quassinoids from *A. altissima* only possess C_20_ skeletons and are therefore often called decanortriterpenoids (Seigler, 1998; Curcino Vieira and Braz-Filho, 2006). Due to the complex chemistry and the challenges related to experimental work with trees compared to herbaceous plants, no experimental data exists for the biosynthesis of quassinoids so far. Another class of partially truncated triterpenoids, called limonoids, is known from the sister families Rutaceae and Meliaceae (order Sapindales) (Figures 1C,D). Due to certain structural similarities between limonoids and quassinoids, it was speculated that they are biosynthetically related (Polonsky, 1973; Das et al., 1987; Seigler, 1998; Curcino Vieira and Braz-Filho, 2006); however, this hypothesis has never been supported experimentally so far. Even though the limonoid biosynthetic pathway is also largely unknown, the first step of the pathway leads to structurally simpler C_30_ precursors called protolimonoids, which were recently elucidated (Hodgson et al., 2019; Lian et al., 2020; Pandreka et al., 2021). In the present study, we identified the first three committed steps of quassinoid biosynthesis. Based on *de novo* transcriptome sequencing of multiple tissues of the globally invasive tree *Ailanthus altissima* and comparison to metabolomic data, we selected suitable biosynthetic gene candidates and expressed them transiently in the common plant host *Nicotiana benthamiana* (Bally et al., 2018). Strikingly, our results show that quassinoid biosynthesis follows the same protolimonoid pathway known from limonoids, leading to the shared intermediate melianol. Our results, therefore pave the way for further elucidation of quassinoid and limonoid biosynthesis and will help to improve our understanding of the invasive properties of *A. altissima*.

## Materials and methods

### Plant material and plant growth conditions

Fresh seeds of *Ailanthus altissima* were collected from a wild adult tree in Hannover, Germany (52°21′43.9′′N 9°42′04.0′′E). The surrounding tissue of the winged seeds was removed, and the seeds were soaked in water at room temperature for 1 day. The next day, water was decanted and the seeds were covered with moist sand and cold-treated at 4°C for 2 weeks before planting. Cold-treated seeds were planted in starting soil 1 cm beneath the surface. The starting soil purchased from a local provider contained 70% organic material from moderately decomposed bog peat, white peat, and 30% clay with calcium carbonate and NPK fertiliser (180 mg/L N, 200 mg/L P_2_O_2_, 240 mg/L K_2_O, 130 mg/L S, and 150 mg/L Mg). The seeds were kept at 23°C in a growth chamber with 16 h of light and 55% humidity. The seeds started to germinate around 2 weeks later. Three days after germination, the seedlings were transferred to pots (7 cm × 7 cm width and length, 9 cm height) with potting soil from the same local provider. The potting soil contained 70% organic material from moderately decomposed bog peat, white peat and sod peat. The rest was composed of clay, calcium carbonate and NPK fertiliser (340 mg/L N, 380 mg/L P_2_O_2_, 450 mg/L K_2_O, 130 mg/L S, and 160 mg/L Mg). The potted seedlings were grown in the same growth chamber with the same settings as for germination until harvesting.

Young *A. altissima* trees (100–125 cm height) for purification of reference compounds were obtained from Baumschule-Aurea.de (Basedow, Germany).

Work with *Ailanthus altissima* in our research group is granted by permit DE-NI-2019-001 (NLWKN, Niedersächsischer Landesbetrieb für Wasserwirtschaft, Küsten- und Naturschutz) in addition to regulation (EU) No. 1143/2014.

*Nicotiana benthamiana* LAB strain (Bally et al., 2018) was grown from seeds in a greenhouse with 11–16 h of illumination per day and at a temperature between 21 and 23°C as described previously (Chuang and Franke, 2022).

### Chemical methods

NMR spectra were recorded using Bruker Ascend 400 or 600 MHz spectrometers operating at 400 and 600 MHz for ^1^H NMR and at 100 and 150 MHz for ^13^C NMR. CDCl_3_ and CD_3_OD were used as solvents. Chemical shifts were referenced relative to the residual solvent signals (CDCl_3_: δ_H_ = 7.26 ppm, δ_*C*_ = 77.16 ppm; CD_3_OD: 3.31 ppm, δ_C_ = 49.00 ppm) and expressed in δ values (ppm), with coupling constants reported in Hz. Analysis was conducted with TopSpin (Version 4.0.6, Bruker).

HRMS measurements were carried out on a Waters Alliance 2695 HPLC coupled to a Micromass LCT Premier mass spectrometer.

Analytical and semipreparative LCMS analyses were performed on an Agilent Infinity II 1260 system consisting of a G7167A autosampler, G7116A column thermostat, G7111B quaternary pump, G7110B make-up pump, G7115A diode array detector, G1364F fraction collector, and G6125B single quadrupole mass spectrometer equipped with an ESI source (positive mode, 4000 V, 12 L/min drying gas and 350°C gas temperature). Alternatively, for metabolomics work, a Waters system (Milford, MA, United States) was used consisting of a Waters 2998 photodiode array detector working from 210 to 400 nm and a Waters Quattro micro mass detector operating in ES+ and ES– modes between *m*/*z* 150 and 800. The columns and gradients used are described below.

### RNA-seq analysis and assembly

For RNA-Seq analysis, 14 samples in total were used from two biological replicates of young seedlings and two biological replicates of 3-year-old trees. See Supplementary Table 1 for a detailed sample list. The same samples were used for RNA extraction and metabolome analyses.

RNA from ca. 100 mg of fresh weight tissues was isolated using a published CTAB-LiCl method (Meisel et al., 2005). In short, plant tissue was ground in liquid nitrogen using a mortar and pestle. Total RNA was extracted using CTAB, cleaned up by phase extraction using chloroform-isoamyl alcohol (24:1), and precipitated using LiCl. The precipitated RNA was dissolved in SDS containing buffer, cleaned up again using chloroform-isoamyl alcohol (24:1) and precipitated using ethanol. The dried RNA pellet was dissolved in nuclease-free water and treated with DNase from TURBO-DNA free kit (Invitrogen AM1907). RNA quality was assessed using a Nanodrop, agarose gel electrophoresis, Invitrogen Qubit fluorometer, and Agilent Fragment Analyzer. Library preparation and sequencing were carried out by the GENEWIZ with Illumina NovaSeq 150 bp paired-end technology. Raw reads were deposited in SRA (Sequence Read Archive) under BioProject accession number PRJNA841173 (SRR19346858-SRR19346871).

Raw reads were assembled using Trinity 2.11.0 (Haas et al., 2013), including trimmomatic; all parameters were used with their default settings. Functional annotation was performed with Trinotate 3.1.1 by searching against the Swiss-Prot/UniProt and Pfam databases. Transcript quantification was achieved using Salmon 1.3.0 (Patro et al., 2017). Transcript counts were normalised using the TMM method (Robinson and Oshlack, 2010). The resulting annotation and quantification data were merged into a single annotated expression matrix for further analyses.

BUSCO analysis (Simão et al., 2015) was performed using the eudicots reference dataset.

### Purification and identification of ailanthone, chaparrinone, glaucarubinone, and amarolide

Purification of main quassinoids from *A. altissima* was performed based on previous reports (Tamura et al., 2006; Tung et al., 2017; Dang et al., 2019; Yang et al., 2021). In short, frozen plant tissues (roots, leaves, bark, phloem, and xylem of young trees) were ground in a blender, dried at 70°C for 48 h, and extracted overnight with 95/5 MeOH/H_2_O. The crude extracts were concentrated *in vacuo*, suspended in H_2_O, and sequentially extracted with petroleum ether, chloroform and *n*-butanol. The chloroform fractions were dissolved in acetonitrile and directly separated by semipreparative HPLC using a Phenomenex Kinetex C_18_ column (5 μm, 100 Å, 250 mm × 10 mm) at 40°C. Separation was achieved using a gradient (solvent A: H_2_O + 0.05% formic acid, solvent B: acetonitrile + 0.045% formic acid; linear gradient from 20 to 50% B over 10 min; flow rate 4.5 mL/min), resulting in the purification of ailanthone (*t*_R_ = 5.0 min), chaparrinone (5.5 min), glaucarubinone (*t*_R_ = 8.0 min), and amarolide (*t*_R_ = 7.2 min). All compounds were identified by comparison of NMR and MS data to literature (Yoshimura et al., 1984; Hirota et al., 1991; Grieco et al., 1993; Lin et al., 1995). NMR data are shown in Supplementary Figures 6–13 and Supplementary Tables 2–5.

### Metabolomic analyses

The same tissue samples used for RNA extraction were also used for metabolite extraction. For each sample, 100 mg ground tissue powder (fresh weight) was transferred into a 2 mL Eppendorf tube. Plant powders were extracted using 90% methanol in water with 1 mg/mL of limonin as the internal standard. The mixtures were shaken at 37°C and 900 rpm for 30 min. The solid debris was removed by centrifugation at 14,000 × *g* for 5 min. The supernatants were filtered through a 0.45 μm 13 mm diameter PTFE syringe filter (Chromafil MN729209) into a 1.5 mL glass vial for LCMS injection.

Quassinoids were detected in *Ailanthus altissima* extracts by LCMS. The samples were separated on a Phenomenex Kinetex column (2.6 μm, C18, 100 Å, 100 mm × 4.6 mm). 20 μL of the sample was injected and separated at a flow rate of 1 mL/min. The mobile phase consisted of H_2_O with 0.05% (v/v) formic acid as solvent A and acetonitrile with 0.045% (v/v) formic acid as solvent B. The column temperature was kept at 40°C. The gradient started at 10% B, increased to 50% B over 10 min, increased further to 90% until 11 min and kept for 3 min at 90% B before re-equilibration.

The peak area was integrated using MassLynx v4.1 and QuanLynx. The sum signal from the diode array detector (DAD) was used for quantifying the internal standard limonin (retention time 9.3 min). Extracted ion chromatograms (EIC) in positive mode with the following ions were used for quantifying each quassinoid: *m*/*z* 377 for ailanthone (retention time 3.9 min), *m*/*z* 495 for glaucarubinone (retention time 6.1 min), *m*/*z* 365 for amarolide (retention time 5.2 min), and *m*/*z* 379 for chaparrinone (retention time 4.3 min). The authenticity of all peaks and ions was confirmed using the purified reference compounds described above. Integrated areas (*A*) were exported to a csv file and plotted in R; the calibrated contents (*c*) were calculated based on sample dry weight (*m*) and the concentration of the internal standard limonin (720 μg/mL) as follows:


$$c_{Quassinoidcalibrated}=\frac{A_{QuassinoidEIC}\times 720\frac{\mu g}{mL}Limonin}{A_{LimoninDAD}\times m_{dryweight}}$$

### Bioinformatic analyses

For phylogenetic analysis, public transcriptome data of four Sapindales species and two species outside of Sapindales were included. The transcript sequences of orange (*Citrus sinensis*) and pomelo (*Citrus grandis*) from the Rutaceae family were downloaded from Citrus Pan-genome to Breeding Database (CPBD) v2.0 and v1.0, respectively (Xu et al., 2013; Wang et al., 2017). For the species in the Anacardiaceae family, mango (*Mangifera indica*) transcript sequences were kindly provided by Wang et al. (2020). Transcript sequences of yangbi maple (*Acer yangbiense*) in the Sapindaceae family were downloaded from GigaDB (Yang et al., 2019). *Arabidopsis thaliana* and *Nicotiana benthamiana* transcript sequences were downloaded from TAIR (Araport 11) and Solgenomics (genome sequence v1.0.1). To extract OSC sequences from total transcripts, HMMER (Finn et al., 2011) was used to identify amino acid sequences matching the squalene hopene cyclase pfam profile [SQHop_cyclase_N (PF13249)]. Extracted sequences with less than 600 amino acids were removed. To identify the citrus reference sequences from the public transcriptome data, CsOSC1 (XM_006468053.3, tirucalla-7,24-dien-3β-ol synthase), CsOSC2 (XM_025102137, β-amyrin synthase), and CsOSC3 (XM_015526998, lupeol synthase) (Hodgson et al., 2019) were downloaded from NCBI to blast against the public Citrus transcriptome. The three CsOSCs were identified as Cs2g08650.1, Cs8g20950.1, and Cs2g08550.1. Filtered amino acid sequences were aligned using Clustal Omega (Sievers and Higgins, 2018). The phylogenetic tree from the Clustal Omega alignment was constructed by the neighbour-joining method with the Jukes-Cantor genetic distance model without outgroup in Geneious Prime (2021.0.3). The phylogenetic tree was then exported as a Newick file for visualisation in R using the ggtree package (Yu et al., 2017).

For the identification of P450 candidates, all the P450 sequences were extracted from our *Ailanthus altissima* transcriptome assembly using the same method as for OSCs based on HMMER, but using the pfam profile p450 (PF00067). Pearson correlation coefficients were calculated and visualised in Python.

Heatmaps of OSC and P450 expression data were generated using expression values normalised by the TMM method (Robinson and Oshlack, 2010) and visualised in Python with the Seaborn package (Waskom et al., 2017).

### Transient expression in *Nicotiana benthamiana* and chromatographic analysis

Our method for transient expression is described by Chuang and Franke (2022). In short, selected gene candidates and booster genes were cloned into the plant expression vector pEAQ-HT (Sainsbury et al., 2009) using in-fusion cloning or into the improved vector pHREAC (Peyret et al., 2019) using Golden Gate cloning. Plasmids were sequenced by Sanger sequencing before transformation into *Agrobacterium tumefaciens* GV3101 by electroporation. Agrobacteria carrying candidate plasmids were mixed with strains carrying booster plasmids before infiltration into *Nicotiana benthamiana*. To identify booster genes, homologs of farnesyl pyrophosphate synthase (FPS) (AtFPS 1 and 2, AT5G47770, and AT4G17190), isopentenyl diphosphate delta isomerase (IPPI) (AtIPPI1 and 2, AT5G16440, and AT3G02780), squalene synthase (SQS) (AsSQS KY284575.1), and 3-hydroxy-3-methylglutaryl CoA reductase (HMGR) (AsHMGR KY284573.1) genes were identified by blast search against our *Ailanthus altissima* transcriptome. The contigs with the highest blast *E*-value and coverage were selected as putative *A. altissima* orthologs; these were also the contigs with the highest total expression. AaHMGR was amplified in truncated form (AatHMGR) to remove feedback sensitivity.

For the analytical scale screening, infiltration was done by needleless syringe into three leaves per plant. For the large-scale infiltration for compound isolation, infiltration was performed using a vacuum for the whole plant. Infiltrated plants were kept in a greenhouse for 7 days before harvesting. The gene sequences of AaHMGR (ON595691), AaIPPI (ON595692), AaFPS (ON595693), AaSQS (ON595694), AaOSC1 (ON595695), AaOSC2 (ON595696), AaOSC3 (ON595697), AaCYP71CD4 (ON595698), and AaCYP71BQ17 (ON595699) were deposited in GenBank. The primers used for cloning are listed in Supplementary Table 9.

For the analytical scale screening, five-leaf disks were harvested using cork borer no. 5 (diameter 10 mm) from the co-expressing *N. benthamiana* leaves, lyophilised overnight and ground using a ball mill.

For the OSC product detection, the method was described previously (Chuang and Franke, 2022). In short, the ground leaf powder was treated with 10% (w/v) KOH in 90% ethanol (v/v) at 70°C for 1 h to saponify triterpene esters, and then extracted with *n*-hexane with 10 ng/μL internal standard (5α-cholestane). Vacuum-dried extract was then dissolved and silylated in 1:1 pyridine:BSTFA/TMCS (1% in ethyl acetate) at 70°C for 1 h before GCMS injection. Samples were injected into an Agilent 6890N Network GC system connected with mass selective detector 5973. Samples were separated on a low bleed 5% phenyl dimethylpolysiloxane column (Optima 5 HT, 30 m length, 0.25 mm inner diameter and 0.25 μm film thickness). The carrier gas was helium at a flow rate of 1.5 mL/min. The temperature gradient started at 100°C, increased by 30°C/min to 275°C, then increased by 3°C/min to 300°C and held at 300°C for 15.83 min. The mass detector used a scan range from *m*/*z* 43 to 650.

For the detection of oxidised triterpenes, ground leaf powders were extracted with 90% methanol in H_2_O and extracts were analysed by LCMS. The samples were separated on a Phenomenex Kinetex column (2.6 μm, C8, 100 Å, 150 mm × 4.6 mm) with a mobile phase consisting of H_2_O + 5 mM NH_4_OAc (solvent A) and MeOH + 5 mM NH_4_OAc (solvent B). The gradient started at 90% B, increased to 100% B over 10 min, and was maintained for a minute before re-equilibration. The flow rate was 1 mL/min. The column temperature was set to 50°C. MSD was operated in positive mode with a mass detection range from 200 to 800 *m*/*z*.

### Compound purification and characterisation

For the production and purification of tirucalla-7,24-dien-3β-ol, 17 *N. benthamiana* plants were vacuum-infiltrated with *A. tumefaciens* strains harbouring booster genes (*AatHMGR*, *AaIPPI*, *AaFPS*, and *AaSQS*) and *AaOSC2* and incubated for 7 days in a greenhouse. Afterwards, leaves were harvested, flash-frozen, ground in a blender and dried at 70°C for 24 h. The crude plant material was extracted with 250 mL ethyl acetate overnight, filtered and concentrated *in vacuo*. This crude extract was dissolved in THF and directly purified by mass-guided semipreparative HPLC using a Phenomenex Luna C8(2) column (5 μm, 100 Å, 250 mm × 10 mm) at 50°C. Separation was achieved using a gradient (solvent A: H_2_O + 5 mM NH_4_OAc, solvent B: MeOH + 5 mM NH_4_OAc; linear gradient from 90 to 100% B over 15 min; flow rate 5 mL/min). The peak corresponding to tirucalla-7,24-dien-3β-ol eluting at 12.6 min was collected based on ESI-MS signal *m*/*z* 427 ([M+H]^+^). The corresponding fractions from multiple runs were pooled, concentrated *in vacuo* and analysed by NMR, confirming the isolation of 6 mg tirucalla-7,24-dien-3β-ol as a colourless powder.

Tirucalla-7,24-dien-3β-ol: Colourless powder; ^13^C and ^1^H NMR data are given in Supplementary Table 6 and Supplementary Figures 14, 15.

For the production and purification of melianol, 120 plants were vacuum-infiltrated with *A. tumefaciens* strains harbouring the genes *AstHMGR* from oat (*Avena sativa*) (Reed et al., 2017), *AaOSC2*, *AaCYP71CD4*, and *AaCYP71BQ17*, and incubated for 7 days in a greenhouse. Afterwards, leaves were harvested, flash-frozen, ground in a blender, and dried at 70°C for 24 h. The crude plant material was extracted with 450 mL ethyl acetate overnight, filtered and concentrated *in vacuo*. This crude extract of *N. benthamiana* was suspended in 150 mL water and sequentially extracted with petroleum ether (PE) 3 × 200 mL, chloroform 2 × 200 mL, ethyl acetate (EA) 2 × 100 mL and *n*-butanol 2 × 100 mL. Melianol was detected using LCMS in the PE and the chloroform fractions. Hence, both fractions were combined (4.9 g) and purified by multiple rounds of flash chromatography (Biotage Isolera One) (Supplementary Table 7). For final purification, column chromatography on silica (Silica 60 M, 0.04–0.063 mm, Macherey-Nagel) was carried out with PE:EA = 1:1, yielding 42 mg melianol as a colourless powder.

Melianol: Colourless powder; HR-ESI-MS: *m*/*z* 495.3448 [M+Na]^+^ (calcd. for C_30_H_48_O_4_Na^+^, 495.3445); ^13^C and ^1^H NMR data are given in Supplementary Table 8 and Supplementary Figures 16, 17.

## Results

### Metabolome and transcriptome profiling of *Ailanthus altissima* for identifying biosynthetic genes

**FIGURE 2:**
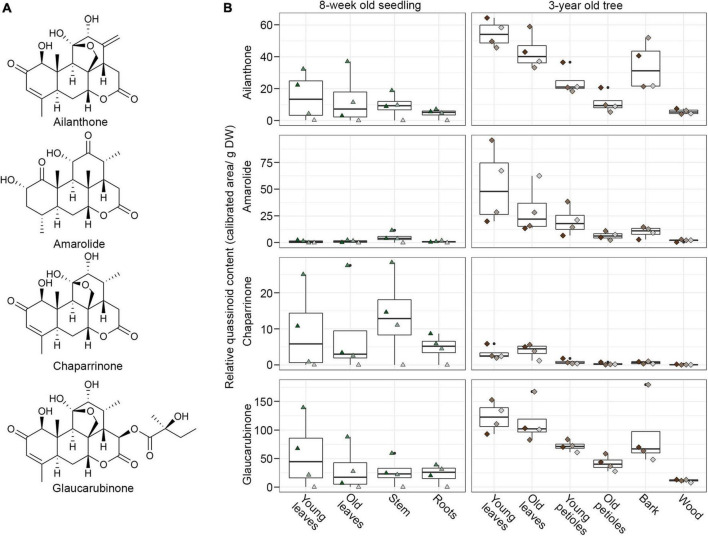
Distribution of four main quassinoids in different tissues of *Ailanthus altissima* seedlings and trees. **(A)** Structures of main quassinoids. **(B)** Levels of the four main quassinoids as determined by LCMS. The data is represented samples were normalised by dry weight to account for differences in tissue water content. The data distribution is represented as a boxplot; the centre line of the box indicates the median value, whereas the lower and upper borders of the box correspond to the first and third quartile, respectively; the whiskers indicate the lowest and highest data points, excluding outliers.

A key strategy to identify biosynthetic genes is to correlate metabolic profiles with gene expression across different producing and non-producing tissues (Caputi et al., 2018; Dang et al., 2018; Nett et al., 2021). Despite the relevance of *A. altissima* as a globally invasive species, no comprehensive transcriptomic data is publicly available for this plant. Likewise, very little is known about the distribution of main metabolites, such as ailanthone across different tissues. To find quassinoid biosynthetic genes, we therefore selected different tissues for metabolite and transcriptome analyses to enable downstream co-expression analyses. In total, we selected samples from 10 different tissues of either freshly grown seedlings or 3-year-old trees to represent a wide range of biological conditions (Supplementary Table 1). The tissues covered were young leaves, old leaves, petioles, roots, stem, bark, and wood. For each sample, we performed metabolite extraction followed by LCMS analysis and RNA isolation for RNA-Seq in parallel. To obtain reference compounds for metabolite quantification, we purified the major quassinoids from the plant material and confirmed their identity by NMR spectroscopy, resulting in ailanthone, chaparrinone, glaucarubinone, and amarolide (Figure 2). The levels of these four metabolites were quantified in four biological replicates, each for young seedlings and trees, including the 14 samples that were eventually used for RNA-sequencing (Figure 2). To account for differences in tissue water content, all samples were normalised by dry weight.

For RNA-Seq, RNA in sufficient quality was successfully isolated from 14 samples, representing 8 of the 10 tissues used for metabolite quantification; surprisingly, all isolated RNA from young leaves or old leaves from 3-year-old trees did not pass the initial quality control and was therefore dismissed. For sequencing of the 14 samples of sufficient quality, RNA was converted to cDNA and sequenced by short-read Illumina sequencing using 150 bp paired end technology. In total, 242,285,498 raw reads were generated, corresponding to 36.3 Gb raw data. The raw reads exhibited a mean quality score of 36.3, suitable for further assembly. After the removal of the adapter and low-quality sequences, the raw reads were *de novo* assembled using Trinity (Haas et al., 2013). In total, this resulted in 783,937 unfiltered transcripts (Table 1). To assess the completeness of our assembly, we performed BUSCO analysis (Simão et al., 2015). In comparison to the eudicots reference dataset, 93.9% of expected complete single-copy orthologs were detected, and only 3.2 and 2.9% were fragmented or missing, respectively (Table 1). Even though the proportion of duplicated sequences was very high at 74.5%, which was not surprising considering the inflated number of unfiltered transcripts, we decided to continue with the full unfiltered assembly to avoid erroneous clustering of physiologically distinct paralogous genes and isoforms. Transcripts were annotated by comparison to Swiss-Prot/UniProt and Pfam databases, and quantified using Salmon (Patro et al., 2017), followed by TMM normalisation (Robinson and Oshlack, 2010). This resulted in a comprehensive expression matrix suitable for discovering biosynthetic genes by co-expression analysis.

**TABLE 1:** RNA-Seq sequencing statistics for *de novo* transcriptome assembly of 14 different *A. altissima* samples.

Type	Value
Total input reads	242,285,498
Total length of raw data (bp)	36,342,824,700
Total assembled transcripts	783,937
Total assembled transcript bases (bp)	495,402,342
GC% of assembly	43.10
BUSCO score	C: 93.9% (S: 19.4%, D: 74.5%), F: 3.2%, M:2.9%, n:2326

### The *Ailanthus altissima* oxidosqualene cyclase AaOSC2 is a tirucalla-7,24-dien-3β-ol synthase

**FIGURE 3:**
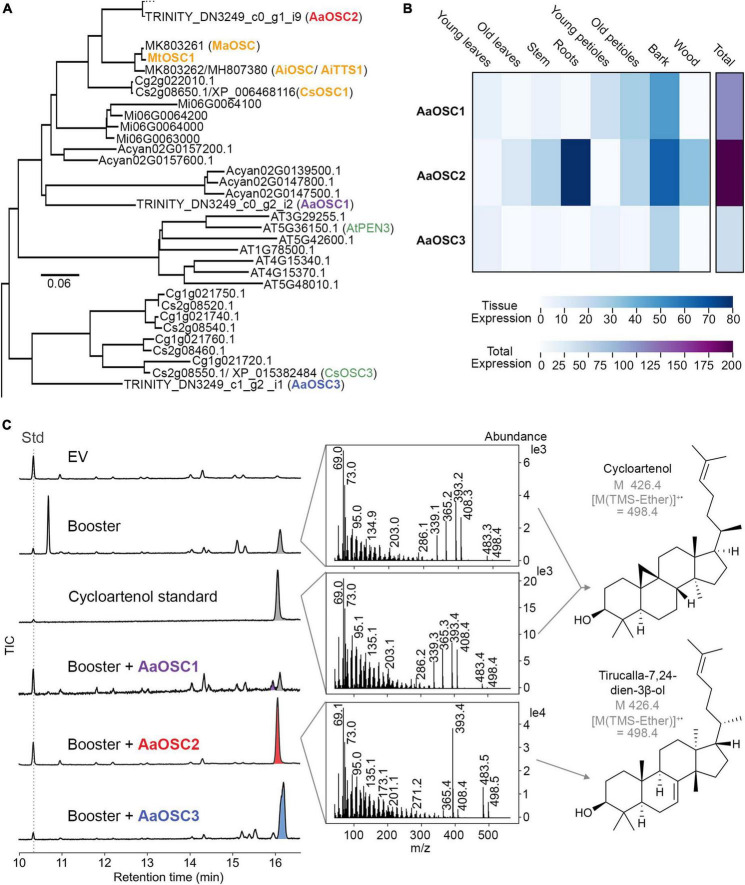
The oxidosqualene cyclase (OSC) AaOSC2 from *Ailanthus altissima* is a tirucalla-7,24-dien-3β-ol synthase. **(A)** Partial phylogenetic tree of OSCs; the full tree is shown in Supplementary Figure 1. OSC amino acid sequences were extracted from five Sapindales species, including the tree of heaven (*Ailanthus altissima*), orange (*Citrus sinensis*), pomelo (*Citrus grandis*), mango (*Mangifera indica*), and yangbi maple (*Acer yangbiense*), and two species outside of Sapindales, including *Arabidopsis thaliana* and *Nicotiana benthamiana*, were aligned using Clustal Omega, and a phylogenetic tree reconstructed using the neighbour-joining method. The scale bar indicates the phylogenetic distance. **(B)** Expression levels of each contig (trimmed mean of *M*-values (TMM)) are visualised as a heat map. Young leaf, old leaf, stem and root tissues are from 8-week-old seedlings. Young petioles, old petioles, bark and wood are from 3-year-old trees. **(C)** GCMS total ion chromatograms of each candidate *AaOSC* co-expressed with booster genes in *Nicotiana benthamiana*. EV is empty vector control. Booster genes include *AatHMGR*, *AaFPS*, *AaIPPI*, and *AaSQS*. 5α-cholestane was used as an internal standard (Std). Mass spectra of co-eluting peaks of cycloartenol and tirucalla-7,24-dien-3β-ol at 16.0 min (grey and red, respectively) are shown. Chromatograms and mass spectra for AaOSC1 and AaOSC3 products (purple and blue, respectively) are shown in Supplementary Figures 3, 4.

With the transcriptome data in our hands, we set out to identify the first enzyme of the quassinoid pathway. Based on the structural similarities of quassinoids to limonoids, we anticipated that the first committed step should be catalysed by an oxidosqualene cyclase (OSC) related to tirucalla-7,24-dien-3β-ol synthase from limonoid biosynthesis (Hodgson et al., 2019; Lian et al., 2020; Pandreka et al., 2021). We therefore selected all 22 transcripts encoding oxidosqualene cyclases (sequences provided as Supplementary material) from our transcriptome dataset and generated a phylogenetic tree to enable functional predictions (Figure 3A and Supplementary Figure 1). Transcripts with very low total expression, which likely represent assembly artefacts, were excluded from further investigation. In total, we identified three full-length (ca. 2.3 kb) OSC-encoding transcripts, named *AaOSC1*, *AaOSC2*, and *AaOSC3*, that are phylogenetically close to the characterised tirucalla-7,24-dien-3β-ol synthases from orange (*Citrus sinensis*) (CsOSC1), *Melia azedarach* (MaOSC1) (Hodgson et al., 2019), *Azadirachta indica* (AiOSC1 / AiTTS1) (Hodgson et al., 2019; Pandreka et al., 2021), and *Melia toosendan* (MtOSC1) (Lian et al., 2020). Notably, these are only distantly related to *Arabidopsis thaliana* PEN3 (AtPEN3), which was previously shown to produce tirucalla-7,24-dien-3β-ol as well (Morlacchi et al., 2009). Of our three candidates AaOSC1-3, AaOSC2 showed the highest amino acid identity to tirucalla-7,24-dien-3β-ol synthases from related species, namely 85% (CsOSC1), 87% (AiOSC1/AiTTS1), 88% (MaOSC1), and 87% (MtOSC1); in comparison, for AaOSC1 and AaOSC3, respectively, the maximum amino acid identities to any of these tirucalla-7,24-dien-3β-ol synthases were 75 and 67%. *AaOSC1* and *AaOSC3* were most strongly expressed in bark samples, whereas *AaOSC2* was also strongly expressed in the roots of seedlings (Figure 3B). These expression patterns were also independently confirmed by semi-quantitative RT-PCR (Supplementary Figure 2).

To test the enzymatic function of the encoded enzymes, we amplified *AaOSC1* from the cDNA of seedling leaves and *AaOSC2* and *AaOSC3* from the cDNA of tree bark. The amplified genes were cloned into the expression vectors pEAQ-HT (*AaOSC1* and *AaOSC3*) (Sainsbury et al., 2009) and pHREAC (*AaOSC2*) (Peyret et al., 2019) for transient expression in *N. benthamiana*. Gene candidates were first expressed alone in *N. benthamiana*, but only trace amounts of products could be observed by GCMS (data not shown). To increase the yields, booster genes to increase the levels of the substrate 2,3-oxidosqualene (Guo et al., 2020) were cloned from *Ailanthus altissima* and co-expressed with candidate genes. The booster genes encoded farnesyl pyrophosphate synthase (FPS), isopentenyl diphosphate delta isomerase (IPPI), and squalene synthase (SQS), and a feedback-insensitive truncated 3-hydroxy-3-methylglutaryl CoA reductase (tHMGR). Co-expression of feedback-insensitive HMGR was previously shown to increase the production of triterpenes in *N. benthamiana* (Reed et al., 2017). Expression of *AaOSC1* resulted in the formation of four new metabolites in low quantities (Supplementary Figure 3), which could not be identified by comparison to common triterpene standards, and also could not be purified successfully due to the low titres. The mass spectra corresponding to these four peaks (Supplementary Figure 3) suggested no similarity to tirucalla-7,24-dien-3b-ol, however (Hodgson et al., 2019). AaOSC3 expression led to the formation of a single major new product. Comparing its retention time and mass spectrum with an authentic standard, this product was identified as lupeol (Supplementary Figure 4). Hence, AaOSC3 was identified as a lupeol synthase. For AaOSC2, we did not observe any obvious activity at the first glance. Closer inspection of the mass spectra of the peaks at 16.0 min revealed a clear difference between *AaOSC2*-expressing leaves and controls not expressing any exogenous *OSC* gene (Figure 3C). In control samples, the peak at 16.0 min could be unambiguously identified as cycloartenol based on a comparison of the retention time and mass spectrum with an authentic standard. However, in *AaOSC2*-expressing samples, a compound with an indistinguishable retention time, but a different mass spectrum was observed. Indeed, careful re-analysis of extracted ion chromatograms (EICs) showed that *AaOSC2* expression led to a decrease of *m*/*z* 339 with a concomitant increase of *m*/*z* 498 at 16.0 min compared to controls and other *OSC* expression experiments (Supplementary Figure 5), confirming that the main product of AaOSC2 eluting at 16.0 min is not cycloartenol, but a different triterpene.

To confirm the structure of the AaOSC2 product, we scaled up the expression to 17 *N. benthamiana* plants and isolated the resulting compound by chromatography. NMR analysis confirmed that the AaOSC2 product is tirucalla-7,24-dien-3β-ol, the same triterpene scaffold that has recently been identified as the key starting point in the biosynthesis of limonoids (Hodgson et al., 2019; Lian et al., 2020; Pandreka et al., 2021). Hence, we rename AaOSC2 as *A. altissima* tirucalla-7,24-dien-3β-ol synthase (AaTS). This finding provides for the first time molecular evidence for the hypothesis that quassinoids and limonoids are indeed related classes of natural products.

### Two cytochrome P450 monooxygenases AaCYP71CD4 and AaCYP71BQ17 convert tirucalla-7,24-dien-3β-ol into the protolimonoid melianol

**FIGURE 4:**
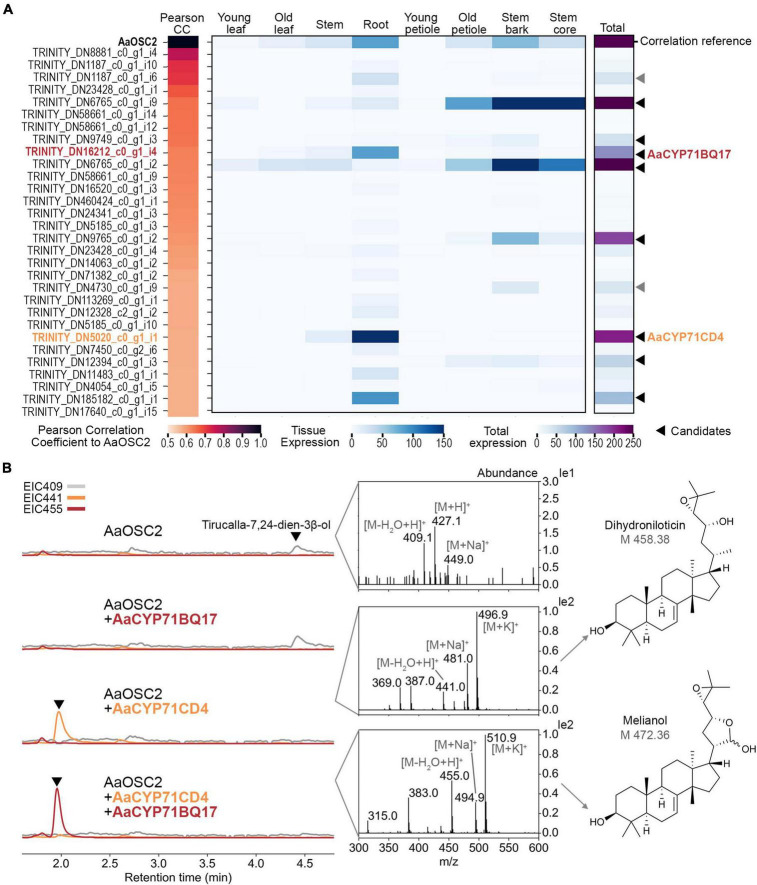
Discovery of the two cytochrome P450 enzymes AaCYP71CD4 and AaCYP71BQ17 which convert tirucalla-7,24-dien-3β-ol into the protolimonoid melianol during quassinoid biosynthesis. **(A)** Top 30 cytochrome P450-encoding contigs co-expressed with the tirucalla-7,24-dien-3β-ol synthase gene *AaTS* ranked by Pearson’s correlation coefficients (CC). Pearson’s CC values and expression data [trimmed mean of *M*-values (TMM)] are shown as a heatmap. Young leaf, old leaf, stem and root tissues were from 8-week-old seedlings; young petioles, old petioles, bark and wood were from 3-year-old trees. The 10 candidates with the highest expression (indicated by arrows) were selected for functional validation by co-expression. Of these, two candidates could not be cloned successfully (grey arrows). **(B)** Extracted ion chromatograms of co-expression experiments in *Nicotiana benthamiana* analysed by LCMS. All candidates were co-expressed with booster genes. Mass spectra of the indicated peaks (arrows) are shown. The product from co-expression of *AaOSC2*, *AaCYP71CD4*, and *AaCYP71BQ17* was confirmed as melianol by NMR.

We next sought to identify further genes of the quassinoid pathway. Considering that quassinoids are highly oxidised and cytochrome P450 monooxygenases (P450s) are key drivers of triterpene modifications (Thimmappa et al., 2014; Ghosh, 2017; Hu et al., 2020), we screened our transcriptome data for genes encoding P450s co-expressed with *AaTS*. For that, Pearson correlation coefficients were calculated for all transcripts encoding cytochrome P450 domains (Figure 4A). Strikingly, a closer analysis of the candidates ranked by correlation coefficients revealed that two co-expressed candidates (#9 and #24) were highly similar to MaCYP71CD2 and MaCYP71BQ5 (86 and 89% amino acid identity, respectively) from limonoid biosynthesis, which convert tirucalla-7,24-dien-3β-ol into melianol (Hodgson et al., 2019).

To elucidate further steps of quassinoid biosynthesis, we selected a total of 10 P450 candidates with the highest overall expression levels from this co-expression dataset for functional evaluation. As mentioned earlier, gene candidates were amplified from cDNA derived from either seedling roots or tree bark, cloned into the transient expression vectors pEAQ-HT or pHREAC and co-expressed together with booster genes and *AaTS*. Eight of these candidates were cloned successfully. Leaf samples were analysed by LCMS instead of GCMS to account for the expected increase in polarity. Gratifyingly, co-expression of *AaCYP71CD4*, booster genes and *AaTS* resulted in a new peak with a mass shift of 32, suggesting the incorporation of 2 oxygen atoms (Figure 4B). Mass data suggested that this peak corresponds to dihydroniloticin, the product of the homologous enzymes CYP71CD1 and CYP71CD2 in limonoid biosynthesis (Hodgson et al., 2019). When we co-expressed *AaCYP71CD4*, *AaCYP71BQ17*, *AaTS* and booster genes, this new peak was converted into yet another new compound with a putative molecular weight of 472 compared to 426 for tirucalla-7,24-dien-3β-ol. In the absence of *AaCYP71CD4*, no new peak was observed when *AaCYP71BQ17*, *AaTS*, and booster genes were co-expressed, suggesting a strict order of transformations. To identify the structure of the product of AaCYP71BQ17, we purified it from large-scale *N. benthamiana* infiltration experiments (120 plants) and fully characterised it spectroscopically. NMR analysis clearly showed that the product of the two P450s was melianol, obtained as an inseparable mixture of lactol epimers as reported earlier (Nakanishi et al., 1986; Hodgson et al., 2019). Melianol is a protolimonoid that was recently identified as an intermediate in limonoid biosynthesis (Hodgson et al., 2019). All other 6 P450 candidates which were additionally tested did not show any activity on either tirucalla-7,24-dien-3β-ol, dihydroniloticin, or melianol. In conclusion, our results indicate that melianol is a shared intermediate in quassinoid and limonoid biosynthesis, and that quassinoids like limonoids, have evolved from a protolimonoid precursor.

## Discussion

**FIGURE 5:**
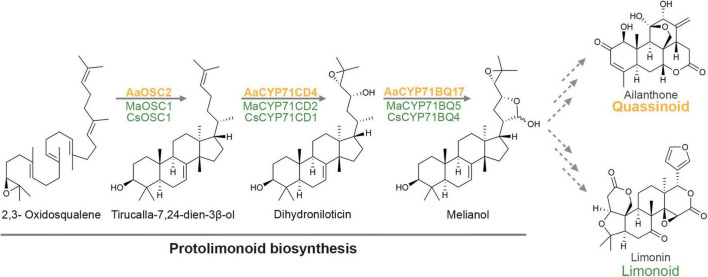
Current model for quassinoid biosynthesis, highlighting the shared protolimonoid origin of quassinoids and limonoids up to melianol.

In the present study, we identified the first three committed steps of the biosynthetic pathway of allelopathic quassinoids in the invasive tree *Ailanthus altissima*. The combined enzymatic action of the oxidosqualene cyclase AaTS and the P450s AaCYP71CD4 and AaCYP71BQ17 leads to the production of the protolimonoid melianol, which is known from limonoid biosynthesis (Hodgson et al., 2019). Even though it has previously been hypothesised that limonoid and quassinoid biosynthetic pathways are related (Polonsky, 1973; Das et al., 1987), we provide the first molecular evidence that both pathways indeed follow the same course at least up to the protolimonoid melianol, based on evolutionary conserved genes (Figure 5). Due to the lack of facile gene silencing systems in limonoid or quassinoid producing plants, the *in planta* function of these genes remains yet to be confirmed. Also, labelling experiments will be important to provide additional evidence for the suggested key role of melianol in both pathways. From a structural point of view, it is surprising that the side chain oxidations *en route* to melianol are still part of the quassinoid biosynthetic pathway, as most quassinoids completely lack this side chain. Besides shedding light on the evolutionary history of both pathways, these conserved genes might therefore suggest that the biochemical transformations for cleavage of the side chain are facilitated by these functionalisations.

In our work, we also provide the first comprehensive transcriptome dataset of the invasive tree *Ailanthus altissima*. Although a leaf transcriptome for *A. altissima* is available from the 1,000 plant transcriptome initiative (sample code QICX) (Carpenter et al., 2019; Leebens-Mack et al., 2019), we could only find a full-length copy of *AaOSC1* in their data, but not *AaOSC2*, *AaOSC3*, *AaCYP71CD4*, or *AaCYP71BQ17*. This underlines that our transcriptome data represent a substantially improved resource for identifying genes from *A. altissima* and elucidating its metabolic pathways. Our data show that all three early quassinoid biosynthetic pathway genes are co-expressed. It is well known that co-expression analyses are powerful tools for elucidating biosynthetic pathways in plants (Caputi et al., 2018; Dang et al., 2018; Nett et al., 2021), and this also holds true for the early steps of quassinoid biosynthesis. We therefore anticipate that our data will also be useful for the identification of further pathway steps downstream of melianol. Strikingly, however, the distribution of main quassinoids throughout the plant that we observed by metabolomic analyses was not consistent with this gene expression pattern. This strongly suggests that transport processes to different tissues must play an important role at certain stages of the quassinoid pathway. As quassinoids are important allelochemicals in *A. altissima*, these transport processes are crucial for the ecological function of quassinoids and will therefore require special attention in the future. For limonoids, similar transport processes have been reported (Hasegawa et al., 1986). It is possible that not only the biosynthetic machinery for limonoids and quassinoids is related, but also the corresponding transporters.

In summary, we identified the first three steps of the biosynthesis of allelopathic quassinoids from the tree of heaven (*Ailanthus altissima*), a globally invasive species. Our data show that quassinoids—like limonoids—are derived from the protolimonoid melianol based on homologous enzymes, even though both classes of triterpenoids feature largely different skeletal modifications. The metabolomic, transcriptomic and biochemical data provided herein will serve as a fruitful basis for further elucidation of the biosynthesis and transport machinery of quassinoids as well as limonoids in the future.

## Data availability statement

The datasets presented in this study can be found in online repositories. The names of the repository/repositories and accession number(s) can be found below: https://www.ncbi.nlm.nih.gov/genbank/, ON595691-ON595699, https://www.ncbi.nlm.nih.gov/, PRJNA841173 (SRR19346858-SRR19346871).

## Author contributions

LC and JF conceived and designed the study and carried out RNA-Seq analysis. LC performed the metabolomics analyses, candidate selection, and all expression experiments. SL and DB purified compounds and carried out structure elucidation. JF, LC, and SL wrote the manuscript. JF supervised the research and acquired funding. All authors read and approved the final manuscript.
