# Supplementary material for: Identification of early quassinoid biosynthesis in the invasive tree of heaven (Ailanthus altissima) confirms evolutionary origin from protolimonoids
Source: Front Plant Sci. 2022 Aug 23;13:958138. doi: 10.3389/fpls.2022.958138 (PMC9445810; doi:10.3389/fpls.2022.958138)
Supplement: Supplementary file 1 [file Data_Sheet_1.pdf]

Supplementary Material

Supplementary Table 1. List of samples used for metabolome and transcriptome analyses

Biological replicate	Tissue name	RNA Sequencing	
		RQN [a]	# reads
Seedling 1 and seedling 3	Young leaf	NA [b]	
	Old leaf		
	Stem		
	Root		
Seedling 2	Young leaf	5.2	23,125,865
	Old leaf	5.5	23,119,884
	Stem	7.5	24,857,387
	Root	7.7	NA [b]
Seedling 4	Young leaf	4.9	22,865,719
	Old leaf	5.9	23,423,192
	Stem	9.1	30,434,405
	Root	6.6	20,764,331
Tree 1	Young leaf	NA	NA [b]
	Young petiole	6.2	24,852,611
	Old leaf	3.4	NA [b]
	Old petiole	5.0	NA [b]
	Bark	6.2	21,308,242
	Wood	7.5	21,887,768
Tree 2 and tree 4	Young leaf	NA [b]	
	Young petiole		
	Old leaf		
	Old petiole		
	Bark		
	Wood		
Tree 3	Young leaf	NA	NA [b]
	Young petiole	7.6	23,045,108
	Old leaf	4.9	NA [b]
	Old petiole	6.7	23,193,419
	Bark	6.1	24,207,108
	Wood	5.5	29,238,073

[a] RNA Quality Number

[b] No good-quality RNA could be obtained, or sequencing was not successful.

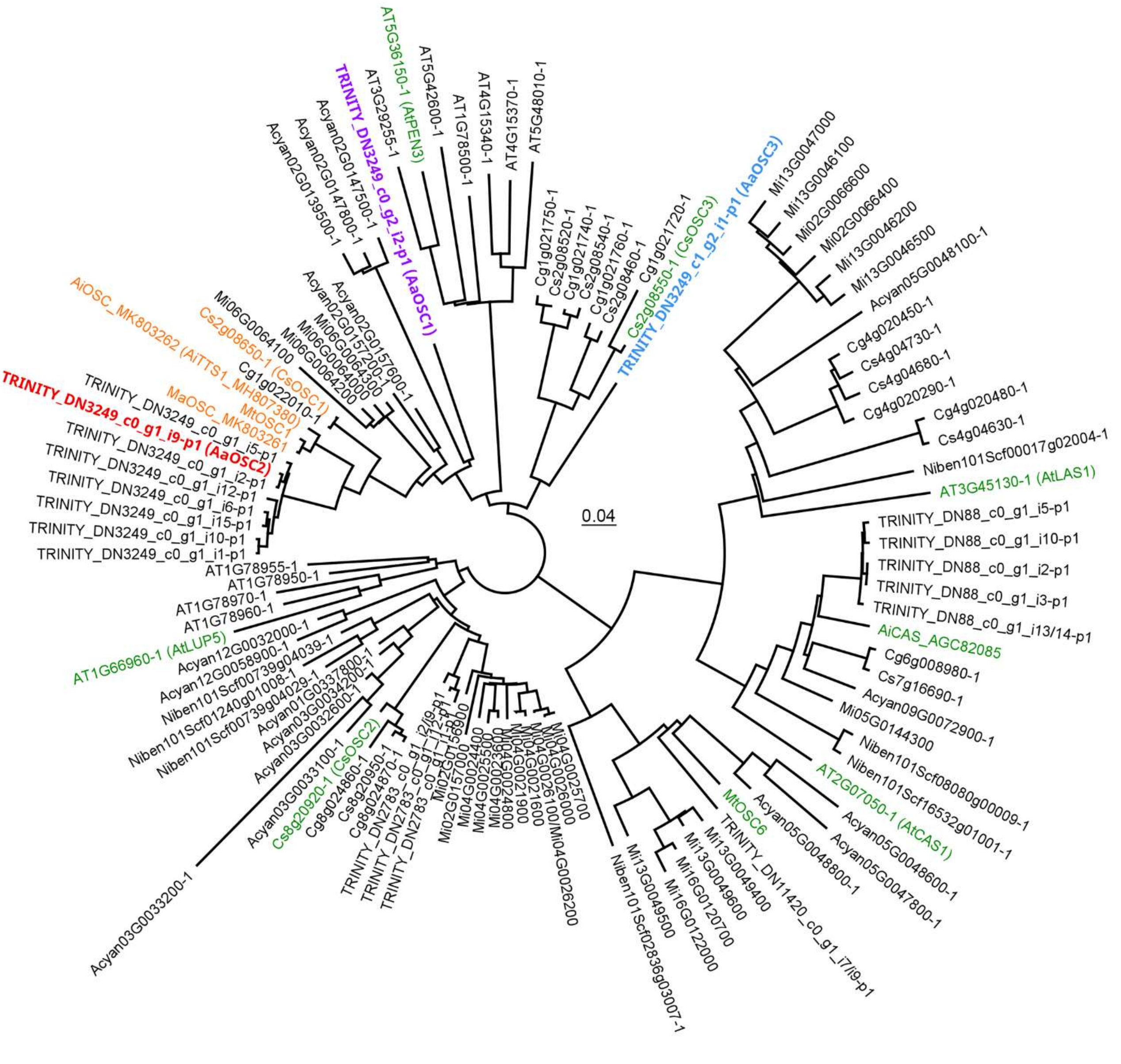

Supplementary Figure 1. Phylogenetic tree of oxidosqualene cyclase (OSC) amino acid sequences from five species in Sapindales order and two species outside of Sapindales order. Species from Sapindales: Aa: *Ailanthus altissima* (Simaroubaceae); Acyan: *Acer yangbiense* (Sapindaceae); Cg: *Citrus grandis* (Rutaceae); Cs: *Citrus sinensis* (Rutaceae); Mi: *Mangifera indica* (Anacardiaceae). Species outside of Sapindales: At: *Arabidopsis thaliana*; Niben: *Nicotiana benthamiana*. For further details see also Figure 3A.

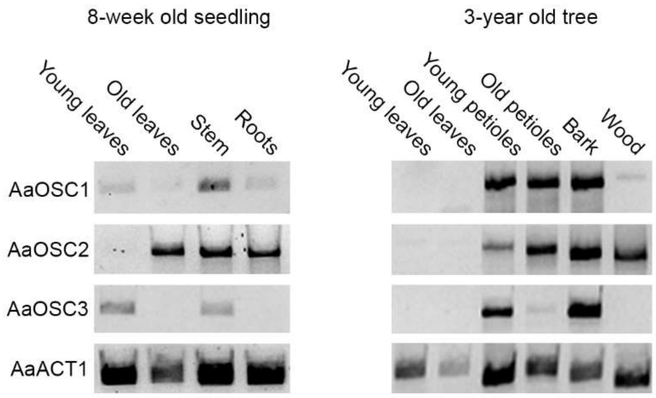

Supplementary Figure 2. Expression profiles of *AaOSC1/2/3* genes based on semi-quantitative RT-PCR analysis, using cDNA from different tissues of a tree of heaven seedling and a 3-year old tree. Actin 1 was used as a control. Similar results were obtained using four biological replicates for each tissue from 8-week-old seedlings and two biological replicates for each tissue from 3-year-old trees.

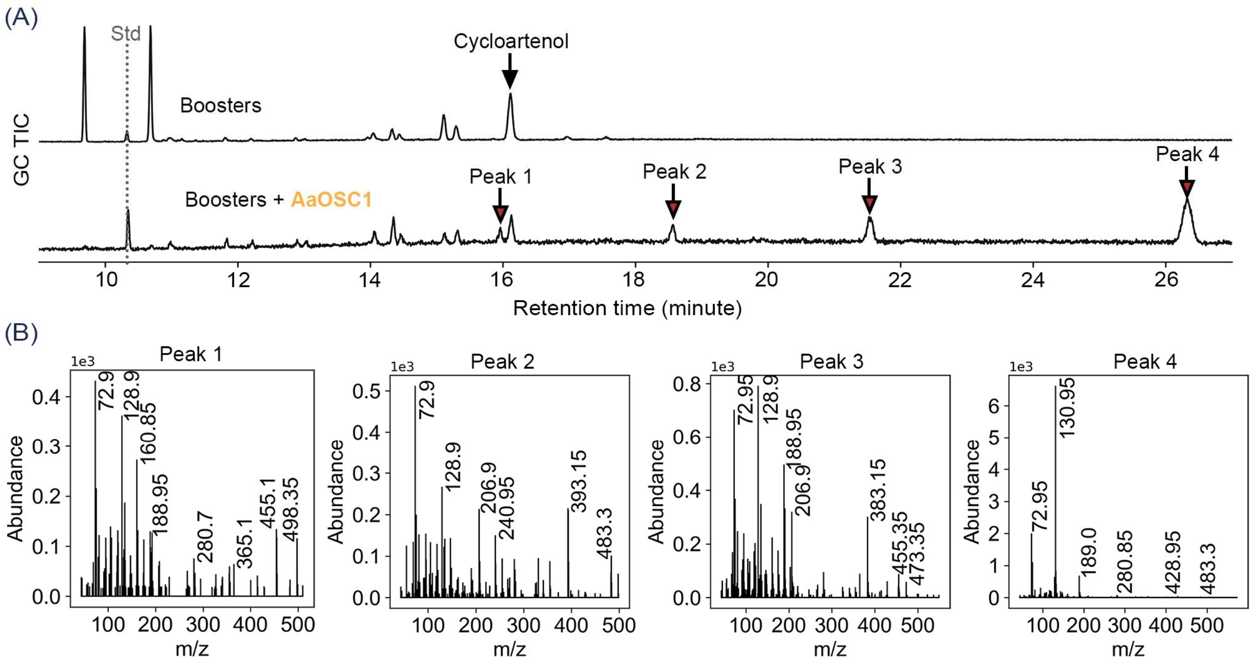

Supplementary Figure 3. GCMS analysis (total ion chromatograms) of *AaOSC1* expression in *N. benthamiana* shows four new, unknown products compared to a control. 5α-cholestane was used as an internal standard (Std) for extraction. Mass spectra of the four products produced by AaOSC1 are shown.

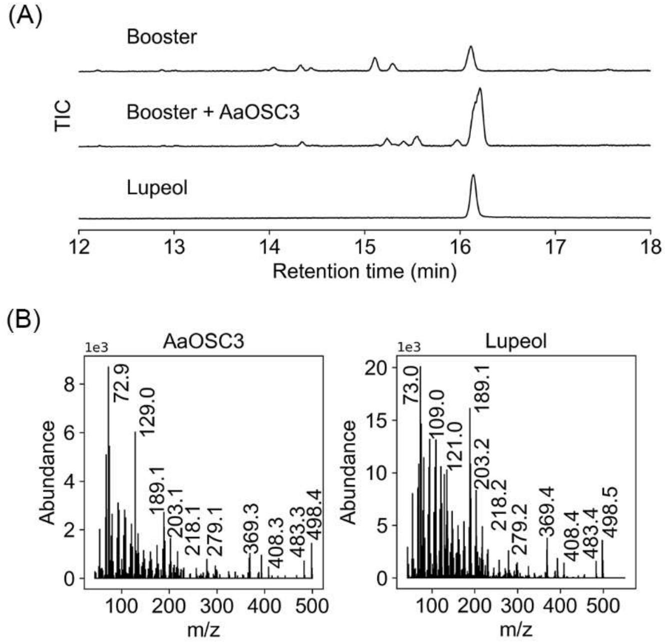

Supplementary Figure 4. GCMS analysis (total ion chromatograms) of *AaOSC3* expression in *N. benthamiana* and lupeol standard show matching fragmentation pattern and retention time. The major fragments ions (Carvalho et al., 2010) from lupeol-TMS ether *m*/*z* 483, 279, 189 are observed in the AaOSC3 product peak.

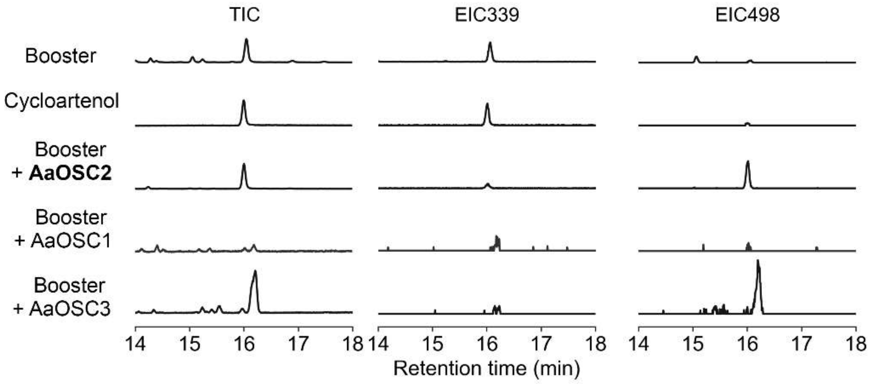

Supplementary Figure 5. Extracted ion chromatograms at *m*/*z* 498 and *m*/*z* 339 for transient expression of AaOSCs in *N. benthamiana* reveal a cryptic product for AaOSC2, which co-elutes with cycloartenol at 16.0 min but has a different mass spectrum.

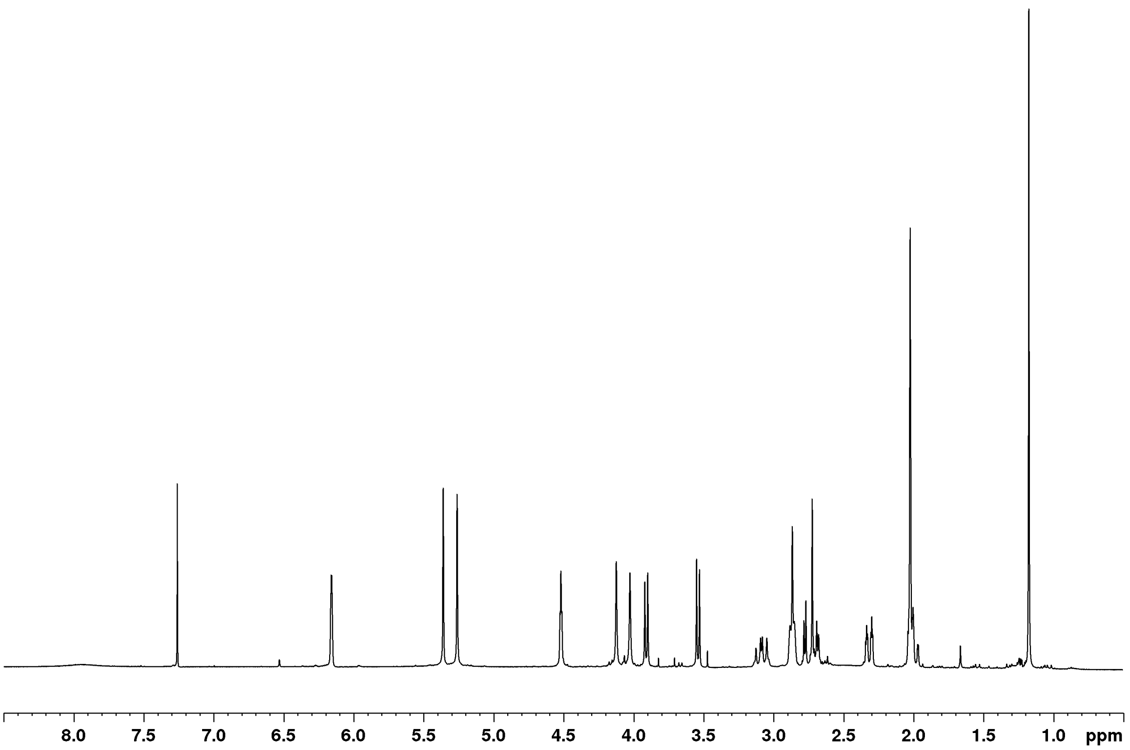

Supplementary Figure 6. ^{1}H NMR spectrum of ailanthone ($CDCl_3$, 298 K, 400 MHz).

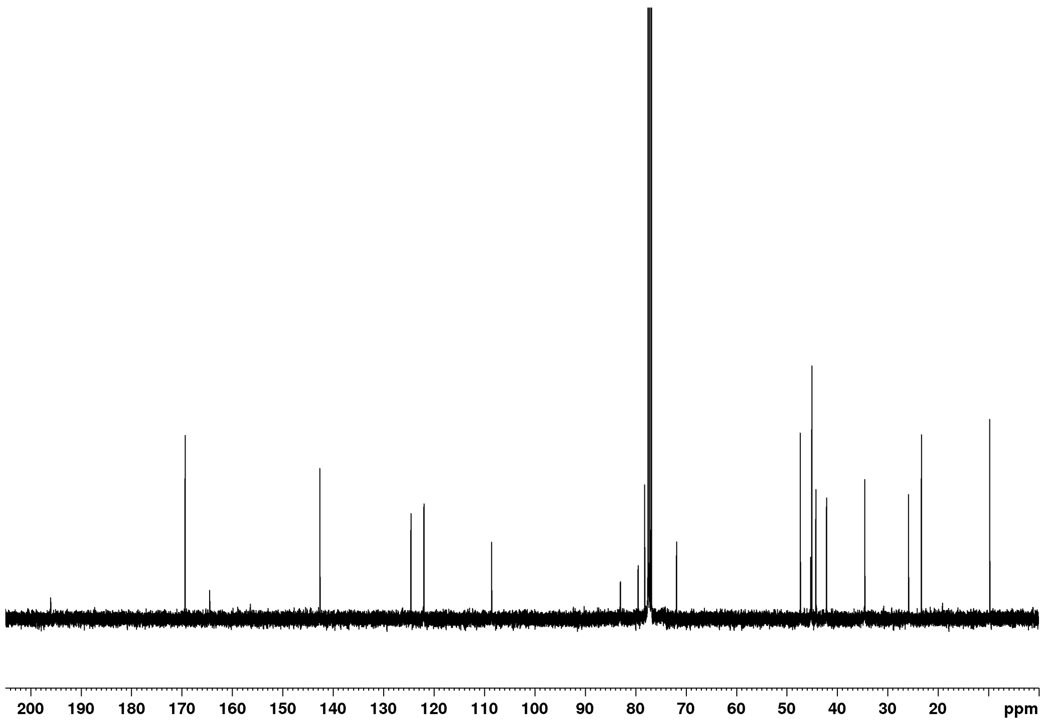

Supplementary Figure 7. ^{13}C NMR spectrum of ailanthone ($CDCl_3$, 298 K, 100 MHz).

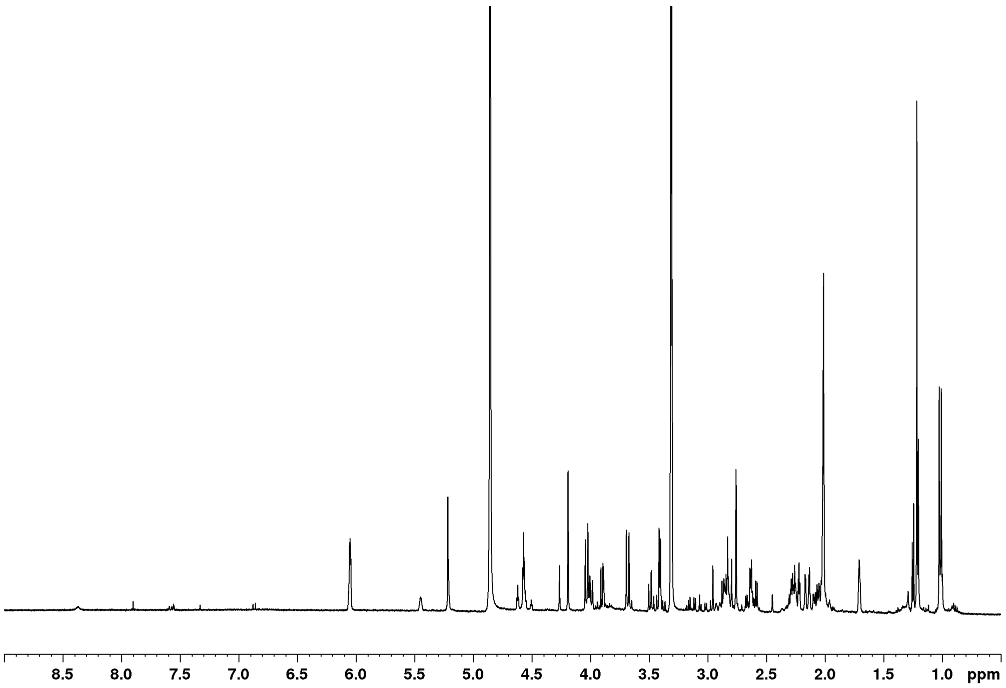

Supplementary Figure 8. ^{1}H NMR spectrum of chaparrinone (CD_3OD, 298 K, 400 MHz).

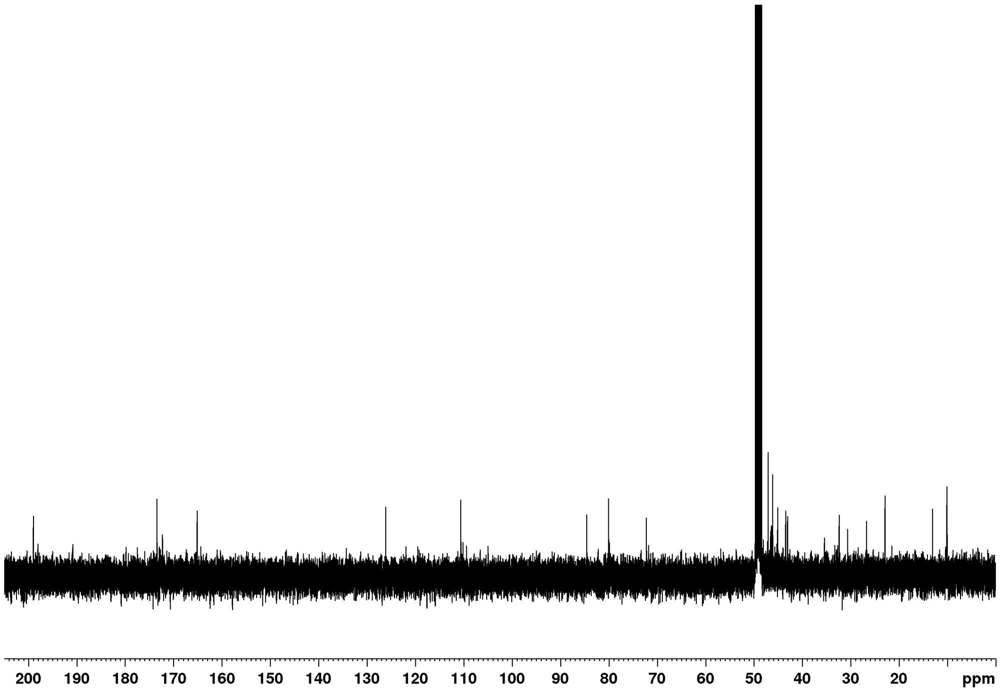

Supplementary Figure 9. ^{13}C NMR spectrum of chaparrinone (CD_3OD, 298 K, 100 MHz).

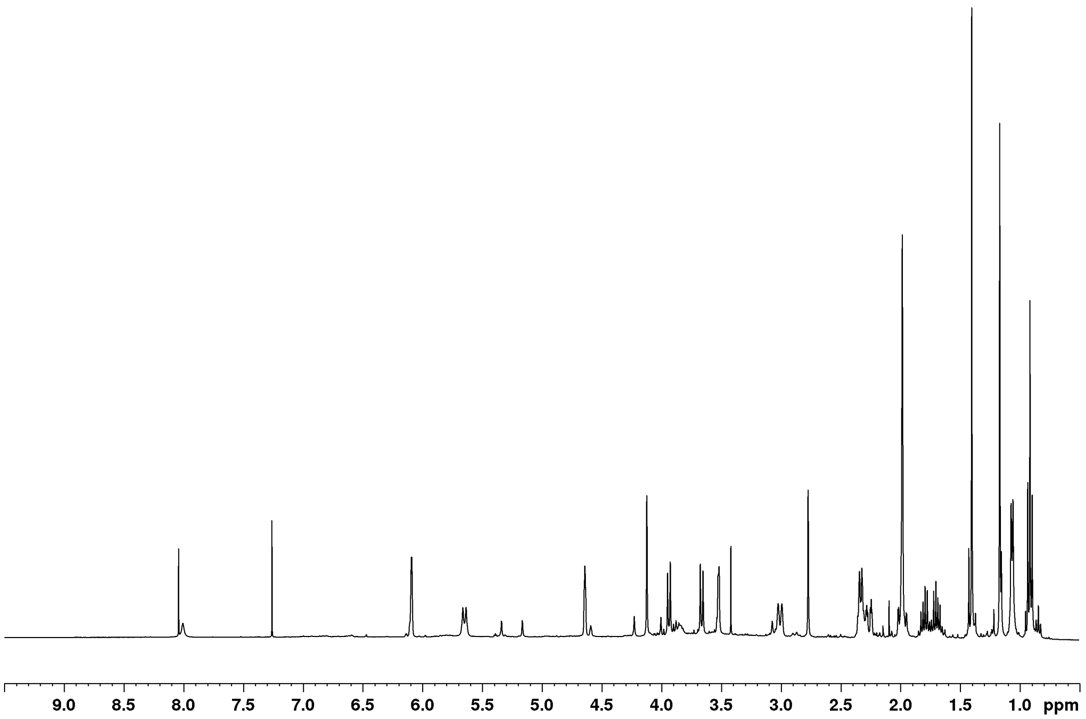

Supplementary Figure 10. ^{1}H NMR spectrum of glaucarubinone ($CDCl_3$, 298 K, 400 MHz).

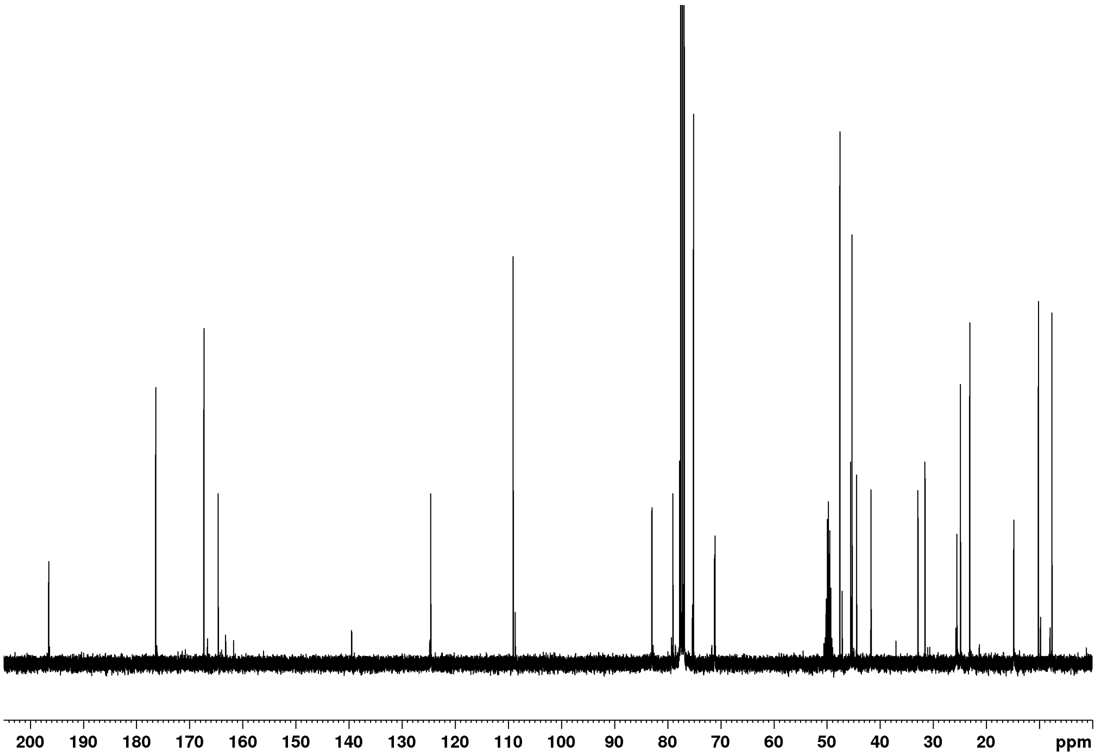

Supplementary Figure 11. ^{13}C NMR spectrum of glaucarubinone ($CDCl_3$, 298 K, 100 MHz).

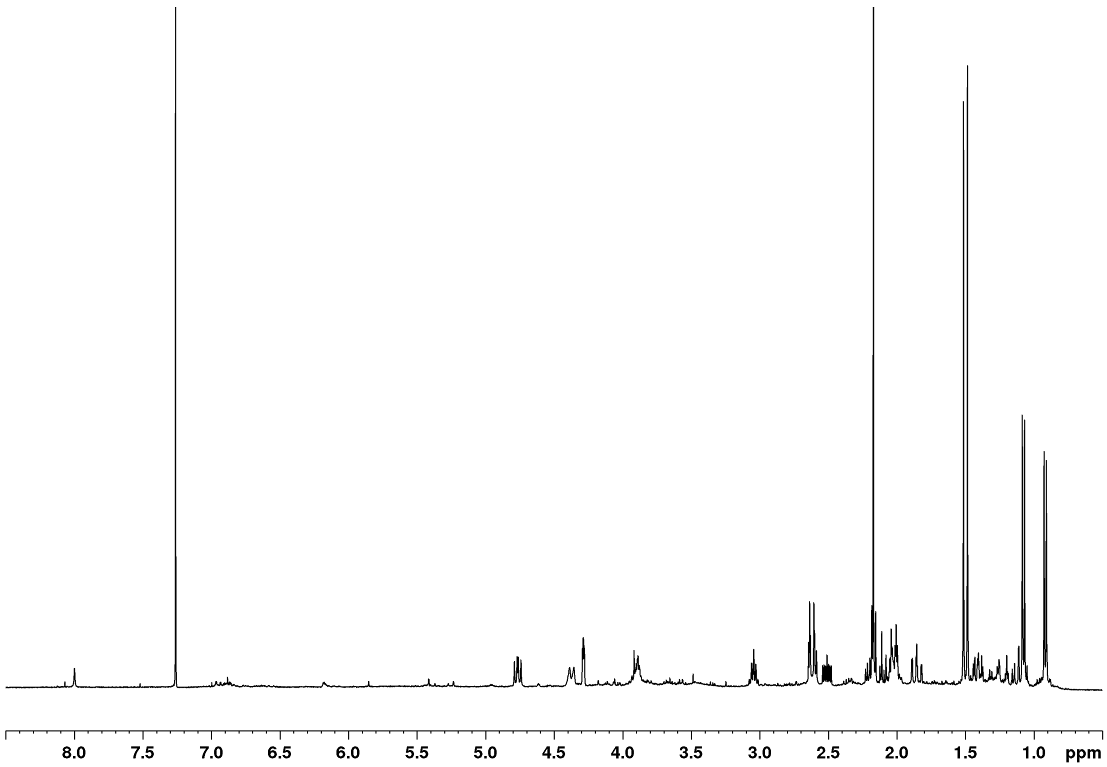

Supplementary Figure 12. ^{1}H NMR spectrum of amarolide ($CDCl_3$, 298 K, 400 MHz).

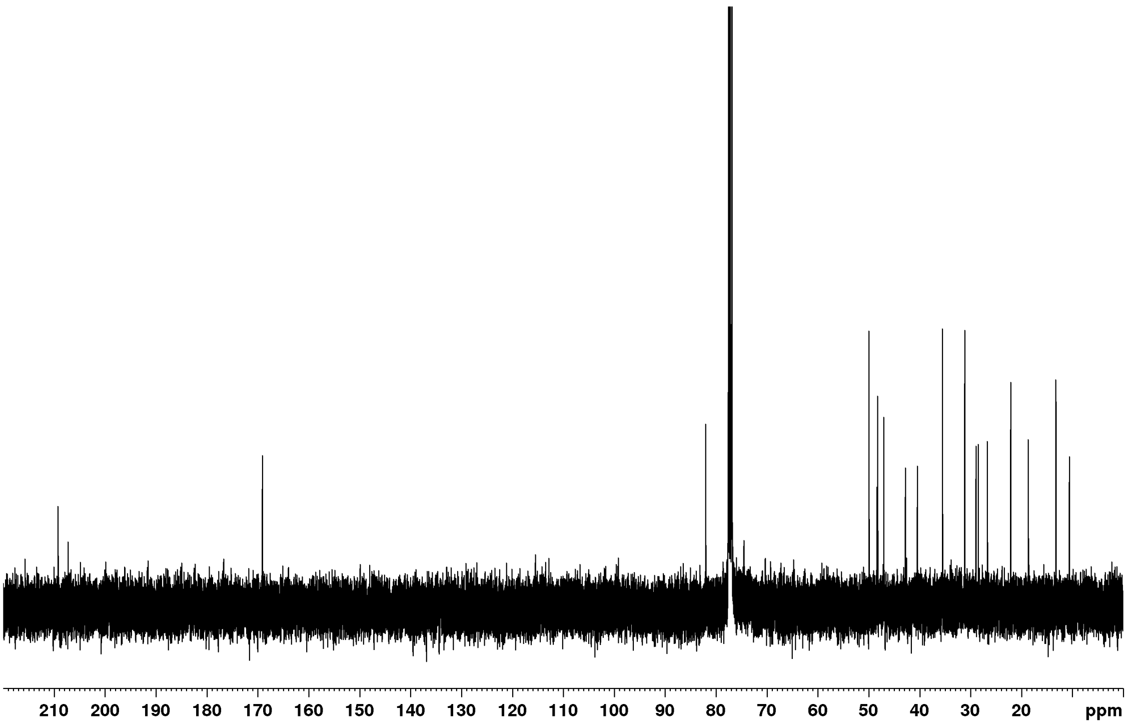

Supplementary Figure 13. ^{13}C NMR spectrum of amarolide ($CDCl_3$, 298 K, 100 MHz).

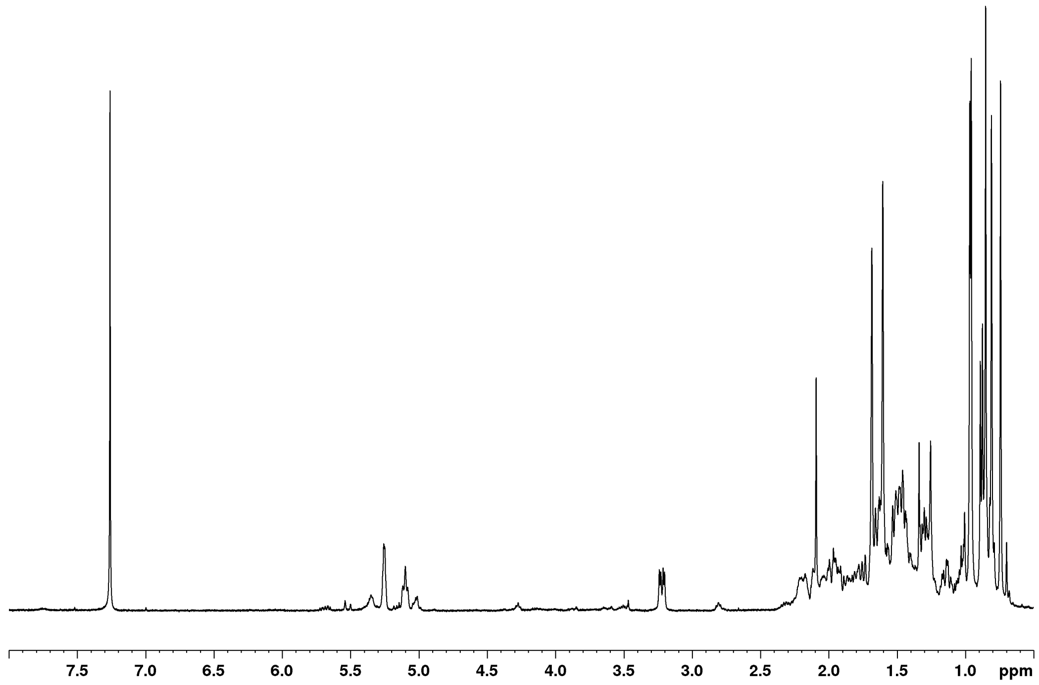

Supplementary Figure 14. ^{1}H NMR spectrum of tirucalla-7,24-dien-3β-ol ($CDCl_3$, 298 K, 400 MHz).

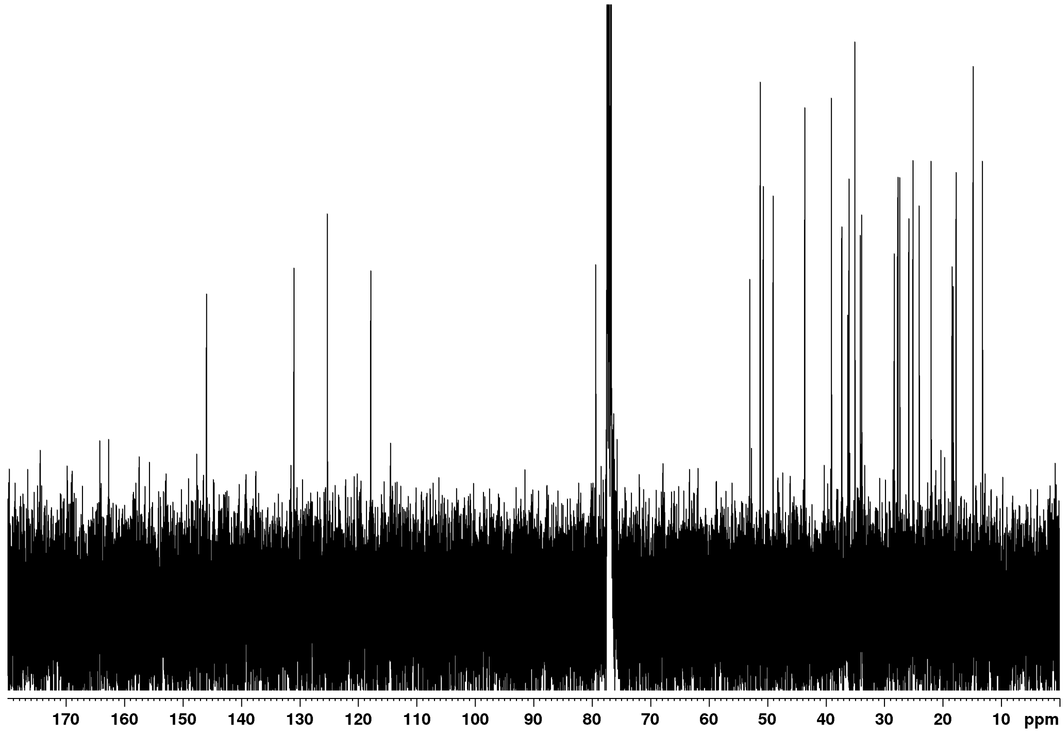

Supplementary Figure 15. ^{13}C NMR spectrum of tirucalla-7,24-dien-3β-ol ($CDCl_3$, 298 K, 100 MHz).

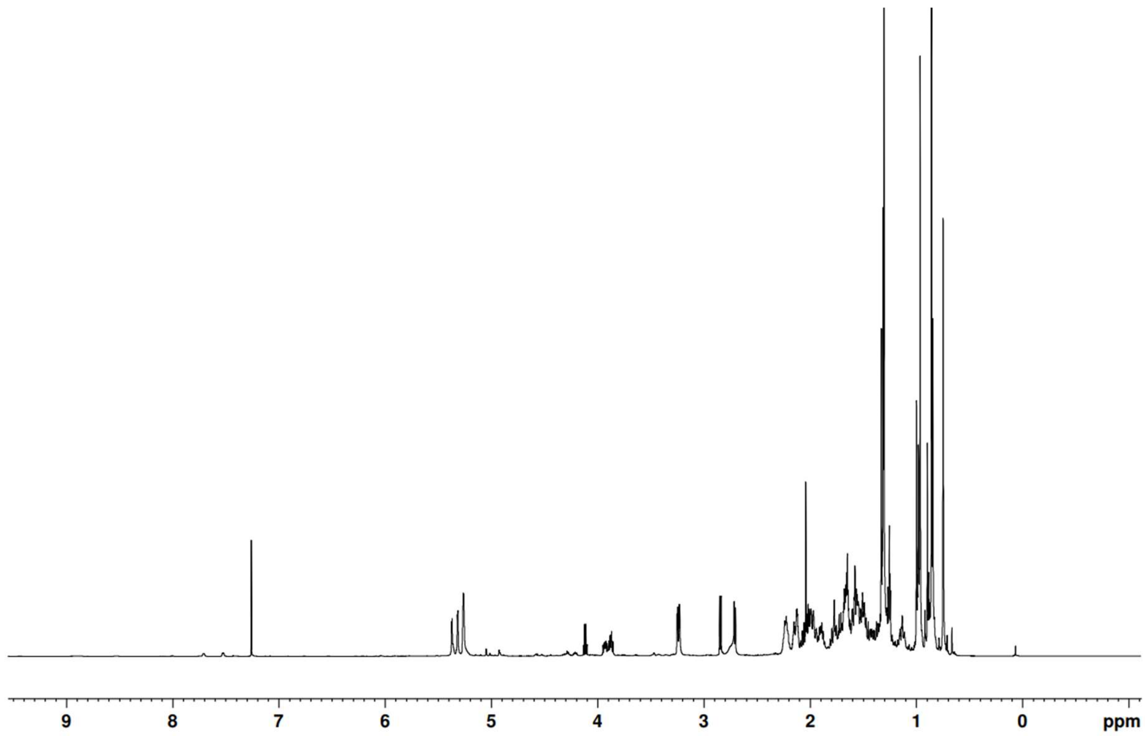

Supplementary Figure 16. ^{1}H NMR spectrum of melianol ($CDCl_3$, 298 K, 600 MHz).

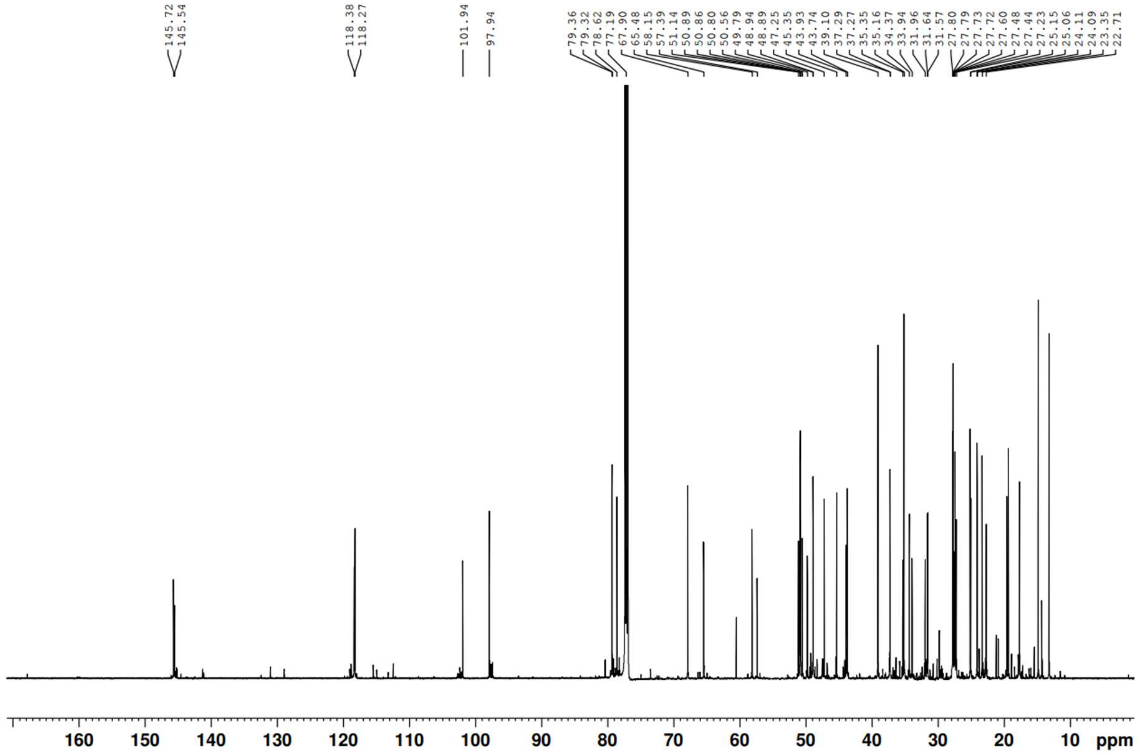

Supplementary Figure 17. ^{13}C NMR spectrum of melianol ($CDCl_3$, 298 K, 150 MHz).

Supplementary Table 2. NMR data of ailanthone ($CDCl_3$, 298 K, 400 MHz) in comparison to literature data.

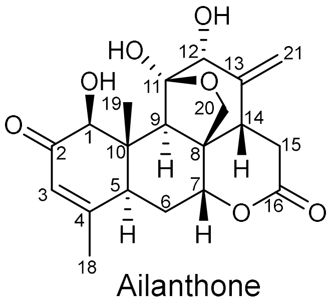

Ailanthone

**Atom**	**¹H ppm (m, Hz)**	**Reference ¹H (Lin et al., 1995)**	**¹³C ppm**	**Reference ¹³C (Lin et al., 1995)**
1	4.12 (1H, s)	4.11 (1H, s)	82.99	82.84
2	-	-	196.00	195.90
3	6.16 (1H, m)	6.12 (1H, br s)	124.53	124.36
4	-	-	164.46	164.25
5	2.87 (1H, br d, 11.7)	2.84 (1H, m)	42.08	41.90
6	2.32 (1H, dt, 14.6, 2.7)<br>2.00 (1H, ddd, 14.7, 12.7, 2.6)	2.28 (1H, m)<br>2.02 (1H, m)	25.80	25.62
7	4.52 (1H, t, 2.6)	4.49 (1H, t, 2.7)	78.15	77.99
8	-	-	45.21[a]	44.81
9	2.86 (1H, s)	2.84 (1H, s)	44.18	43.98
10	-	-	44.98[a]	45.00
11	-	-	108.53	108.43
12	4.03 (1H, s)	3.99 (1H, s)	79.46	79.34
13	-	-	142.58	142.52
14	2.70 (1H, m)	2.69 (1H, m)	47.27	47.08
15	3.09 (1H, dd, 18.1, 12.9)<br>2.73 (1H, dd, 18.1, 5.6)	3.06 (1H, dd, 15.2, 5.2)<br>2.70 (1H, dd, 15.2, 5.6)	34.49	34.30
16	-	-	169.33	169.23
18	2.02 (3H, br s)	1.99 (3H, s)	23.24	23.05
19	1.17 (3H, s)	1.14 (3H, s)	9.70	9.51
20	3.91 (1H, d, 8.5)<br>3.54 (1H, d, 8.5)	3.88 (1H, d, 8.5)<br>3.50 (1H, d, 8.6)	71.83	71.64
21	5.36 (1H, s)<br>5.26 (1H, s)	5.32 (1H, s)<br>5.22 (1H, s)	121.95	121.63

[a] Differing assignments supported by HMBC correlations.

Supplementary Table 3. NMR data of chaparrinone (CD_3OD, 298 K, 400 MHz) in comparison to literature data.

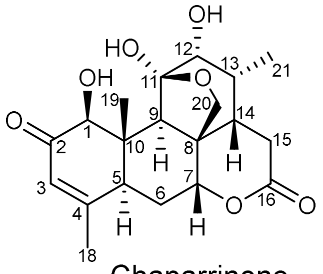

Chaparrinone

Atom	^{1}H ppm (m, Hz)	Reference ^{1}H $CDCl_3$:CD_3OD 10:1 (Lin et al., 1995)	^{13}C ppm	Reference ^{13}C $CDCl_3$:CD_3OD 10:1 (Lin et al., 1995)
1	4.19 (1H, s)	4.07 (1H, s)	84.53	82.94
2	-	-	198.95	196.42
3	6.05 (1H, m)	6.15 (1H, br s)	126.08	124.52
4	-	-	165.09	164.13
5	2.85 (1H, m)	2.85 (1H, m)	43.37	41.97
6	2.24 (1H, dt, 15.0, 2.9)<br>2.13 (1H, ddd, 15.0, 12.6, 2.5)	2.30 (1H, m)<br>2.02 (1H, m)	26.68	25.53
7	4.57 (1H, t, 2.8)	4.48 (1H, br s)	80.01[b]	78.11
8	-	-	47.02	44.83
9	2.76 (1H, s)	2.71 (1H, s) or 2.01 (1H, m)[a]	45.04	43.67
10	-	-	46.08	45.45
11	-	-	110.57	108.90
12	3.41 (1H, d, 4.0)	3.56 (1H, d, 4.4)	80.03[b]	78.39
13	2.30-2.25 (1H, m)	2.30 (1H, m)	32.33	30.50
14	2.08-2.02 (1H, m)	Not reported[a]	43.01	41.97
15	2.84 (1H, m)<br>2.61 (1H, m)	2.79 (1H, m)<br>2.65 (1H, m)	30.58	29.60
16	-	-	173.39	170.81
18	2.01 (3H, m)	2.02 (3H, s)	22.85	22.80
19	1.22 (3H, s)	1.19 (3H, s)	10.07	9.46
20	4.03 (1H, d, 8.7)<br>3.68 (1H, d, 8.8)	3.96 (1H, d, 8.7)<br>3.68 (1H, d, 8.8)	72.19	70.95
21	1.01 (3H, d, 7.2)	1.05 (3H, d, 7.1)	13.02	12.30

[a] No shift reported for H-14, but H-9 reported twice (2.71 and 2.01 ppm); one of them likely is H-14.
[b] Signals interchangeable.

Supplementary Table 4. NMR data of glaucarubinone ($CDCl_3$, 298 K, 400 MHz) in comparison to literature data.

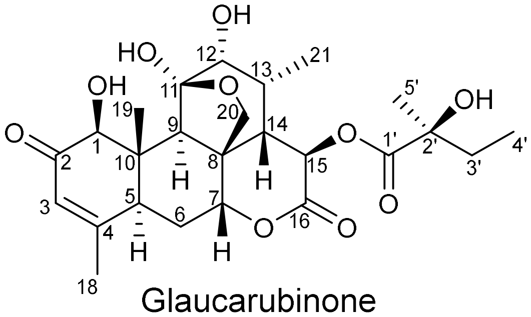

Glaucarubinone

Atom	^{1}H ppm (m, Hz)	^{13}C ppm	Reference ^{13}C (Grieco et al., 1993) [a]
1	4.12 (1H, s)	82.91	82.90
2	-	196.49	196.02
3	6.09 (1H, m)	124.56	124.29
4	-	164.58	164.54
5	3.01 (1H, br d, 12.6)	41.66	41.59
6	2.26 (1H, dt, 14.9, 3.0)<br>1.98 (1H, m)	25.48	25.42
7	4.64 (1H, t, 2.6)	77.69	77.49
8	-	47.52	47.41
9	2.77 (1H, s)	44.37	44.38
10	-	45.23	45.19
11	-	109.03	108.86
12	3.52 (1H, m)	78.95	79.08
13	2.36-2.30 (1H, m)	31.49	31.31
14	2.37-2.30 (1H, m)	45.49	45.46
15	5.65 (1H, d, 11.0)	71.02[b]	70.91[b]
16	-	167.25	166.80
18	1.98 (3H, br s)	23.03	22.99
19	1.17 (3H, s)	10.12	10.03
20	3.94 (1H, d, 9.0)<br>3.66 (1H, d, 9.0)	71.11[b]	71.09[b]
21	1.07 (3H, d, 6.5)	14.75	14.67
1‘	-	176.33	Not reported
2‘	-	75.07	74.97
3‘	1.80 (1H, dq, 14.7, 7.3)<br>1.69 (1H, dq, 14.5, 7.5)	32.81	32.87
4‘	0.91 (3H, t, 7.4)	7.59	7.47
5‘	1.40 (3H, s)	24.80	24.79

[a] Reported shifts were not assigned to atoms. Literature data was assigned to correspond to our 2D NMR data.
[b] Signals interchangeable.

Supplementary Table 5. NMR data of amarolide ($CDCl_3$, 298 K, 400 MHz) in comparison to literature data.

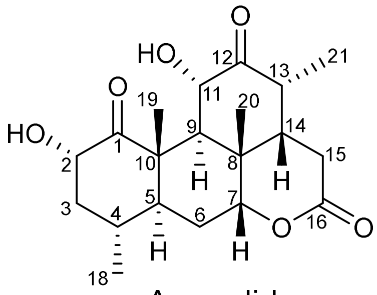

Amarolide

Atom	^{1}H ppm (m, Hz)	Reference ^{1}H (Hirota et al., 1991) [a]	^{13}C ppm
1	-	-	215.67
2	4.76 (1H, dd, 11.2, 7.8)	4.77 (1H, ddd, 11.5, 7.5, 6.0)	70.28
3	2.51 (1H, ddd, 12.8, 8.0, 4.5)<br>1.16-1.05 (1H, m)	Not reported	48.32[b]
4	2.04-1.98 (1H, m)	Not reported	28.85
5	1.44-1.37 (1H, m)	Not reported	47.00
6	2.05-1.99 (1H, m)<br>1.85 (1H, ddd, 14.8, 12.9, 2.0)	Not reported	26.63
7	4.28 (1H, dd, 3.6, 2.1)	4.29 (1H, dd, 3.5, 2.5)	81.95
8	-	-	35.45
9	2.62 (1H, d, 12.5)	2.62 (1H, d, 12.5)	40.35
10	-	-	49.89
11	4.37 (1H, br d, 12.3)	4.38 (1H, dd, 12.5, 10.0)	74.47
12	-	-	209.23
13	3.04 (1H, dq, 6.8, 6.3)	3.05 (1H, m)	42.73
14	2.23-2.15 (1H, m)	Not reported	48.17[b]
15	2.61 (1H, dd, 17.6, 5.4)<br>2.13 (1H, m)	Not reported	28.42
16	-	-	169.06
18	0.91 (3H, d, 6.5)	0.92 (3H, d, 7.0)	18.60
19	1.48 (3H, s)	1.49 (3H, s)	13.16
20	1.51 (3H, s)	1.52 (3H, s)	22.04
21	1.07 (3H, d, 6.7)	1.08 (3H, d, 7.5)	10.52

[a] Reported shifts were only partially assigned to atoms. Unassigned literature data was assigned to correspond to our 2D NMR data.
[b] Signals interchangeable.

Supplementary Table 6. NMR data of tirucalla-7,24-dien-3β-ol ($CDCl_3$, 298 K, 400 MHz) in comparison to literature data.

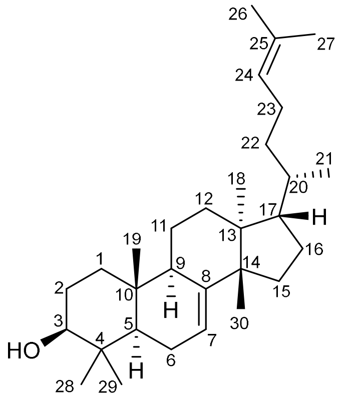

Tirucalla-7,24-dien-3β-ol

Atom	^{1}H ppm (m, Hz)	Reference ^{1}H (Hodgson et al., 2019)	^{13}C ppm	Reference ^{13}C (Hodgson et al., 2019)
1	1.64-1.71, 1.09-1.15 (2H, m)	1.68 (1H, m), 1.14 (1H, m)	37.35	37.21
2	1.56-1.67 (2H, m)	1.64 (2H, m)	27.82[a]	27.70
3	3.22 (1H, dd, 11.0, 4.5)	3.25 (1H, dd, 11.2, 4.3)	79.43	79.27
4	-	-	39.11	38.96
5	1.27-1.33 (1H, m)	1.32 (1H, m)	50.76	50.63
6	2.09-2.16, 1.94-2.00 (2H, m)	2.14 (1H, m), 1.96 (1H, m)	24.09	23.94
7	5.25 (1H, dt, 5.9, 2.9)	5.25 (1H, m)	117.94	117.80
8	-	-	146.05	145.90
9	2.16-2.22 (1H, m)	2.20 (1H, m)	49.10	48.95
10	-	-	35.09	34.95
11	1.46-1.53 (2H, m)	1.51 (2H, m)	18.28	18.13
12	1.74-1.80, 1.58-1.63 (2H, m)	1.79 (1H, m), 1.61 (1H, m)	33.95	33.79
13	-	-	43.66	43.52
14	-	-	51.30	51.15
15	1.40-1.49 (2H, m)	1.45 (2H, m)	34.18	34.03
16	1.89-1.97, 1.30-1.23 (2H, m)	1.93 (1H, m), 1.27 (1H, m)	28.36	28.22
17	1.44-1.48 (1H, m)	1.47 (1H, m)	53.10	52.95
18	0.81 (3H, s)	0.81 (3H, s)	22.06	21.91
19	0.74 (3H, s)	0.75 (3H, s)	13.26	13.12
20	1.34-1.37 (1H, m)	1.36 (1H, m)	36.11	35.97
21	0.88 (3H, d, 6.3)	0.88 (3H, d, 6.4)	18.48	18.33
22	1.41-1.48, 0.99-1.08 (2H, m)	1.46 (1H, m), 1.03 (1H, m)	36.33	36.19
23	1.99-2.07, 1.84-1.90 (2H, m)	2.04 (1H, m), 1.86 (1H, m)	25.17	25.02
24	5.06-5.13 (1H, t, 7.2)	5.01 (1H, appt tquin, 7.1, 1.4)	125.36	125.22
25	-	-	131.09	130.94
26	1.60 (3H, s)	1.60 (3H, s)	17.80	17.64
27	1.68 (3H, s)	1.68 (3H, s)	25.89	25.73
28	0.95 (3H, s)	0.97 (3H, s)	27.74[ab]	27.61
29	0.85 (3H, s)	0.86 (3H, s)	14.87	14.73
30	0.97 (3H, s)	0.97 (3H, s)	27.41[b]	27.27

[a] Signals interchangeable.

[b] Signals interchangeable.

Supplementary Table 7. Conditions for flash chromatography purification of melianol.

Target Molecule	Column	Solvents	Gradient	Yield
Melianol	SNAP KP-Sil 100 g	A: Petroleum ether<br>B: Ethyl Acetate	6-100% (13 CV)	1591 mg
	SNAP KP-Sil 50 g	A: Petroleum ether<br>B: Ethyl Acetate	10-75% (13 CV)<br>75% (2 CV)	568 mg
	SNAP Ultra 10 g	A: Dichloromethane<br>B: Ethyl Acetate	0-10% (20 CV)<br>10% (20 CV)<br>10%-100% (5 CV)	210 mg
	Sfär C18 D 12 g	A: Water<br>B: Acetonitrile	80% (10 CV)<br>80-100% (2 CV)<br>100% (3 CV)	67 mg

Supplementary Table 8. NMR data of melianol ($CDCl_3$, 298 K, 600 MHz). Melianol occurs as a ca. 2:1 mixture of C-21 epimers. The first values correspond to the major epimer, the second values to the minor epimer. Assignments deviating from literature are supported by 2D correlations.

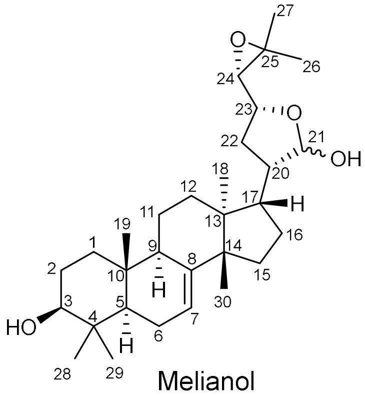

Atom	^{1}H ppm (m, Hz)	^{13}C ppm	Reference ^{13}C (Hodgson et al., 2019)
1	1.07-1.16, 1.63-1.70 (2H, m)	37.29/37.27	37.17/37.15
2	1.55-1.69 (2H, m)	27.80/27.79	27.70
3	3.24 (1H, dd, 11.5, 4.0)	79.36/79.32	79.25/79.21
4	-	39.10	38.99
5	1.27-1.34 (1H, m)	50.86/50.80	50.78/50.75
6	1.92-2.00, 2.10-2.17 (2H, m)	24.11/24.09	23.23
7	5.26 (1H, br s)	118.27/118.38	118.28/118.18
8	-	145.72/145.54	145.59/145.42
9	2.19-2.26 (1H, m)	48.94/48.89	49.71/48.83
10	-	35.16	35.05
11	1.44-1.59 (2H, m)	17.68/17.67	17.56/17.55
12	1.66-1.80 (2H, m)	31.64/31.96	35.24
13	-	43.74/43.93	43.82/43.63
14	-	50.89/51.14	50.68/50.44
15	1.46-1.69 (2H, m)	34.37/33.94	34.25
16	1.27-1.37, 1.86-1.93 (2H, m)	27.48/27.60	27.37/27.12
17	2.01-2.09/1.76-1.81 (1H, m)	45.35/50.56	47.15/45.25
18	0.84/0.90 (3H, s)	23.35/22.71	13.08
19	0.75 (3H, s)	13.20/13.19	23.99/23.98
20	1.98-2.05/2.19-2.26 (1H, m)	47.25/49.79	33.83/31.87
21	5.32 (1H, d, 3.3) / 5.37 (1H, d, 3.2)	97.94/101.94	101.86/97.86
22	1.36-1.47, 1.93-2.04 (2H, m)	31.57/35.35	31.52/31.47
23	3.88 (1H, dt, 9.6, 7.0) / 3.93 (1H, ddd, 10.6, 7.6, 5.3)	78.62/77.19	78.53/77.12
24	2.84 (1H, d, 7.5) / 2.71 (1H, d, 7.6)	67.90/65.48	67.76/65.34
25	-	58.15/57.39	58.03/57.25
26	1.31/1.33 (3H, s)	25.15/25.06	25.05/24.95
27	1.30 (3H, s)	19.35/19.58	19.46/19.22
28	0.96 (3H, s)	27.73	27.48/27.61
29	0.86 (3H, s)	14.85	14.72
30	1.00/0.98 (3H, s)	27.44/27.23	22.71/22.60

Supplementary Table 9. List of primer sequences used in this study. In the primer names, F is short for forward primer and R is short for reverse primer. For RT-PCR of AaOSC1, 2 and 3, primer for cloning were used. Start and stop codons are marked in red. Overlapping sequences to the vector for In-Fusion cloning are marked in blue. For Golden gate cloning, BsaI restriction sites are marked in green, and the cutting site is labeled with a slash.

Primer name	Sequence	Purpose
AaOSC1_cloning_F	CAAATTCGCGACCGGATGTGGAGACTGAAGATTGCAGA	Cloning of AaOSC1 to pEAQ-HT (AgeI/XhoI cut) using In-Fusion
AaOSC1_cloning_R	AGTTAAAGGCCTCGATCAAGGCAATGGAACTTTGCTG	
AaOSC2_cloning_F	CACCACAGGTCTCG/AAAAATGTGGAGGCTTAAGATTGCAGA	Cloning of AatHMGR to pHREAC using Golden gate (BsaI)
AaOSC2_cloning_R	CACCACAGGTCTCG/AGCGTCAATTAGGCAATGGAACTTTCCT	
AaOSC3_cloning_F	GCCCAAATTCGCGACCGGTTGTATTTGAAAGAGAGAAAAGAAGTGA	Cloning of AaOSC3 to pEAQ-HT (AgeI/XhoI cut) using In-Fusion
AaOSC3_cloning_R	CAGAGTTAAAGGCCTCGACTAAAATTTGCTGGAAGGGAATGGA	
AaCYP71CD4_cloning_F	GCCCAAATTCGCGACCGGATGATGGAGCTACAGCTTGA	Cloning of AaCYP71CD4 to pEAQ-HT (AgeI/XhoI cut) using In-Fusion
AaCYP71CD4_cloning_R	CAGAGTTAAAGGCCTCGATCACGGATCGTAAGGAGTGG	
AaCYP71BQ17_cloning_F	GCCCAAATTCGCGACCGGTTGAGAACAAAATTGCCAATGGA	Cloning of AaCYP71BQ17 to pEAQ-HT (AgeI/XhoI cut) using In-Fusion
AaCYP71BQ17_cloning_R	CAGAGTTAAAGGCCTCGATCACTTCTGGAAAGGAATATGAGTG	
AaSQS_cloning_F	TAAACGTCTCTAAAAATGTTGAGATCAGAAAGAGAGAGAACAA	Cloning of AaSQS to pHREAC (BsaI cut) using In-Fusion
AaSQS_cloning_R	AATGAAACCAGAGCGCTAGTTAGTCAGTCGGTTGGAG	
AatHMGR_cloning_F	CACCACAGGTCTCG/AAAAATGGATCCCACCACGGTGTC	Cloning of AatHMGR to pHREAC using Golden gate (BsaI)
AatHMGR_cloning_R	CACCACAGGTCTCG/AGCGTTAAACAAGCTGGCCAGCAG	
AaIPPI_cloning_F	CACCACAGGTCTCG/AAAAATGTCTGCTCTTTATAATCTCACAACC	Cloning of AaIPPI to pHREAC using Golden gate (BsaI)
AaIPPI_cloning_R	CACCACAGGTCTCG/AGCGCTAAGTTAACTTGTGAATGATTTGCA	
AaFPS_cloning_F	CACCACAGGTCTCG/AAAAATGAGTGATCTGAAGGCAAGA	Cloning of AaFPS to pHREAC using Golden gate (BsaI)
AaFPS_cloning_R	CACCACAGGTCTCG/AGCGTTACTTCTGCCTCTTGTATATCTTTGC	
Candidate_1_cloning_F	GCCCAAATTCGCGACCGGCTGATCTCAAGGTGGAGAAACA	Cloning of TRINITY_DN1187_c0_g1_i6 to pEAQ-HT (AgeI/XhoI cut) using In-Fusion
Candidate_1_cloning_R	CAGAGTTAAAGGCCTCGATCATGCTTTTTCTGATGGCTGC	
Candidate_2_cloning_F	CACCACAGGTCTCG/AAAATTGTCAACAAAAGCTGAAATGGC	Cloning of TRINITY_DN6765_c

Candidate_2_cloning_R	CACCACAGGTCTCG/AGCGCTAAGCTTGGGCTTTCTTCACC	0_g1_i9 to pHREAC using Golden gate (BsaI)
Candidate_3_cloning_F	GCCCAAATTCGCGACCGGATGGAAGAAACAACCATTCTGT	Cloning of TRINITY_DN9749_c0_g1_i3 to pEAQ-HT (AgeI/XhoI cut) using In-Fusion
Candidate_3_cloning_R	CAGAGTTAAAGGCCTCGATTAGCTCCACACAGAACCTCC	
Candidate_5_cloning_F	CACCACAGGTCTCG/AAAAATGAGCACCCCATCCATTAT	Cloning of TRINITY_DN6765_c0_g1_i2 to pHREAC using Golden gate (BsaI)
Candidate_5_cloning_R	CACCACAGGTCTCG/AGCGTTAAGCTTGGACTTTCTTCAACGA	
Candidate_6_cloning_F	GCCCAAATTCGCGACCGGCTGATGCACAAAGCACAATCTCT	Cloning of TRINITY_DN9765_c0_g1_i2 to pEAQ-HT (AgeI/XhoI cut) using In-Fusion
Candidate_6_cloning_R	CAGAGTTAAAGGCCTCGATTAAAAAAGAAAGTTCATGATGGGTCT	
Candidate_7_cloning_F	GCCCAAATTCGCGACCGGTTGAGAAAGTGCATTAATTTCAAGCA	Cloning of TRINITY_DN4730_c0_g1_i9 to pEAQ-HT (AgeI/XhoI cut) using In-Fusion
Candidate_7_cloning_R	CAGAGTTAAAGGCCTCGATCAATTGATATTTCTAGAATGATAAGGACTGG	
Candidate_9_cloning_F	GCCCAAATTCGCGACCGGATGGAGTTGTTTGAATCATCCT	Cloning of TRINITY_DN12394_c0_g1_i3 to pEAQ-HT (AgeI/XhoI cut) using In-Fusion
Candidate_9_cloning_R	CAGAGTTAAAGGCCTCGATCAGTCTCTTCTTTCTTGAACCAC	
Candidate_10_cloning_F	CACCACAGGTCTCG/AAAAATGGAACTCCTCCTTTACACA	Cloning of TRINITY_DN185182_c0_g1_i1 to pHREAC using Golden gate (BsaI)
Candidate_10_cloning_R	CACCACAGGTCTCG/AGCGTTAATTTTCATTGGGAAAAAGGCGA	
AaACT1_RT-PCR_F	TTGACTTTGAAGTACCCAATTGAACATG	For RT-PCR of AaACT1 (scaffold-QICX-2036794-Ailanthus_altissima)
AaACT1_RT-PCR_R	TCAGAAGCATTTCCTGTGCACAATC	

Supplementary References

Carvalho, T. C. de, Polizeli, A. M., Turatti, I. C. C., Severiano, M. E., Carvalho, C. E. de, Ambrósio, S. R., et al. (2010). Screening of Filamentous Fungi to Identify Biocatalysts for Lupeol Biotransformation. *Molecules* 15, 6140–6151. doi: 10.3390/molecules15096140.

Grieco, P. A., Collins, J. L., Moher, E. D., Fleck, T. J., and Gross, R. S. (1993). Synthetic studies on quassinoids: total synthesis of (-)-chaparrinone, (-)-glaucarubolone, and (+)-glaucarubinone. *J. Am. Chem. Soc.* 115, 6078–6093. doi: 10.1021/ja00067a025.

Hirota, H., Yokoyama, A., Miyaji, K., Nakamura, T., Igarashi, M., and Takahashi, T. (1991). Total synthesis of (.+-.)-amarolide, a quassinoid bitter principle. *J. Org. Chem.* 56, 1119–1127. doi: 10.1021/jo00003a039.

Hodgson, H., Peña, R. D. L., Stephenson, M. J., Thimmappa, R., Vincent, J. L., Sattely, E. S., et al. (2019). Identification of key enzymes responsible for protolimonoid biosynthesis in plants: Opening the door to azadirachtin production. *Proc. Natl. Acad. Sci. U. S. A.*, 201906083. doi: 10.1073/pnas.1906083116.

Lin, L.-J., Peiser, G., Ying, B.-P., Mathias, K., Karasina, F., Wang, Z., et al. (1995). Identification of Plant Growth Inhibitory Principles in Ailanthus altissima and Castela tortuosa. *J. Agric. Food Chem.* 43, 1708–1711. doi: 10.1021/jf00054a056.
