# Supplementary material for: Identification of early quassinoid biosynthesis in the invasive tree of heaven (Ailanthus altissima) confirms evolutionary origin from protolimonoids
Source: Front Plant Sci. 2022 Aug 23;13:958138. doi: 10.3389/fpls.2022.958138 (PMC9445810; doi:10.3389/fpls.2022.958138)
Supplement: Supplementary file 2 [file Data_Sheet_2.PDF]

>AaOSC1_TRINITY_DN3249_c0_g2_i2 len=2482 path=[0:0-1362 2:1363-1584 4:1585-2481]

ACCAATATAAATGTGGAGACTGAAGATTGCAGAGGGTGGCAACAACCCGTATATTTTTAGCACAAACAATTAC
GTGGGAAGGCAAATATGGGAATTTGATCCAAATGATGGCAGCCCTGAGGAGTATGCTGAAGTTGAAGCGGCT
CGCCATAATTTTTACAATAACCGCCGTCAGGTCAAGGCCAGCAGCGACCTTATTTGGCGATTACAGTTTCTGAG
AGAGAAAAACTTCAAGCAAACAATTCCGCAAGTAAAGGTTAAGGATGATGAGGAGATTACATATGAAATTGC
AACGAACGCATTGAGGAGGGCTGTTCACTTCTTTTCAGCGTTACAGGCCAGCGATGGCCATTGGCCTGCTGAA
AATGCTGGACCCTTGTTCTACCTCCCTCCACTTGTTATATGCTTGTACATTACAGGCCATCTCAATATTATATTTA
CAGCCGACCATCGCAAAGAAATCTTTCGCTATTTGTATAATCATCAGAATGAAGATGGTGGGTGGGGATTACA
CGTAGAGGGCCAGAGCACAATGTTTGGTACAGCTTTCAGCTACATTTGCATGCGTTTACTTGGAATGGGACCT
GAAGATGGTGAGAACAATGCTTGTGTAAGAGCACGAAAGTGGATTCTTGACCATGGCGGTGTCACTTACATAC
CTTCTTGGGGAAAGATTTGGCTTTCGATACTTGGACTATTTGATTGGACTGGAACCATCCCAACACCTCCTGAG
TTTTGGCTCCTTCCCTCTTCTCTTCCTTTTCATCCAGCAAAAATGTGGTGCTACGGGCGGCTGCTTTATATGCCCA
CATCTTACTTATATGGGAAAAGGTTCGTTGGTCGAATTACTCCACTTATTCAACAGCTTAGGGAAGAACTCCAC
ACTCAGTCTTACAATGAAATTAATTGGAGGAAAGTGCGTCATTTATGCGCAAAGGAGGATTTGTACCATTCCCA
TCCTTTTATACAAGATGTTTTTTGGGATGTTGTTTACATGACTACGGAGCCTCTGCTATCTCGTTGGCCTTTGAA
CAAGTTGATTAGAGAGAAGGCTCTTCAAGTAACAATGAAGTTCATTCATTATCAAAATGAGAGTAGTCGATAT
ATTACTACTGGATGCATCGAAAAGGCATCATGTATGCTTGCATGTTGGATTGAAGATCCTGAAGGGGATGCCT
TTAAGAAGCATCTTGCCAGAGTCGTAGATTACATTTGGGTCGGAGAAGATGGAATGAAGATTCAGACTTTTGG
CAGTCAAGTATGGGATACTGCTCTTGCCCTTCAAGCTTTGCTTGCTAGCAACCTCATGGATGACATCGGACCTA
CACTTGCTAAAGCACATGATTTCTTGAAAAAATCTCAGATCAAGGATAACCCACCAGGCGATTATAAAAGCATG
TTTCGTCACTTTTCAAAAGGTGCATGGACTTTCTCTGATCAGGATCATGGATTGCAAGTTTCAGATTGTACCGCA
GACAGTTTGAAATGTTGCCTACTTCTCTCAATGCTGCCGCCGGATACTGTTGGTGAGAAGCTAGAAGCTGAGA
AGCTCTACCAGGCTGTCAAATACATACTTTCTGTTCAGAGTGAAGATGGCGGATTAGCTGCTTGGGAGCCAGC
AGGTGGCTCAGTTTTGTTGGAGTGGCTCAGTCCTGTAGAATTTATGAAGGACCTTGTTGTCGAGCAAACGTAT
GTTGAATGCACTGCATCAGCAATCAAAGCATTTGCTTTGTTTAAGAAACTATACCCGAATCACAGGACTAAAGA
GATTGAAAATTCCATCACAAAAGCTGTGAAGTACATTGAAGCCAAACAAGAGGCTGATGGCTCATGGTATGGA
AGTTGGGGAGTGTGCTTCATATATGGTACAGCTTTTGCACTTGGAGGTTTGGCTGCTGCTGGAAAAACTTACA
ACAATTGTCTTGCTATTCGTAGAGCTGTTGGTTTTTTACTGAATACACAAAAGACTGATGGTGGTTGGGGTGAG
AGCTTTCTTTCGTGCCCAACTAAGAAATATACTCCTCTCGAAGGAAAGAGAACAAATCTGGTACAAACTGCATT
GGCTGTTATGGGTTTAATTGATTCTGGACAGGCTGAAAGAGATCCAACCCCTATACATCGTGCTGCAAAGTTGC
TGATCAATTCTCAATTAGAAAATGGAGACTTCCCCCAGGAGCAAATTACGGGAGTTTTCATGAACAGTTGCCCC
TTACACTATGCGGAGTACAGGAATATTTTTCCGTTGTGGGCTTTAGCAGAATACCGCAGCAAAGTTCCATTGCC
TTGATAAAGCTCTTAAATTAATCAAACGACATATCACTCTTAATAAGAATTATGTTTCGATATTTCATGTATTTAT
ACATATTGATCTATTGATTATGTAGTATATGAACGAAAGAGATACTAAAAAATAAATTTGTGCATAAATGATAA
TAATATTCACTTGTTCTTTGTATTGTAAGATTGCATTTGCAAAAGTCCC

>AaOSC2_TRINITY_DN3249_c0_g1_i9 len=2640 path=[0:0-802 2:803-837 3:838-881 4:882-908 6:909-1001 8:1002-1053 9:1054-1620 10:1621-1794 12:1795-1838 14:1839-1899 17:1900-1938 18:1939-1968 21:1969-2021 23:2022-2046 26:2047-2078 28:2079-2150 31:2151-2157 32:2158-2160 33:2161-2252 35:2253-2332 36:2333-2408 38:2409-2420 40:2421-2639]

ATAAATATGATGTTCTATTTTTCTCCTTGAACATACTGCATCGTTTCAAAAAAATCTCTAGCTTTTGCAGCCAACG
ACATCACCAACTCCAACACCAACCCCCTCTTCTTTCCTTGCTCTTGCCAACTGATTCTAAGTCAAGGGGGACGAT
AATATTATTATGTGGAGGCTTAAGATTGCAGAGGGTGACAAAAATAGCCCATACATTTTTACAACAAACAATTT
TGTGGGAAGGCAAATATGGGAATTTGATCCAAATTATGCTGCCTCGCCGGAAGAGCTAGCTGAAGTTGAAGA
GGCTCGCCAGAAGTTTCACAAAAATCGCCACAAGGTCAAGCCTGCCAGTGATCTTATGTGGCGGCTACAGTTC
CTTAGAGAGAAAAACTTCAAGCAAACAATCCCTCCAGTAAAGGTTAAGGATGAGGAGGAAATCACGTATGAA
ACGGCAACCAAGGCAGTGAAGAGGGCTGCTAGCTATTTTTCAGCCATACAGGCTAACGATGGCCACTGGCCTG
CTGAAAATGCTGGCCCTATGTACTTCCTTCCTCCATTTGTCTTCTGCCTGTACATTACAGGGCATCTTGATGCTGT
ATTTACAGCTGAGCACAAAAAAGAAATCCTTCGCTATTTGTACAATCATCAGCATGAAGATGGTGGATGGGGA
ATACACATAGAAGGCCACAGCAGCATGTTTGGCACAGTTTACGGCTACATTACTATGCGTTTACTTGGATTAGG
ACCCAATGATGGTGAAAACAATGCTTGTGCAAGAGCACGAAAATGGATTCGCGACAATGGTGGTGTCACATAC
ATACCCTCCTGGGGAAAGAATTGGCTGTCGATACTTGGATTGTTTGAATGGGCTGGAACCCACCCAATGCCGC
CTGAGTTCTGGCTGCTCCCTTCTTATTTTCCATTGCATCCAGCACAAATGTGGTGCTATTGCCGACTTGTTTACAT
GCCATTATCTTATTTGTATGGGAAAAGATTTGTTGGTCCAATCACTCCGCTTATTCAACAATTGAGGAATGAACT
TCACACTCAGCCGTACAAGGAAATAAATTGGAGGAAAGTTCGTCATTTATGTGCAAAGCCGGATCTCTACTATC
CCCACACCGTGGTACAGAACATACTTTGGGATGGTATGTACATGGCAACAGAGCCTCTCCTAACTCGTTGGCCT
TTGAACAAGTATCTTAGACAGAAGGCTTTAAAAGAAACAATGAAGATCATTCATTATGAAGACCAAAGTAGTA
GATACATTACCATAGGAAGTGTAGAAAAGCCTTTATGTATGCTTGCTTGTTGGGTTGAAGATCCCGATGGTGTT
GCCTTTAAGAAGCATCTTGCTAGAGTTTCAGATTACTTCTGGCTTGGAGAAGATGGAATGAAAGCTCAGACTTT
TGGAAGTCAAACATGGGATACTGCTCTTGGCCTTCAAGCTTTGCTTGCTTGCGATCTCGTCGATGAAATTGCAC
CTACTCTTGCAAAAGGACACGACTACTTAAAGAAAGCTCAGGTGAGGGATAATCCAATAGGCGATTATACAAG
CAATTTCCGTCACTTTTCTAAAGGAGCGTGGACTTTCTCTGATCAAGATCATGGATGGCAAGTTTCGGACTGTA
CAGCAGAAAGTTTGAAGTGTTGCCTAAATTTCTCAATGATGTCACCTGAAATCGTTGGCGAGAAAATCGAACCT
GAGAGGTTATATGATGCTGTCAATTTCATACTCTCTCTTCAGGACAAAACTACTGGTGGATTAGCAGTTTGGGA
AAAAGCCGGTGCCTCGTTGTTATTGGAGTGGCTCAATCCTGTCGAGTTTCTTGAGGACCTTATTGTCGAGCATA
CGTACGTCGAATGCACTGCTTCAGCAATTGAAGCATTTGTTTTGTTTAGGAAATTATACCCACATCACCGAAAG
AAGGAGATTGATAATTTCATTGTAAAAGCTGTACAGTATATTGAACACGAACAAACTGCCGATGGTTCATGGT
ATGGAAATTGGGGAATTTGCTTCCTATACGGTTCATGTTTTGCACTTGGAGGCTTGGCTGCTGCTGGCAAAACT
TACCACAATTGTGAAGCCATTCGTAGAGGAGTTGATTTTCTGCTAAAAGCACAAAGTGATGATGGTGGCTGGG
GAGAGAGCTACCAGTCATGCCCAAATAAGATATATACACCACTTGATGGGAAGAGATCAACTGTGGTACACAC
TGCATTGGCTGTCCTTGGTTTAATTCATGCCGGGCAGGCTGAGAGAGACGCCACTCCTATTCATCGCGGTGTCA
AGTTTTTGATCAACTCTCATTTGGAAAATGGAGACTTCCCACAACAGGAAATTATGGGAGTTTTCATGAGAAAC
TGCATGTTACATTATGCACTATACAAGAATACTTTTCCATTGTGGGCTTTAGCTGAATACCGAAGGAAAGTTCC
ATTGCCTAATTGAAAAGACAATTAAAAAAAATGTGCATAGAGATTTCGTGCATCATGGATTCATGCTGATCACT
TGCTCTACTACATTAATGTAATCGACGGAGCAAATGCTATGATGTTTTTTTATTTTATTTTTATAATAATAATAAA
AAATAAGTAATATTCCGGAATCAAAAAAAAAAAAAAAAAAAAAAAAAAAAAA

>AaOSC3_TRINITY_DN3249_c1_g2_i1 len=1786 path=[0:0-1785]

TGGGAAAAGATTTGTTGGTCCAATAACACCACTCATTTTACAATTGAGAGAAGAACTTTACACTCAGACATACA
ATGAGATAAATTGGAGCAAAACGCGCCATTTATGCGCAAAGGAGGATCTTTACTATCCCCATACTCTTATACAA
AAACTTTTATGGGATAGTCTTTATTACACGACAGAGCCCCTTCTTACGTGTTGGCCGTTTAAAAAATTGAGGGG
GAACAGTCTCCAAGTGACCATGCACCATATTCATTATGAAGATGAGGCCAGTCAATACATTACAATTGGGTGT
GTTGAAAAGCCGTTATTTATGCTTGCTTGTTGGATCGAAGATCCTAATGGAGATTGTTTCAAGAAGCATCTCGC
TAGGATTCATGATTACTTATGGCTTGGAGAAGATGGAATGAGAATCCATAGTTTTGGCAGTCAAACATGGGAT
TGTGTTTTTGCCATTCAATCTTTACTTGCCAGCAATCTCATCAATGAAATCGGAGCAATACTCATGAAAGGACAT
GAATTCATAAAGAATTCCCAGGTCTCCAATAATCCTCCGGGCGATTTTAGGAAAATGTTTCGCCACATCTCTAA
GGGTGGATGGACTTTCTCTGATCAAGACCATGGATGGAAAGTTTCTGATTGCACTGCAGAAGGCTTATACTGT
TGCCTATATATGTCAACGATGTCACCAGAAGTTGTTGGGGAGAAGATGGAACCTGAAAGGTTATATGATGCTG
TTAATATCCTTCTTTCAATGCAGAGTAAAAATGGTGGCGTATCAGCCTGGGAGCCAGCAGGAGCTCAGTCATG
GTTGGAGATGCTCAATCCGGTGGAATTCCTCTCGGATCTGATCATTGAATATGAGTATCCTGAGTGCACTGCGT
CAGCAATCAAAGCCCTCTCATTGTTTCAGAAGTTATGCCCAGAGCACAGGAAAAACGAGATTGAAAAGTTTTTA
AGCAAAGCTGTTAAATTTCTTGAAGACTCACAATTTCCAGATGGTTCATGGTATGGGCACTGGGGAATCTGCTT
CATATACGGTACGTGGTATGCTCTTAAAGGGCTAGCGGTTGCTGGCAAGACTTACAGTGATTGTTTGGCTATG
CGCAAGGGTGCTGATTTTCTACTCAAAACACAAGCACATGATGGCGGCTGGGGAGAGAGCCATCTTTCCTGCC
CAAACAAGAAATATATACCTCTTGAGGGAAATAGATCAAATTTGGTACAGACTTCGTGGGCTATGATGGGTTT
AATTCATTCTGGACAGATGGAAAGAGACCCTACTCCCCTTCACCGTGCTGCGAAGCTATTGATTAATTCTCAGC
TTGAGAACGGCGATTTTCCACAACAGGAACTCACAGCATCTTTTATGGTGAACTGTATGATACAGTTCGCAATG
TATAGAAGTACTTTTCCGTTATGGGCTTTGGCTGAATACCGCTCAAAGGTTCCATTCCCTTCCAGCAAATTTTAG
GGGATCCATTAGCACTATAAAAGGGTTGCCATAAAGGAGGGATGTGAAGATCTTTGTGTATTTATGTTTTATAA
ATTATTACAATTAAAAACAAAACGTATAATAGGTTACGGATCAAGAATAACTTAAATAAAATGCAATTTGAAAA
TTGTATTTAAAATATTCTGCAACCTCTGTTCATGATTCGTGAAGTAACAAAGATTTGTGCCTATACATATTATAG
TTTATCTCATAAATGATGAGCTAAACATGTGTCTATATGAAAATGTATACACATGCACACTCATAAAATACAAA
ATATAGTAACTTGCA

>AaOSC3_k69_1_Locus_9685_Transcript_1/1_Confidence_1.000_Length_2787_(reversed)_-_ORF_1_(frame_3) Locus_9685_Transcript_1/1_Confidence_1.000_Length_2787

TTGTATTTGAAAGAGAGAAAAGAAGTGAAAAAAATGTGGAGGCTTAAGATAGGAGAGGGTCGTGGGGATAA
TAACCCATACTTACGCAGCACAAACAACTTTGTTGGAAGGCAGACATGGGAGTTTGACCCAAACGCTGGCACT
CCTGAAGAGCGAGCTGAGGTTGAAGAGGCTCGCCAAAATTTCTACAGAAACCGTTTTCGAATCAGGCCTGGCT
CAGACCTCATCTGGCGTATGCAGTTTCTGAGAGAAAAAAAATTCATACAGAAAATTCCGCCACTAAAAGTTCAG
GATGGAGAAGAGATCACATATGAAGTTGCCACAGCCGCACTGAAGAGGGCAGTCCACTACCTTTCAGCTTTGC
AATCAAAGGATGGCCACTGGCCTGCTGATAATTCTGGCCCACTATTTTACCATCCTCCCTTTGTCATGTGTCTGT
ATATTACAGGATATCTTAGCATTTTATTCTCCGCTGAACACCGAAAAGAGATGCTTCGTTACATATACTATCATC
AGAATGAAGATGGTGGGTGGGGATTATACGTCGGGGGCCATAGCACAATGTTTTGCACTGCTTTCAACTATGT
TTGTATGCGTCTACTTGGAGAAGGACCTGATGGTGGTGAAAACAATGCTTGTGAAAAATCACGAAAATGGATT
TTGGATCATGGTGGTTTAACAGCTATACCTTCTTGGGGAAAGACTTGGCTTTCAATACTTGGAGTGTATGATTG
GTCCGGATGTAATCCAATGCCTCCAGAATTTTGGTCGTTTCCCTCTTTTCTTCCCATTCATCCAGCAAAAATGCTC
AACTACTGTCGGTTGACATATATGCCTATGTCATATTTATATGGGAAAAGATTTGTTGGTCCAATAACACCACTC
ATTTTACAATTGAGAGAAGAACTTTACACTCAGACATACAATGAGATAAATTGGAGCAAAACGCGCCATTTATG
CGCAAAGGAGGATCTTTACTATCCCCATACTCTTATACAAAAACTTTTATGGGATAGTCTTTATTACACGACAGA
GCCCCTTCTTACGTGTTGGCCGTTTAAAAAATTGAGGGGGAACAGTCTCCAAGTGACCATGCACCATATTCATT
ATGAAGATGAGGCCAGTCAATACATTACAATTGGGTGTGTTGAAAAGCCGTTATTTATGCTTGCTTGTTGGATC
GAAGATCCTAATGGAGATTGTTTCAAGAAGCATCTCGCTAGGATTCATGATTACTTATGGCTTGGAGAAGATG
GAATGAGAATCCATAGTTTTGGCAGTCAAACATGGGATTGTGTTTTTGCCATTCAATCTTTACTTGCCAGCAATC
TCATCAATGAAATCGGAGCAATACTCATGAAAGGACATGAATTCATAAAGAATTCCCAGGTCTCCAATAATCCT
CCGGGCGATTTTAGGAAAATGTTTCGCCACATCTCTAAGGGTGGATGGACTTTCTCTGATCAAGACCATGGAT
GGAAAGTTTCTGATTGCACTGCAGAAGGCTTATACTGTTGCCTATATATGTCAACGATGTCACCAGAAGTTGTT
GGGGAGAAGATGGAACCTGAAAGGTTATATGATGCTGTTAATATCCTTCTTTCAATGCAGAGTAAAAATGGTG
GCGTATCAGCCTGGGAGCCAGCAGGAGCTCAGTCATGGTTGGAGATGCTCAATCCGGTGGAATTCCTCTCGGA
TCTGATCATTGAATATGAGTATCCTGAGTGCACTGCGTCAGCAATCAAAGCCCTCTCATTGTTTCAGAAGTTAT
GCCCAGAGCACAGGAAAAACGAGATTGAAAAGTTTTTAAGCAAAGCTGTTAAATTTCTTGAAGACTCACAATT
TCCAGATGGTTCATGGTATGGGCACTGGGGAATCTGCTTCATATACGGTACGTGGTATGCTCTTAAAGGGCTA
GCGGTTGCTGGCAAGACTTACAGTGATTGTTTGGCTATGCGCAAGGGTGCTGATTTTCTACTCAAAACACAAG
CACATGATGGCGGCTGGGGAGAGAGCCATCTTTCCTGCCCAAACAAGAAATATATACCTCTTGAGGGAAATAG
ATCAAATTTGGTACAGACTTCGTGGGCTATGATGGGTTTAATTCATTCTGGACAGATGGAAAGAGACCCTACTC
CCCTTCACCGTGCTGCGAAGCTATTGATTAATTCTCAGCTTGAGAACGGCGATTTTCCACAACAGGAACTCACA
GCATCTTTTATGGTGAACTGTATGATACAGTTCGCAATGTATAGAAGTACTTTTCCGTTATGGGCTTTGGCTGA
ATACCGCTCAAAGGTTCCATTCCCTTCCAGCAAATTTTAG

>TRINITY_DN11420_c0_g1_i7 >TRINITY_DN11420_c0_g1_i7 len=2839 path=[0:0-95 5:96-206 6:207-212 7:213-277 9:278-301 11:302-373 12:374-401 13:402-451 15:452-609 17:610-649 18:650-759 19:760-794 21:795-820 22:821-844 25:845-897 31:898-1035 32:1036-1046 35:1047-1119 36:1120-1172 39:1173-1288 40:1289-1360 41:1361-1475 43:1476-1843 44:1844-1846 45:1847-2341 47:2342-2446 49:2447-2492 50:2493-2516 52:2517-2573 54:2574-2767 55:2768-2838]

TTTTTTTTAAATAATAATAATAATAATTAAAAGTAAAAAAGAAATTCGTACAAATTATTGTTTTTTGCCTGTTCTA
TTTTTGCACAAAATAATCTCTAACCCTTGAATGCATCCAAAGGACTGAGACTTTAGTTCCGCATGTTATATACAT
ACATACATACATATATATATATATGTATTGCATCTATCTGTTATTTGCATTTCAATCCAGGCTTATTTTGTTATTGC
ATCATCGGGAAATTGAAATTTACATCAACAAAAGCCGTTGACGATATTCCCCAAGAGCCCAAATGGGGAAGAT
GTTTCGATATGCTGAGTAATTTAGACTGCAAGTCCTCATAAAAACTCCTGTTATTTCCTGCTGTGGGAAGTCGCC
ATCTTCCATTTGTGAATTTATCAACACCCTTATTCCCCTGTGGATTGGTGTAGGATCTACTTGGCTCTGTCCTGCA
ACGATTAAAGATAACAAAGCCCAGGATGTTTGGACCAAATTTGCACGATCACCTTTTAGATTCGTATACACCTT
ATTTTGGCTGGAAAGATAACTCTCTCCCCATCCACCACAAGGTAGCTGCTTTGATAACAAAAATTCACAAGCTTT
GCGTATAGCCGGGCTATTGTGGTAGGTTTTCCCGCAAGCTGCCAGCCCCTCTACTGCAAACCATGTCCCATAGG
TGTAGCAAATTCCCCAGCAACCATACCAGGAACCATCAGGCTTTTGTGTATCTTCAATGTAATGAATTGCTCTTG
AGATGCAATGGTCTATTTCCTTGCGTCGATGCTGGGGATATAACTTCCTAAAAATTGCTAGAGCTTGGATTACT
GATGAGGTGCATTCAACATACTCTGTTTCAATAAGAGCATCTTCAAAGAACTCAATAGTATTGAACTTCTCCATC
CAACGGAATGCTCTTTGAGGCTCCCAAGCTGGGAAACCGCCGTTGCTACTTTGTAAAGAAAGAATGACATTTA
CAGCATCATAAAACCTCTCTGCTTCCATGCCTTCACCAACAAGGTCTGATGACATTTGTGACAACAATAGTGTG
GCCTTTAGTCCTTCTGCAGTGCAATCAGAGACTTGCCAACCGTGATCCTGGGTGGAGAATGTCCATGCTCCTTT
AGAAATGTGCCGATACATTGCAAAGAAATTGCCCGAAGGGTTTTCTCGGACCTGTGATGCTTTCGCAAAGTCA
TGTGCTTTGCGAAGTGTTGACCCATATTCTTCAGTTAGATTACTTGAGATAATGGCTTGAATGGCAAAAGCTGC
ATCCCACATTTGAGAACCAAAGGACTGAAATTTCAAGCCATCTTCGGCAACCCAATAGTAATCTGGAACTCTGG
CTAAATGACGCTTGTAAGCTACAGAATTTGGATCTTCGATCCACCATACAATCAAACATAGAACCTTTTCCATGC
ATCCAATGCAAAAATATCTACTATTTTTATCTTCATAACGAACATGTTCGATAGCAACTTTTAGAGCCTTTTCTCG
CATCATAGAAAAAGGCCAACGGGTAAGGATAGGCTCAGCAACATGGTGTAATAATCCCCATGTTAAATCTTGA
ATCAATGGCTGTGGATAATAGCGATCCTCCTTGGCAACTGTGTTTCGAGCTTTATTCCAATTGATACGATGATAT
GGTTCATTGTAAAGCTCTTGTCTAAGTGATAGAATCAATGGGGTGATTGGGCCGACAAATCTCTTTCCATATAA
ATATGACATAGCCATATAACCCAAGCGGCTATAGCAAAACATTTTGCCTGGATGAATTGGAAACATTTTTGGAA
GAAGCCATAGCTCCGGAGGCATAGGATTGCCACCGCACCACTCATAGATTCCAAGTACCGAAACCCAAAATTT
TCCCCAAGAAGGAATGCACACTAAAGCACCATGGTCAAGGATCCATTTTCGGGCTCTAGCAATGGCCACTTTGT
CCCCCTCTTCCCCAAGCAATCTCAAGGCAATGTAGCTTAGTGCTGAACCAAACATTGTGCTTTCCCCTTCTATGT
GTAGTCCCCAACCCCCATCTTCATTCTGATGATTGTATAAGTAGCGTTTAATTTCTTTCCTGTGTTCTGGTGATAG
CACAGCGTTAATTCCTCCAGTAACATACAATCCCACAACTAAAGGCTGAATGAAGAACATTGGCCCAGCAGATT
CAGCAGGCCAGTGACCATCATGTGCCTGAATGGAGGAGAAGAAGCTTATGGCTCTTCTAAGTGTAGTAGTCAC
TGATTCTTCTGTTATCTTCTCCGTTTCTTTCACTTTTACGGCTTTTGGAATTGGCCCTCTGGGATTTTCTTTAGTCA
GCTGCATTCTCATGAGGAGATCAGCACTTTGTTTGACTTGAAATCGATTCTTCTTAAAATCCTCTCGAGCTTTTTC
AACTTGAACACGCTCTTCAGGGGTTCCGGCATCGGGATCAAATTCCCAGTGCTGTCTTCCGACGTGATTATTAC
CTGTCGTGAGCCATGCACCTCCTTCTGCTATCTTTAGCTTCCACATGATCTCTCTCTAATATCGAACTCCAAAAGA
AAAAATTATATAAGTACCACGAGAGAGATTCACTTGTGTAATTCCTGGACTACGTTATATGGACGTAGGTTCTT
GAAGAAGAAGATATACAAAATAATAATAATAAAAAGAAGAATATTGTACACTAAAAGGAGAAGCTGGAGATT
CAGGTTATGATATAAAAGATGGACCAATGTGATTGAAATATATTTTGATGATAGTATTAATCAACAAAAGCAG
AATCCATATACCTTTGAAGATAAACGAGGCAAAGAACTTAGTTCTCAAAAGAGTGAAGCACAAAATGTTGAA
GACTGTTTCTG

>TRINITY_DN11420_c0_g1_i9 >TRINITY_DN11420_c0_g1_i9 len=2866 path=[0:0-95 4:96-233 6:234-239 7:240-304 9:305-328 11:329-400 12:401-428 13:429-478 15:479-636 17:637-676 18:677-786 19:787-821 21:822-847 22:848-871 25:872-924 31:925-1062 32:1063-1073 35:1074-1146 36:1147-1199 39:1200-1315 40:1316-1387 41:1388-1502 43:1503-1870 44:1871-1873 45:1874-2368 47:2369-2473 49:2474-2519 50:2520-2543 52:2544-2600 54:2601-2794 55:2795-2865]

TTTTTTTTAAATAATAATAATAATAATTAAAAGTAAAAAAGAAATTCGTACAAATTATTGTTTTTTGCCTGTTCTA
TTTTTGCACAAAATAATCTCAACCCTTGAATGCATCCAAAGGGCTGAGACTTTAGATCCGCATGTTAACAGTAA
TACAGAGAGGGGATATTAGTAAGCTTAATATATTTATGTATTGCATCTATCTGTATTTTATATTCAATCCACATG
CATAACTTCCCAGGCTTATTTTGTTATTGCATCATCGGGAAATTGAAATTTACATCAACAAAAGCCGTTGACGAT
ATTCCCCAAGAGCCCAAATGGGGAAGATGTTTCGATATGCTGAGTAATTTAGACTGCAAGTCCTCATAAAAACT
CCTGTTATTTCCTGCTGTGGGAAGTCGCCATCTTCCATTTGTGAATTTATCAACACCCTTATTCCCCTGTGGATTG
GTGTAGGATCTACTTGGCTCTGTCCTGCAACGATTAAAGATAACAAAGCCCAGGATGTTTGGACCAAATTTGCA
CGATCACCTTTTAGATTCGTATACACCTTATTTTGGCTGGAAAGATAACTCTCTCCCCATCCACCACAAGGTAGC
TGCTTTGATAACAAAAATTCACAAGCTTTGCGTATAGCCGGGCTATTGTGGTAGGTTTTCCCGCAAGCTGCCAG
CCCCTCTACTGCAAACCATGTCCCATAGGTGTAGCAAATTCCCCAGCAACCATACCAGGAACCATCAGGCTTTT
GTGTATCTTCAATGTAATGAATTGCTCTTGAGATGCAATGGTCTATTTCCTTGCGTCGATGCTGGGGATATAACT
TCCTAAAAATTGCTAGAGCTTGGATTACTGATGAGGTGCATTCAACATACTCTGTTTCAATAAGAGCATCTTCA
AAGAACTCAATAGTATTGAACTTCTCCATCCAACGGAATGCTCTTTGAGGCTCCCAAGCTGGGAAACCGCCGTT
GCTACTTTGTAAAGAAAGAATGACATTTACAGCATCATAAAACCTCTCTGCTTCCATGCCTTCACCAACAAGGTC
TGATGACATTTGTGACAACAATAGTGTGGCCTTTAGTCCTTCTGCAGTGCAATCAGAGACTTGCCAACCGTGAT
CCTGGGTGGAGAATGTCCATGCTCCTTTAGAAATGTGCCGATACATTGCAAAGAAATTGCCCGAAGGGTTTTCT
CGGACCTGTGATGCTTTCGCAAAGTCATGTGCTTTGCGAAGTGTTGACCCATATTCTTCAGTTAGATTACTTGA
GATAATGGCTTGAATGGCAAAAGCTGCATCCCACATTTGAGAACCAAAGGACTGAAATTTCAAGCCATCTTCG
GCAACCCAATAGTAATCTGGAACTCTGGCTAAATGACGCTTGTAAGCTACAGAATTTGGATCTTCGATCCACCA
TACAATCAAACATAGAACCTTTTCCATGCATCCAATGCAAAAATATCTACTATTTTTATCTTCATAACGAACATGT
TCGATAGCAACTTTTAGAGCCTTTTCTCGCATCATAGAAAAAGGCCAACGGGTAAGGATAGGCTCAGCAACAT
GGTGTAATAATCCCCATGTTAAATCTTGAATCAATGGCTGTGGATAATAGCGATCCTCCTTGGCAACTGTGTTT
CGAGCTTTATTCCAATTGATACGATGATATGGTTCATTGTAAAGCTCTTGTCTAAGTGATAGAATCAATGGGGT
GATTGGGCCGACAAATCTCTTTCCATATAAATATGACATAGCCATATAACCCAAGCGGCTATAGCAAAACATTT
TGCCTGGATGAATTGGAAACATTTTTGGAAGAAGCCATAGCTCCGGAGGCATAGGATTGCCACCGCACCACTC
ATAGATTCCAAGTACCGAAACCCAAAATTTTCCCCAAGAAGGAATGCACACTAAAGCACCATGGTCAAGGATC
CATTTTCGGGCTCTAGCAATGGCCACTTTGTCCCCCTCTTCCCCAAGCAATCTCAAGGCAATGTAGCTTAGTGCT
GAACCAAACATTGTGCTTTCCCCTTCTATGTGTAGTCCCCAACCCCCATCTTCATTCTGATGATTGTATAAGTAG
CGTTTAATTTCTTTCCTGTGTTCTGGTGATAGCACAGCGTTAATTCCTCCAGTAACATACAATCCCACAACTAAA
GGCTGAATGAAGAACATTGGCCCAGCAGATTCAGCAGGCCAGTGACCATCATGTGCCTGAATGGAGGAGAAG
AAGCTTATGGCTCTTCTAAGTGTAGTAGTCACTGATTCTTCTGTTATCTTCTCCGTTTCTTTCACTTTTACGGCTTT
TGGAATTGGCCCTCTGGGATTTTCTTTAGTCAGCTGCATTCTCATGAGGAGATCAGCACTTTGTTTGACTTGAAA
TCGATTCTTCTTAAAATCCTCTCGAGCTTTTTCAACTTGAACACGCTCTTCAGGGGTTCCGGCATCGGGATCAAA
TTCCCAGTGCTGTCTTCCGACGTGATTATTACCTGTCGTGAGCCATGCACCTCCTTCTGCTATCTTTAGCTTCCAC
ATGATCTCTCTCTAATATCGAACTCCAAAAGAAAAAATTATATAAGTACCACGAGAGAGATTCACTTGTGTAAT
TCCTGGACTACGTTATATGGACGTAGGTTCTTGAAGAAGAAGATATACAAAATAATAATAATAAAAAGAAGAA
TATTGTACACTAAAAGGAGAAGCTGGAGATTCAGGTTATGATATAAAAGATGGACCAATGTGATTGAAATATA
TTTTGATGATAGTATTAATCAACAAAAGCAGAATCCATATACCTTTGAAGATAAACGAGGCAAAGAAACTTAGT
TCTCAAAAGAGTGAAGCACAAAATGTTGAAGACTGTTTCTG

>TRINITY_DN2783_c0_g1_i1 >TRINITY_DN2783_c0_g1_i1 len=2997 path=[0:0-474 7:475-506 8:507-682 10:683-754 13:755-808 18:809-848 20:849-906 22:907-932 23:933-1164 30:1165-1180 32:1181-1279 33:1280-1403 34:1404-1523 35:1524-2552 36:2553-2996]

GTGGAATGCAAGGCCTTATATCTTGGAATCAAATATGGAGATTACCTGCATCAAATAAAGATCCTTCAACTTAA
TTACACACTATAAAGAAAGGCTAATGATTGTCAAGTTGTAAATAGTTGTCAATTGTAAAAAAAAAAAAAGGATA
ATCCTTATTTAGTTAGAATTTATTACTTATCAAAAAGACCTCCATCGGTTATAATACTAACGGAATAAATTTGTA
TTGCTTTCACATATATCATACACGATCCAATAATATTCAGTTCAAAACTTCTTTAGTCAAGACGTGCAAGAAACT
AGTGCAAATCAATAACAAAAATGAACCACTTGCATCGATCAATTCCTTTTATATATGTTGATCGAGCTGCATTGA
GAATATCTTCTTATTAATTTAAACAATTGTTTTAGAAGGCAATCGAACTTTTCTGTGGTATTCAGCAAGAGCCCA
CAATGGGTAAATATTTCGATAGGCTGCATAGTGTAACATGCAGTTCTTCATGAACACTCCTGTAATCTCCTGTTG
GGGAAAATCACCATCTTCAAGTTGAGAATTGATTATCATCTTAGCTGCACGGTGGAGAGGAGTTGGGTCTCTTT
CCCCCTGTCCAGCATGCATTAGACCCATCATGGCCCATGAAGTATGCACTAAATTCGACCTGTTTCCTTCAAGTG
GTATGTATTTCTATATGATCATCACCCAATATACAATGGTTGGTTAGTTGTTGGATATCAACTCGAGGGTTAAA
AATATTACCTTCTCAGGACAAGATCGGTAACTCTCTCCCCAACCGCCGTCGTCTCTCTGAGATTTAAGAAGAAA
ATCAACACCTTTGCGCATGGCTGCACAATTGTTGTGGTTTCTGCCAACAGCCGCCAGCCCTCCAAGTGCAAACC
AAGTACCATATGTAAAGCAAACTCCCCAATTTCCATACCATGAACCGTCAGGGAGTTGCACGTTTTCCAAGTAT
CGAACGGCATTTTCGATGAAACTGTCAATCTCTTTCTTCCTATGTCCTGGATATAGCATCTTGAATAAAACTAAG
GCATGGATTGCGGATGCGGTGCATTCAACATATTCATGCTCAATCACAATATCCGCAAAAAATTCCGTGGGATT
TAGAAGCTCCAACCATTCCTGAGCCCCGGCCGGCTCCCAAGCTGCTAATCCTCCATTTTTACTCTGTAATGAAAG
TAATACATTGACCGAATCGTATAACCGCTCAGGTTCCATTTTCTCACCCACAATTTGGGGCGGCATCATGGAGA
AAAGCAGGCAACACTTTAAACCTTCAGCGGTACAATCAGAAACTTGCCATCCATGATCTTGATCGGAAAATGTC
CAAGATCCCTTTGAAATATGACGATACATTGCCTTAAAGTCCCCGGAAGGATTGTCCTTGACCTGAGATGCCTT
GATGAATTCGTGTCCTCTCTTAAGTACAGTTCCAATTTCGTCAATGAGATTACTAGTCAGCAAAGCTTGAATAG
CAAAAGCAGCGTCCCACTCTTGGCTCCCAAAGCTCTGCATCTTCATTCCATCTTCAGCAATCCATAAATAATCCG
GAATTCTAGCAAGATGCTTTTTAAAATAATCTCCGTTTGGATCTTCGACCCAACAAGCAAGCATGCATAACACCT
TTTCCACACATCCAATTGTGATGTATCGACTGTTCTCATCTTCGTAATGAATGTGCTTCATTGTAACTTGAAGAG
CCTTTTCTCTGATCATCTTGTTGAATGGCCAACGAGTGAGAAATGGCTCTGTGAACACATATAAACTGTCCCAA
ATCAAGTCTTGTAGCAAAGGATGGGGATAGTAAAGATCCTCCTTTGCACATAAATGGCGTGCTTTCCTCCAATT
AACTGCATGATAAGGTTGAGCGTAGAGTTCTTCTCTCAATTGCAGAATGAGAGGTGTGATTGGTCCTACGAAC
TTCTTGCCATATAAATATGACATTGGCATGTATACCATGCGGCAATAGCACCACATTTTTGCTGGGTGGATGGG
AAGAAATGAAGGAAGGATCCAAAATTCCGGAGGCATTGGGTTGCTTCCGGACCAATCAAAAACACCGAGTAT
CGAAAGCCAAGTTTTTCCCCAGGAGGGAATGTGGGTAACACCACCACGGTCAAGAATCCATTTTCGAGCTCTT
GCACAAGCATTGTCTTGACCACCACCAGGTCCTTCTCCCAGAATTCGCATACAAATGTAGCTAAGTGCTGTGCA
AAACATGGAGCTGTGCCCCTCGATGTGCAATCCCCAACCACCATCTTCATTCTGATGATAGTAAATGTAACGAA
GTATTTCCTTGCGATGCTCGGCTGGGAACACAGTATCAACATGCCCAGTAATATACATGCACATTACTAAGGGA
GGAAGAAAAAATAAAGGACCGGCATTTTCAGCAGGCCAATGACCGTCGGTGGCCTGCAAAGCTGAGAAGAAA
TGGACGGCTCTCTTCAACGTTGTTGTCGCTGTTTCATATGTTATTTCTTCCCCATCTTCAACCTTTACTTGTGGGA
TTGTTTGTTTGAAATTTTTCTCCCTCAGAAACTGCATTCGCCAGAGGAGATCGGCACTGGGTTTGACGAGGTAA
CGGTTGTTGTAGAAGTTTTGACGAGCGGCTTCTACCTGAGCTCGCTCCTGAGGAGTGCCTGCCTCGGGATCAT
ACTCCCATATTTGCCTGCCCACGAAGTTGTTTGTGCTGTAAATGTAAGGGTCCTTGCCCCCATCTGCTACCTTAA
GCTTCCACATTAACTCCTTTCAGATGCTTTCCTTGTTCTTAGCGTTACACCTCTAATATGGCAGCTCAATGATAAA
TTGTTAGTTCGTCAAATCTTGGAGTAATTGAGGGAGCTAATAAGAGAAAACAACAAGGGCTTTTGCTAATATA
TATATCTTTGTGTGTGTGTATATGTGTACAGAAAGTCAGAAACGTATTCCAATTAAAGACCAGAAATTAACTTT
GGAATGGTGGCAGAAACTTTTATGATGATC

>TRINITY_DN2783_c0_g1_i12 >TRINITY_DN2783_c0_g1_i12 len=2925 path=[0:0-474 7:475-506 8:507-682 13:683-736 18:737-776 20:777-834 22:835-860 23:861-1092 30:1093-1108 32:1109-1207 33:1208-1331 34:1332-1451 35:1452-2480 36:2481-2924]

GTGGAATGCAAGGCCTTATATCTTGGAATCAAATATGGAGATTACCTGCATCAAATAAAGATCCTTCAACTTAA
TTACACACTATAAAGAAAGGCTAATGATTGTCAAGTTGTAAATAGTTGTCAATTGTAAAAAAAAAAAAGGATA
ATCCTTATTTAGTTAGAATTTATTACTTATCAAAAAGACCTCCATCGGTTATAATACTAACGGAATAAATTTGTA
TTGCTTTCACATATATCATACACGATCCAATAATATTCAGTTCAAAACTTCTTTAGTCAAGACGTGCAAGAAACT
AGTGCAAATCAATAACAAAAATGAACCACTTGCATCGATCAATTCCTTTTATATATGTTGATCGAGCTGCATTGA
GAATATCTTCTTATTAATTTAAACAATTGTTTTAGAAGGCAATCGAACTTTTCTGTGGTATTCAGCAAGAGCCCA
CAATGGGTAAATATTTCGATAGGCTGCATAGTGTAACATGCAGTTCTTCATGAACACTCCTGTAATCTCCTGTTG
GGGAAAATCACCATCTTCAAGTTGAGAATTGATTATCATCTTAGCTGCACGGTGGAGAGGAGTTGGGTCTCTTT
CCCCCTGTCCAGCATGCATTAGACCCATCATGGCCCATGAAGTATGCACTAAATTCGACCTGTTTCCTTCAAGTG
GTATGTATTTCTTCTCAGGACAAGATCGGTAACTCTCTCCCCAACCGCCGTCGTCTCTCTGAGATTTAAGAAGAA
AATCAACACCTTTGCGCATGGCTGCACAATTGTTGTGGTTTCTGCCAACAGCCGCCAGCCCTCCAAGTGCAAAC
CAAGTACCATATGTAAAGCAAACTCCCCAATTTCCATACCATGAACCGTCAGGGAGTTGCACGTTTTCCAAGTA
TCGAACGGCATTTTCGATGAAACTGTCAATCTCTTTCTTCCTATGTCCTGGATATAGCATCTTGAATAAAACTAA
GGCATGGATTGCGGATGCGGTGCATTCAACATATTCATGCTCAATCACAATATCCGCAAAAAATTCCGTGGGA
TTTAGAAGCTCCAACCATTCCTGAGCCCCGGCCGGCTCCCAAGCTGCTAATCCTCCATTTTTACTCTGTAATGAA
AGTAATACATTGACCGAATCGTATAACCGCTCAGGTTCCATTTTCTCACCCACAATTTGGGGCGGCATCATGGA
GAAAAGCAGGCAACACTTTAAACCTTCAGCGGTACAATCAGAAACTTGCCATCCATGATCTTGATCGGAAAAT
GTCCAAGATCCCTTTGAAATATGACGATACATTGCCTTAAAGTCCCCGGAAGGATTGTCCTTGACCTGAGATGC
CTTGATGAATTCGTGTCCTCTCTTAAGTACAGTTCCAATTTCGTCAATGAGATTACTAGTCAGCAAAGCTTGAAT
AGCAAAAGCAGCGTCCCACTCTTGGCTCCCAAAGCTCTGCATCTTCATTCCATCTTCAGCAATCCATAAATAATC
CGGAATTCTAGCAAGATGCTTTTTAAAATAATCTCCGTTTGGATCTTCGACCCAACAAGCAAGCATGCATAACA
CCTTTTCCACACATCCAATTGTGATGTATCGACTGTTCTCATCTTCGTAATGAATGTGCTTCATTGTAACTTGAAG
AGCCTTTTCTCTGATCATCTTGTTGAATGGCCAACGAGTGAGAAATGGCTCTGTGAACACATATAAACTGTCCC
AAATCAAGTCTTGTAGCAAAGGATGGGGATAGTAAAGATCCTCCTTTGCACATAAATGGCGTGCTTTCCTCCAA
TTAACTGCATGATAAGGTTGAGCGTAGAGTTCTTCTCTCAATTGCAGAATGAGAGGTGTGATTGGTCCTACGA
ACTTCTTGCCATATAAATATGACATTGGCATGTATACCATGCGGCAATAGCACCACATTTTTGCTGGGTGGATG
GGAAGAAATGAAGGAAGGATCCAAAATTCCGGAGGCATTGGGTTGCTTCCGGACCAATCAAAAACACCGAGT
ATCGAAAGCCAAGTTTTTCCCCAGGAGGGAATGTGGGTAACACCACCACGGTCAAGAATCCATTTTCGAGCTC
TTGCACAAGCATTGTCTTGACCACCACCAGGTCCTTCTCCCAGAATTCGCATACAAATGTAGCTAAGTGCTGTG
CAAAACATGGAGCTGTGCCCCTCGATGTGCAATCCCCAACCACCATCTTCATTCTGATGATAGTAAATGTAACG
AAGTATTTCCTTGCGATGCTCGGCTGGGAACACAGTATCAACATGCCCAGTAATATACATGCACATTACTAAGG
GAGGAAGAAAAAATAAAGGACCGGCATTTTCAGCAGGCCAATGACCGTCGGTGGCCTGCAAAGCTGAGAAG
AAATGGACGGCTCTCTTCAACGTTGTTGTCGCTGTTTCATATGTTATTTCTTCCCCATCTTCAACCTTTACTTGTG
GGATTGTTTGTTTGAAATTTTTCTCCCTCAGAAACTGCATTCGCCAGAGGAGATCGGCACTGGGTTTGACGAGG
TAACGGTTGTTGTAGAAGTTTTGACGAGCGGCTTCTACCTGAGCTCGCTCCTGAGGAGTGCCTGCCTCGGGAT
CATACTCCCATATTTGCCTGCCCACGAAGTTGTTTGTGCTGTAAATGTAAGGGTCCTTGCCCCCATCTGCTACCT
TAAGCTTCCACATTAACTCCTTTCAGATGCTTTCCTTGTTCTTAGCGTTACACCTCTAATATGGCAGCTCAATGAT
AAATTGTTAGTTCGTCAAATCTTGGAGTAATTGAGGGAGCTAATAAGAGAAAACAACAAGGGCTTTTGCTAAT
ATATATATCTTTGTGTGTGTGTATATGTGTACAGAAAGTCAGAAACGTATTCCAATTAAAGACCAGAAATTAAC
TTTGGAATGGTGGCAGAAACTTTTATGATGATC

>TRINITY_DN2783_c0_g1_i2 >TRINITY_DN2783_c0_g1_i2 len=2655 path=[1:0-184 4:185-198 5:199-199 6:200-324 7:325-356 9:357-482 12:483-537 15:538-566 17:567-586 18:587-626 19:627-654 21:655-684 22:685-710 24:711-722 26:723-768 27:769-908 28:909-942 30:943-958 32:959-1057 33:1058-1181 35:1182-2210 36:2211-2654]

TTTTTTTCAGCAGAAGGGAGAATATTTATTAAATTAAAGTTAAAGTACACTTAATTAAAAGGATGTAATGAGCT
TAATTTACCCCCTAAAAATAAGTAAAAAGAAAAGAAAAAAAAAGCACAAATGAACAATAAGTAAGAACTAAGC
GCATTGCTTTGATCGGCTCCTCTCTCTCTTTTTTTTTTATCAAAAAAAAAAAAGTTAATCGATCGAGCTATATTTA
ATTGGCTTCTTCTTGATTTAAACAGTAGTCTTGGAAGGCAATCGAACTCTCCTGCGGTATTCAGCAAGAGCCCA
TAAGGGGTAAATATTTCGATAGGCTGCGTAGTGTAACATGCAGTTCTTCATGAACACTCCAGTAATCTCCTGTT
GGGGAAAATCGCCATGTTCAAGTTGAGAATTGATTATCAACTTAGCTGCACGGTGGAGAGGAATTGGATCTCT
TTCCCCCTGTCCAGCATGTATTAGACCCATCATAGCCCACGCAGTATGCACCAAATTCGATCTATTTCCTTCAAG
TGGTACGTATTTCTTCTCAGGACAAGAACGATAGCTCTCTCCCCAGCCACCATCATCTCTCTGAGATGTAAGAA
GAAAATCAACACCTTTGCGCATGGCTGCACAATTATTGTAGCTTTTGCCAGCGGCTGTTAGTCCTCCAAGTGCA
AACCATGTACCATATGTGAAGCAAACTCCCCAATTTCCATACCAAGAACCATCAGGCTGTTGCACGTCTTCCAA
GTAGCGAACGGCATTGGCAATGAAACTATCAATCTCTTTCTTCCTGTGTCCGGGATATAGCTTCTTAAACAACA
CTAAAGAATGAATGGCGGATGAGGTGCACTCAACATATTCATGCTCAATTACAATATCCGCAAAAAATTCTGTG
GGATTGAGCAGTTCCAACCATTCCTGAGCTCCGGCCGGCTCCCACGCTGCCAAGCCTCCATTTTTACTCTGTAAT
GAAAGTAATACATTGACCGAATCGTATAACCGCTCAGGTTCCATTTTCTCACCCACAATTTGGGGCGGCATCAT
GGAGAAAAGCAGGCAACACTTTAAACCTTCAGCGGTACAATCAGAAACTTGCCATCCATGATCTTGATCGGAA
AATGTCCAAGATCCCTTTGAAATATGACGATACATTGCCTTAAAGTCCCCGGAAGGATTGTCCTTGACCTGCAT
CTTCATTCCATCTTCAGCAATCCATAAATAATCCGGAATTCTAGCAAGATGCTTTTTAAAATAATCTCCGTTTGG
ATCTTCGACCCAACAAGCAAGCATGCATAACACCTTTTCCACACATCCAATTGTGATGTATCGACTGTTCTCATC
TTCGTAATGAATGTGCTTCATTGTAACTTGAAGAGCCTTTTCTCTGATCATCTTGTTGAATGGCCAACGAGTGAG
AAATGGCTCTGTGAACACATATAAACTGTCCCAAATCAAGTCTTGTAGCAAAGGATGGGGATAGTAAAGATCC
TCCTTTGCACATAAATGGCGTGCTTTCCTCCAATTAACTGCATGATAAGGTTGAGCGTAGAGTTCTTCTCTCAAT
TGCAGAATGAGAGGTGTGATTGGTCCTACGAACTTCTTGCCATATAAATATGACATTGGCATGTATACCATGCG
GCAATAGCACCACATTTTTGCTGGGTGGATGGGAAGAAATGAAGGAAGGATCCAAAATTCCGGAGGCATTGG
GTTGCTTCCGGACCAATCAAAAACACCGAGTATCGAAAGCCAAGTTTTTCCCCAGGAGGGAATGTGGGTAACA
CCACCACGGTCAAGAATCCATTTTCGAGCTCTTGCACAAGCATTGTCTTGACCACCACCAGGTCCTTCTCCCAGA
ATTCGCATACAAATGTAGCTAAGTGCTGTGCAAAACATGGAGCTGTGCCCCTCGATGTGCAATCCCCAACCACC
ATCTTCATTCTGATGATAGTAAATGTAACGAAGTATTTCCTTGCGATGCTCGGCTGGGAACACAGTATCAACAT
GCCCAGTAATATACATGCACATTACTAAGGGAGGAAGAAAAAATAAAGGACCGGCATTTTCAGCAGGCCAAT
GACCGTCGGTGGCCTGCAAAGCTGAGAAGAAATGGACGGCTCTCTTCAACGTTGTTGTCGCTGTTTCATATGTT
ATTTCTTCCCCATCTTCAACCTTTACTTGTGGGATTGTTTGTTTGAAATTTTTCTCCCTCAGAAACTGCATTCGCC
AGAGGAGATCGGCACTGGGTTTGACGAGGTAACGGTTGTTGTAGAAGTTTTGACGAGCGGCTTCTACCTGAG
CTCGCTCCTGAGGAGTGCCTGCCTCGGGATCATACTCCCATATTTGCCTGCCCACGAAGTTGTTTGTGCTGTAA
ATGTAAGGGTCCTTGCCCCCATCTGCTACCTTAAGCTTCCACATTAACTCCTTTCAGATGCTTTCCTTGTTCTTAG
CGTTACACCTCTAATATGGCAGCTCAATGATAAATTGTTAGTTCGTCAAATCTTGGAGTAATTGAGGGAGCTAA
TAAGAGAAAACAACAAGGGCTTTTGCTAATATATATATCTTTGTGTGTGTGTATATGTGTACAGAAAGTCAGAA
ACGTATTCCAATTAAAGACCAGAAATTAACTTTGGAATGGTGGCAGAAACTTTTATGATGATC

>TRINITY_DN3249_c0_g1_i1 >TRINITY_DN3249_c0_g1_i1 len=2640 path=[0:0-802 2:803-837 3:838-881 4:882-908 6:909-1001 8:1002-1053 9:1054-1620 11:1621-1794 12:1795-1838 13:1839-1881 16:1882-1942 20:1943-1968 21:1969-2021 22:2022-2027 25:2028-2100 29:2101-2157 32:2158-2160 33:2161-2252 34:2253-2332 36:2333-2408 38:2409-2420 40:2421-2639]

ATAAATATGATGTTCTATTTTTCTCCTTGAACATACTGCATCGTTTCAAAAAAATCTCTAGCTTTTGCAGCCAACG
ACATCACCAACTCCAACACCAACCCCCTCTTCTTTCCTTGCTCTTGCCAACTGATTCTAAGTCAAGGGGGACGAT
AATATTATTATGTGGAGGCTTAAGATTGCAGAGGGTGACAAAAATAGCCCATACATTTTTACAACAAACAATTT
TGTGGGAAGGCAAATATGGGAATTTGATCCAAATTATGCTGCCTCGCCGGAAGAGCTAGCTGAAGTTGAAGA
GGCTCGCCAGAAGTTTCACAAAAATCGCCACAAGGTCAAGCCTGCCAGTGATCTTATGTGGCGGCTACAGTTC
CTTAGAGAGAAAAACTTCAAGCAAACAATCCCTCCAGTAAAGGTTAAGGATGAGGAGGAAATCACGTATGAA
ACGGCAACCAAGGCAGTGAAGAGGGCTGCTAGCTATTTTTCAGCCATACAGGCTAACGATGGCCACTGGCCTG
CTGAAAATGCTGGCCCTATGTACTTCCTTCCTCCATTTGTCTTCTGCCTGTACATTACAGGGCATCTTGATGCTGT
ATTTACAGCTGAGCACAAAAAAGAAATCCTTCGCTATTTGTACAATCATCAGCATGAAGATGGTGGATGGGGA
ATACACATAGAAGGCCACAGCAGCATGTTTGGCACAGTTTACGGCTACATTACTATGCGTTTACTTGGATTAGG
ACCCAATGATGGTGAAAACAATGCTTGTGCAAGAGCACGAAAATGGATTCGCGACAATGGTGGTGTCACATAC
ATACCCTCCTGGGGAAAGAATTGGCTGTCGATACTTGGATTGTTTGAATGGGCTGGAACCCACCCAATGCCGC
CTGAGTTCTGGCTGCTCCCTTCTTATTTTCCATTGCATCCAGCACAAATGTGGTGCTATTGCCGACTTGTTTACAT
GCCATTATCTTATTTGTATGGGAAAAGATTTGTTGGTCCAATCACTCCGCTTATTCAACAATTGAGGAATGAACT
TCACACTCAGCCGTACAAGGAAATAAATTGGAGGAAAGTTCGTCATTTATGTGCAAAGCCGGATCTCTACTATC
CCCACACCGTGGTACAGAACATACTTTGGGATGGTATGTACATGGCAACAGAGCCTCTCCTAACTCGTTGGCCT
TTGAACAAGTATCTTAGACAGAAGGCTTTAAAAGAAACAATGAAGATCATTCATTATGAAGACCAAAGTAGTA
GATACATTACCATAGGAAGTGTAGAAAAGCCTTTATGTATGCTTGCTTGTTGGGTTGAAGATCCCGATGGTGTT
GCCTTTAAGAAGCATCTTGCTAGAGTTTCAGATTACTTCTGGCTTGGAGAAGATGGAATGAAAGCTCAGACTTT
TGGAAGTCAAACATGGGATACTGCTCTTGGCCTTCAAGCTTTGCTTGCTTGCGATCTCGTCGATGAAATTGCAC
CTACTCTTGCAAAAGGACACGACTACTTAAAGAAAGCTCAGGTGAGGGATAATCCAATAGGCGATTATACAAG
CAATTTCCGTCACTTTTCTAAAGGAGCGTGGACTTTCTCTGATCAAGATCATGGATGGCAAGTTTCGGACAGTA
CAGCAGAGAGTTTGAAGTGTGTCCTACATTTCTCAATGATGCCACCTGAAATCGTTGGCGAAAAAATCGAGCC
TAAGAAGTTATACGATACTGTCAATTTCCTACTCTCTCTTCAGGATAAAACTACTGGCGGATTAGCAATTTGGG
AGAAAGCCGGTGCCTCATTGTTGTTGGAGTGGCTCAATCCTGTCGAGTTTCTTGAGGACCTTATTGTTGAGCAT
ACGTACGTTGAGTGCACTGCTTCAGCAATTGAGGCATTTGTTTTGTTTAAGAAATTATACCCACATCACCAAAA
GAAGGAGATTGATTATTTCCTCGTTAAAGCTGTACAGTATATTGAACATGAACAAACTGCCGATGGTTCATGGT
ATGGAAATTGGGGAATTTGCTTCCTATATGGTTCGTGTTTTGCTCTTGGAGGTTTGGCTGCTGCCGGCAAAACT
TACCGCAATTGCGAAGCCATTTGTAAAGGAATTGATTTTCTGTTAAAAGCACAAAGTGGTGATGGCGGTTGGG
GAGAGAGCTACCGATCGTGCCCAAATAAGATATATACACCACTTGATGGGAAGAGATCAACTGTGGTACACAC
TGCATTGGCTGTCCTTGGTTTAATTCATGCCGGGCAGGCAGAGAGAGAGACCCAACACCTATTCATCGTGGTGTA
AAGTATTTGATCAACTCTCAATTGGAAAAGGGAGACTTCCCGCAAGAGGAAATTATGGGAGTTTTCATGAGAA
ACTGCATGTTACATTATGCACTATACAAGAATACTTTTCCATTGTGGGCTTTAGCTGAATACCGAAGGAAAGTT
CCATTGCCTAATTGAAAAGACAATTAAAAAAAATGTGCATAGAGATTTCGTGCATCATGGATTCATGCTGATCA
CTTGCTCTACTACATTAATGTAATCGACGGAGCAAATGCTATGATGTTTTTTTATTTTATTTTTATAATAATAATA
AAAAATAAGTAATATTCCGGAATCAAAAAAAAAAAAAAAAAAAAAAAAAAAAAAA

>TRINITY_DN3249_c0_g1_i10 >TRINITY_DN3249_c0_g1_i10 len=2734 path=[0:0-802 2:803-837 3:838-881 4:882-908 6:909-1001 8:1002-1053 9:1054-1620 11:1621-1794 12:1795-1838 13:1839-1881 16:1882-1942 20:1943-1968 21:1969-2021 22:2022-2027 25:2028-2100 29:2101-2157 32:2158-2160 33:2161-2252 34:2253-2332 36:2333-2408 37:2409-2525 41:2526-2733]

ATAAATATGATGTTCTATTTTTCTCCTTGAACATACTGCATCGTTTCAAAAAAATCTCTAGCTTTTGCAGCCAACG
ACATCACCAACTCCAACACCAACCCCCTCTTCTTTCCTTGCTCTTGCCAACTGATTCTAAGTCAAGGGGGACGAT
AATATTATTATGTGGAGGCTTAAGATTGCAGAGGGTGACAAAAATAGCCCATACATTTTTACAACAAACAATTT
TGTGGGAAGGCAAATATGGGAATTTGATCCAAATTATGCTGCCTCGCCGGAAGAGCTAGCTGAAGTTGAAGA
GGCTCGCCAGAAGTTTCACAAAAATCGCCACAAGGTCAAGCCTGCCAGTGATCTTATGTGGCGGCTACAGTTC
CTTAGAGAGAAAAACTTCAAGCAAACAATCCCTCCAGTAAAGGTTAAGGATGAGGAGGAAATCACGTATGAA
ACGGCAACCAAGGCAGTGAAGAGGGCTGCTAGCTATTTTTCAGCCATACAGGCTAACGATGGCCACTGGCCTG
CTGAAAATGCTGGCCCTATGTACTTCCTTCCTCCATTTGTCTTCTGCCTGTACATTACAGGGCATCTTGATGCTGT
ATTTACAGCTGAGCACAAAAAAGAAATCCTTCGCTATTTGTACAATCATCAGCATGAAGATGGTGGATGGGGA
ATACACATAGAAGGCCACAGCAGCATGTTTGGCACAGTTTACGGCTACATTACTATGCGTTTACTTGGATTAGG
ACCCAATGATGGTGAAAACAATGCTTGTGCAAGAGCACGAAAATGGATTCGCGACAATGGTGGTGTCACATAC
ATACCCTCCTGGGGAAAGAATTGGCTGTCGATACTTGGATTGTTTGAATGGGCTGGAACCCACCCAATGCCGC
CTGAGTTCTGGCTGCTCCCTTCTTATTTTCCATTGCATCCAGCACAAATGTGGTGCTATTGCCGACTTGTTTACAT
GCCATTATCTTATTTGTATGGGAAAAGATTTGTTGGTCCAATCACTCCGCTTATTCAACAATTGAGGAATGAACT
TCACACTCAGCCGTACAAGGAAATAAATTGGAGGAAAGTTCGTCATTTATGTGCAAAGCCGGATCTCTACTATC
CCCACACCGTGGTACAGAACATACTTTGGGATGGTATGTACATGGCAACAGAGCCTCTCCTAACTCGTTGGCCT
TTGAACAAGTATCTTAGACAGAAGGCTTTAAAAGAAACAATGAAGATCATTCATTATGAAGACCAAAGTAGTA
GATACATTACCATAGGAAGTGTAGAAAAGCCTTTATGTATGCTTGCTTGTTGGGTTGAAGATCCCGATGGTGTT
GCCTTTAAGAAGCATCTTGCTAGAGTTTCAGATTACTTCTGGCTTGGAGAAGATGGAATGAAAGCTCAGACTTT
TGGAAGTCAAACATGGGATACTGCTCTTGGCCTTCAAGCTTTGCTTGCTTGCGATCTCGTCGATGAAATTGCAC
CTACTCTTGCAAAAGGACACGACTACTTAAAGAAAGCTCAGGTGAGGGATAATCCAATAGGCGATTATACAAG
CAATTTCCGTCACTTTTCTAAAGGAGCGTGGACTTTCTCTGATCAAGATCATGGATGGCAAGTTTCGGACAGTA
CAGCAGAGAGTTTGAAGTGTGTCCTACATTTCTCAATGATGCCACCTGAAATCGTTGGCGAAAAAATCGAGCC
TAAGAAGTTATACGATACTGTCAATTTCCTACTCTCTCTTCAGGATAAAACTACTGGCGGATTAGCAATTTGGG
AGAAAGCCGGTGCCTCATTGTTGTTGGAGTGGCTCAATCCTGTCGAGTTTCTTGAGGACCTTATTGTTGAGCAT
ACGTACGTTGAGTGCACTGCTTCAGCAATTGAGGCATTTGTTTTGTTTAAGAAATTATACCCACATCACCAAAA
GAAGGAGATTGATTATTTCCTCGTTAAAGCTGTACAGTATATTGAACATGAACAAACTGCCGATGGTTCATGGT
ATGGAAATTGGGGAATTTGCTTCCTATATGGTTCGTGTTTTGCTCTTGGAGGTTTGGCTGCTGCCGGCAAAACT
TACCGCAATTGCGAAGCCATTTGTAAAGGAATTGATTTTCTGTTAAAAGCACAAAGTGGTGATGGCGGTTGGG
GAGAGAGCTACCGATCGTGCCCAAATAAGATATATACACCACTTGATGGGAAGAGATCAACTGTGGTACACAC
TGCATTGGCTGTCCTTGGTTTAATTCATGCCGGGCAGGCAGAGAGAGACCCAACACCTATTCATCGTGGTGTA
AAGTATTTGATCAACTCTCAATTGGAAAAGGGAGACTTCCCGCAAGAGGAAATTATGGGAGTTTTCATGAGAA
ACTGCATGTTACATTATGCACTATACAAGAATACTTTTCCATTGTGGGCATTAGCTGAATATCGAAATAAAGTTC
GATTTCCTAATTGAAAGATCTATTCACTCTTTAGATGTATAAGAAGACGATAAATAATTGTGCATAGCACTTCGT
GCATGTCTCCATGCTTATCAATGGATTTACAACATTGTAATTGACGGAGCAAAAGGCGAAGATGATAATTTACA
GTAATAATGATAATAATATTCTTGAATCAAGTATTTGTTGCAACTTATCACATCTTTCTCTCAAGCTTACGTTTGC
GCAAGATTTAGCTCATGTTTGATATTATGTTGAATTTTCAAATAATTAATTTTAGTCTAGATTTTGAATTAAAA

>TRINITY_DN3249_c0_g1_i12 >TRINITY_DN3249_c0_g1_i12 len=2253 path=[0:0-802 1:803-837 3:838-881 5:882-908 6:909-1001 7:1002-1053 9:1054-1620 10:1621-1794 12:1795-1838 13:1839-1881 16:1882-1942 20:1943-1968 21:1969-2021 22:2022-2027 25:2028-2100 29:2101-2157 32:2158-2160 33:2161-2252]

ATAAATATGATGTTCTATTTTTCTCCTTGAACATACTGCATCGTTTCAAAAAAATCTCTAGCTTTTGCAGCCAACG
ACATCACCAACTCCAACACCAACCCCCTCTTCTTTCCTTGCTCTTGCCAACTGATTCTAAGTCAAGGGGGACGAT
AATATTATTATGTGGAGGCTTAAGATTGCAGAGGGTGACAAAAATAGCCCATACATTTTTACAACAAACAATTT
TGTGGGAAGGCAAATATGGGAATTTGATCCAAATTATGCTGCCTCGCCGGAAGAGCTAGCTGAAGTTGAAGA
GGCTCGCCAGAAGTTTCACAAAAATCGCCACAAGGTCAAGCCTGCCAGTGATCTTATGTGGCGGCTACAGTTC
CTTAGAGAGAAAAACTTCAAGCAAACAATCCCTCCAGTAAAGGTTAAGGATGAGGAGGAAATCACGTATGAA
ACGGCAACCAAGGCAGTGAAGAGGGCTGCTAGCTATTTTTCAGCCATACAGGCTAACGATGGCCACTGGCCTG
CTGAAAATGCTGGCCCTATGTACTTCCTTCCTCCATTTGTCTTCTGCCTGTACATTACAGGGCATCTTGATGCTGT
ATTTACAGCTGAGCACAAAAAAGAAATCCTTCGCTATTTGTACAATCATCAGCATGAAGATGGTGGATGGGGA
ATACACATAGAAGGCCACAGCAGCATGTTTGGCACAGTTTACGGCTACATTACTATGCGTTTACTTGGATTAGG
ACCCAATGATGGTGAAAACAATGCTTGTGCAAGAGCACGAAAATGGATTCGCGACAATGGTGGTGCCACATA
CATACCCTCTTGGGGAAAGAATTGGCTCTCGATACTTGGATTGTTTGAATGGGCTGGAACCCACCCAATGCCCC
CTGAATTCTGGTTGCTCCCTTCTTGTTTTCCATTGCATCCAGCACAAATGTGGTGCTATTGCCGACTTGTTTACAT
GCCATTATCTTATTTGTATGGGAAAAGATTTGTTGGTCCAATTACTCCACTTATTCAACAATTGAGGAAAGAACT
TCACACTCAGCCTTACAATGAAATAAATTGGAGGAAAGTTCGTCATTTATGTGCAAAGCCGGATCTCTACTATC
CCCACACCGTGGTACAGAACATACTTTGGGATGGTATGTACATGGCAACAGAGCCTCTCCTAACTCGTTGGCCT
TTGAACAAGTATCTTAGACAGAAGGCTTTAAAAGAAACAATGAAGATCATTCATTATGAAGACCAAAGTAGTA
GATACATTACCATAGGAAGTGTAGAAAAGCCTTTATGTATGCTTGCTTGTTGGGTTGAAGATCCCGATGGTGTT
GCCTTTAAGAAGCATCTTGCTAGAGTTTCAGATTACTTCTGGCTTGGAGAAGATGGAATGAAAGCTCAGACTTT
TGGAAGTCAAACATGGGATACTGCTCTTGGCCTTCAAGCTTTGCTTGCTTGCGATCTCGTCGATGAAATTGCAC
CTACTCTTGCAAAAGGACACGACTACTTAAAGAAAGCTCAGGTGAGGGATAATCCAATAGGCGATTATACAAG
CAATTTCCGTCACTTTTCTAAAGGAGCGTGGACTTTCTCTGATCAAGATCATGGATGGCAAGTTTCGGACTGTA
CAGCAGAAAGTTTGAAGTGTTGCCTAAATTTCTCAATGATGTCACCTGAAATCGTTGGCGAGAAAATCGAACCT
GAGAGGTTATATGATGCTGTCAATTTCATACTCTCTCTTCAGGACAAAACTACTGGTGGATTAGCAGTTTGGGA
AAAAGCCGGTGCCTCGTTGTTATTGGAGTGGCTCAATCCTGTCGAGTTTCTTGAGGACCTTATTGTTGAGCATA
CGTACGTTGAGTGCACTGCTTCAGCAATTGAGGCATTTGTTTTGTTTAAGAAATTATACCCACATCACCAAAAG
AAGGAGATTGATTATTTCCTCGTTAAAGCTGTACAGTATATTGAACATGAACAAACTGCCGATGGTTCATGGTA
TGGAAATTGGGGAATTTGCTTCCTATATGGTTCGTGTTTTGCTCTTGGAGGTTTGGCTGCTGCCGGCAAAACTT
ACCGCAATTGCGAAGCCATTTGTAAAGGAATTGATTTTCTGTTAAAAGCACAAAGTGGTGATGGCGGTTGGGG
AGAGAGCTACCGATCGTGCCCAAATAAGATATATACACCACTTGATGGGAAGAGATCAACTGTGGTACACACT
GCATTGGCTGTCCTTGGTTTAATTCATGCCGGGCAGGC

>TRINITY_DN3249_c0_g1_i15 >TRINITY_DN3249_c0_g1_i15 len=2734 path=[0:0-802 2:803-837 3:838-881 5:882-908 6:909-1001 7:1002-1053 9:1054-1620 11:1621-1794 12:1795-1838 13:1839-1881 15:1882-1899 17:1900-1938 19:1939-1942 20:1943-1968 21:1969-2021 22:2022-2027 24:2028-2046 26:2047-2078 27:2079-2100 29:2101-2157 32:2158-2160 33:2161-2252 34:2253-2332 36:2333-2408 37:2409-2525 41:2526-2733]

ATAAATATGATGTTCTATTTTTCTCCTTGAACATACTGCATCGTTTCAAAAAAATCTCTAGCTTTTGCAGCCAACG
ACATCACCAACTCCAACACCAACCCCCTCTTCTTTCCTTGCTCTTGCCAACTGATTCTAAGTCAAGGGGGACGAT
AATATTATTATGTGGAGGCTTAAGATTGCAGAGGGTGACAAAAATAGCCCATACATTTTTACAACAAACAATTT
TGTGGGAAGGCAAATATGGGAATTTGATCCAAATTATGCTGCCTCGCCGGAAGAGCTAGCTGAAGTTGAAGA
GGCTCGCCAGAAGTTTCACAAAAATCGCCACAAGGTCAAGCCTGCCAGTGATCTTATGTGGCGGCTACAGTTC
CTTAGAGAGAAAAACTTCAAGCAAACAATCCCTCCAGTAAAGGTTAAGGATGAGGAGGAAATCACGTATGAA
ACGGCAACCAAGGCAGTGAAGAGGGCTGCTAGCTATTTTTCAGCCATACAGGCTAACGATGGCCACTGGCCTG
CTGAAAATGCTGGCCCTATGTACTTCCTTCCTCCATTTGTCTTCTGCCTGTACATTACAGGGCATCTTGATGCTGT
ATTTACAGCTGAGCACAAAAAAGAAATCCTTCGCTATTTGTACAATCATCAGCATGAAGATGGTGGATGGGGA
ATACACATAGAAGGCCACAGCAGCATGTTTGGCACAGTTTACGGCTACATTACTATGCGTTTACTTGGATTAGG
ACCCAATGATGGTGAAAACAATGCTTGTGCAAGAGCACGAAAATGGATTCGCGACAATGGTGGTGTCACATAC
ATACCCTCCTGGGGAAAGAATTGGCTGTCGATACTTGGATTGTTTGAATGGGCTGGAACCCACCCAATGCCCC
CTGAATTCTGGTTGCTCCCTTCTTGTTTTCCATTGCATCCAGCACAAATGTGGTGCTATTGCCGACTTGTTTACAT
GCCATTATCTTATTTGTATGGGAAAAGATTTGTTGGTCCAATTACTCCACTTATTCAACAATTGAGGAAAGAACT
TCACACTCAGCCTTACAATGAAATAAATTGGAGGAAAGTTCGTCATTTATGTGCAAAGCCGGATCTCTACTATC
CCCACACCGTGGTACAGAACATACTTTGGGATGGTATGTACATGGCAACAGAGCCTCTCCTAACTCGTTGGCCT
TTGAACAAGTATCTTAGACAGAAGGCTTTAAAAGAAACAATGAAGATCATTCATTATGAAGACCAAAGTAGTA
GATACATTACCATAGGAAGTGTAGAAAAGCCTTTATGTATGCTTGCTTGTTGGGTTGAAGATCCCGATGGTGTT
GCCTTTAAGAAGCATCTTGCTAGAGTTTCAGATTACTTCTGGCTTGGAGAAGATGGAATGAAAGCTCAGACTTT
TGGAAGTCAAACATGGGATACTGCTCTTGGCCTTCAAGCTTTGCTTGCTTGCGATCTCGTCGATGAAATTGCAC
CTACTCTTGCAAAAGGACACGACTACTTAAAGAAAGCTCAGGTGAGGGATAATCCAATAGGCGATTATACAAG
CAATTTCCGTCACTTTTCTAAAGGAGCGTGGACTTTCTCTGATCAAGATCATGGATGGCAAGTTTCGGACAGTA
CAGCAGAGAGTTTGAAGTGTGTCCTACATTTCTCAATGATGCCACCTGAAATCGTTGGCGAAAAAATCGAGCC
TAAGAAGTTATACGATACTGTCAATTTCCTACTCTCTCTTCAGGATAAAACTACTGGCGGATTAGCAATTTGGG
AGAAAGCCGGTGCCTCATTGTTGTTGGAGTGGCTCAATCCTGTCGAGTTTCTTGAGGACCTTATTGTTGAGCAT
ACGTACGTTGAGTGCACTGCTTCAGCAATTGAGGCACTTATTTTCTTTAAGAAGTTATACCCACATCACCGAAA
GAAGGAGATTGATAATTTCCTCATTAAAGCTGTACAGTATATTGAACATGAACAAACTGCCGATGGTTCATGGT
ATGGAAATTGGGGAATTTGCTTCCTATATGGTTCATGTTTTGCTCTAGGAGGTTTGGCTGCTGCTGGCAAAACT
TACCACAATTGCGAAGCAGTTCGTAAAGGAGTCGATTTTCTGTTAAAAGCACAAAGTGGTGATGGCGGTTGGG
GAGAGAGCTACCGATCGTGCCCAAATAAGATATATACACCACTTGATGGGAAGAGATCAACTGTGGTACACAC
TGCATTGGCTGTCCTTGGTTTAATTCATGCCGGGCAGGCAGAGAGAGACCCAACACCTATTCATCGTGGTGTA
AAGTATTTGATCAACTCTCAATTGGAAAAGGGAGACTTCCCGCAAGAGGAAATTATGGGAGTTTTCATGAGAA
ACTGCATGTTACATTATGCACTATACAAGAATACTTTTCCATTGTGGGCATTAGCTGAATATCGAAATAAAGTTC
GATTTCCTAATTGAAAGATCTATTCACTCTTTAGATGTATAAGAAGACGATAAATAATTGTGCATAGCACTTCGT
GCATGTCTCCATGCTTATCAATGGATTTACAACATTGTAATTGACGGAGCAAAAGGCGAAGATGATAATTTACA
GTAATAATGATAATAATATTCTTGAATCAAGTATTTGTTGCAACTTATCACATCTTTCTCTCAAGCTTACGTTTGC
GCAAGATTTAGCTCATGTTTGATATTATGTTGAATTTTCAAATAATTAATTTTAGTCTAGATTTTGAATTAAAA

>TRINITY_DN3249_c0_g1_i2_ >TRINITY_DN3249_c0_g1_i2 len=2640 path=[0:0-802 2:803-837 3:838-881 5:882-908 6:909-1001 7:1002-1053 9:1054-1620 10:1621-1794 12:1795-1838 14:1839-1899 17:1900-1938 18:1939-1968 21:1969-2021 23:2022-2046 26:2047-2078 28:2079-2150 31:2151-2157 32:2158-2160 33:2161-2252 35:2253-2332 36:2333-2408 38:2409-2420 40:2421-2639]

ATAAATATGATGTTCTATTTTTCTCCTTGAACATACTGCATCGTTTCAAAAAAATCTCTAGCTTTTGCAGCCAACG
ACATCACCAACTCCAACACCAACCCCCTCTTCTTTCCTTGCTCTTGCCAACTGATTCTAAGTCAAGGGGGACGAT
AATATTATTATGTGGAGGCTTAAGATTGCAGAGGGTGACAAAAATAGCCCATACATTTTTACAACAAACAATTT
TGTGGGAAGGCAAATATGGGAATTTGATCCAAATTATGCTGCCTCGCCGGAAGAGCTAGCTGAAGTTGAAGA
GGCTCGCCAGAAGTTTCACAAAAATCGCCACAAGGTCAAGCCTGCCAGTGATCTTATGTGGCGGCTACAGTTC
CTTAGAGAGAAAAACTTCAAGCAAACAATCCCTCCAGTAAAGGTTAAGGATGAGGAGGAAATCACGTATGAA
ACGGCAACCAAGGCAGTGAAGAGGGCTGCTAGCTATTTTTCAGCCATACAGGCTAACGATGGCCACTGGCCTG
CTGAAAATGCTGGCCCTATGTACTTCCTTCCTCCATTTGTCTTCTGCCTGTACATTACAGGGCATCTTGATGCTGT
ATTTACAGCTGAGCACAAAAAAGAAATCCTTCGCTATTTGTACAATCATCAGCATGAAGATGGTGGATGGGGA
ATACACATAGAAGGCCACAGCAGCATGTTTGGCACAGTTTACGGCTACATTACTATGCGTTTACTTGGATTAGG
ACCCAATGATGGTGAAAACAATGCTTGTGCAAGAGCACGAAAATGGATTCGCGACAATGGTGGTGTCACATAC
ATACCCTCCTGGGGAAAGAATTGGCTGTCGATACTTGGATTGTTTGAATGGGCTGGAACCCACCCAATGCCCC
CTGAATTCTGGTTGCTCCCTTCTTGTTTTCCATTGCATCCAGCACAAATGTGGTGCTATTGCCGACTTGTTTACAT
GCCATTATCTTATTTGTATGGGAAAAGATTTGTTGGTCCAATTACTCCACTTATTCAACAATTGAGGAAAGAACT
TCACACTCAGCCTTACAATGAAATAAATTGGAGGAAAGTTCGTCATTTATGTGCAAAGCCGGATCTCTACTATC
CCCACACCGTGGTACAGAACATACTTTGGGATGGTATGTACATGGCAACAGAGCCTCTCCTAACTCGTTGGCCT
TTGAACAAGTATCTTAGACAGAAGGCTTTAAAAGAAACAATGAAGATCATTCATTATGAAGACCAAAGTAGTA
GATACATTACCATAGGAAGTGTAGAAAAGCCTTTATGTATGCTTGCTTGTTGGGTTGAAGATCCCGATGGTGTT
GCCTTTAAGAAGCATCTTGCTAGAGTTTCAGATTACTTCTGGCTTGGAGAAGATGGAATGAAAGCTCAGACTTT
TGGAAGTCAAACATGGGATACTGCTCTTGGCCTTCAAGCTTTGCTTGCTTGCGATCTCGTCGATGAAATTGCAC
CTACTCTTGCAAAAGGACACGACTACTTAAAGAAAGCTCAGGTGAGGGATAATCCAATAGGCGATTATACAAG
CAATTTCCGTCACTTTTCTAAAGGAGCGTGGACTTTCTCTGATCAAGATCATGGATGGCAAGTTTCGGACTGTA
CAGCAGAAAGTTTGAAGTGTTGCCTAAATTTCTCAATGATGTCACCTGAAATCGTTGGCGAGAAAATCGAACCT
GAGAGGTTATATGATGCTGTCAATTTCATACTCTCTCTTCAGGACAAAACTACTGGTGGATTAGCAGTTTGGGA
AAAAGCCGGTGCCTCGTTGTTATTGGAGTGGCTCAATCCTGTCGAGTTTCTTGAGGACCTTATTGTCGAGCATA
CGTACGTCGAATGCACTGCTTCAGCAATTGAAGCATTTGTTTTGTTTAGGAAATTATACCCACATCACCGAAAG
AAGGAGATTGATAATTTCATTGTAAAAGCTGTACAGTATATTGAACACGAACAAACTGCCGATGGTTCATGGT
ATGGAAATTGGGGAATTTGCTTCCTATACGGTTCATGTTTTGCACTTGGAGGCTTGGCTGCTGCTGGCAAAACT
TACCACAATTGTGAAGCCATTCGTAGAGGAGTTGATTTTCTGCTAAAAGCACAAAGTGATGATGGTGGCTGGG
GAGAGAGCTACCAGTCATGCCCAAATAAGATATATACACCACTTGATGGGAAGAGATCAACTGTGGTACACAC
TGCATTGGCTGTCCTTGGTTTAATTCATGCCGGGCAGGCTGAGAGAGACGCCACTCCTATTCATCGCGGTGTCA
AGTTTTTGATCAACTCTCATTTGGAAAATGGAGACTTCCCACAACAGGAAATTATGGGAGTTTTCATGAGAAAC
TGCATGTTACATTATGCACTATACAAGAATACTTTTCCATTGTGGGCTTTAGCTGAATACCGAAGGAAAGTTCC
ATTGCCTAATTGAAAAGACAATTAAAAAAAATGTGCATAGAGATTTCGTGCATCATGGATTCATGCTGATCACT
TGCTCTACTACATTAATGTAATCGACGGAGCAAATGCTATGATGTTTTTTTATTTTATTTTTATAATAATAATAAA
AAATAAGTAATATTCCGGAATCAAAAAAAAAAAAAAAAAAAAAAAAAAAAAAAAA

>TRINITY_DN3249_c0_g1_i5 >TRINITY_DN3249_c0_g1_i5 len=2736 path=[0:0-802 2:803-837 3:838-881 5:882-908 6:909-1001 7:1002-1053 9:1054-1620 10:1621-1794 12:1795-1838 14:1839-1899 17:1900-1938 18:1939-1968 21:1969-2021 23:2022-2046 26:2047-2078 28:2079-2150 30:2151-2160 33:2161-2252 34:2253-2332 36:2333-2408 38:2409-2420 39:2421-2527 41:2528-2735]

ATAAATATGATGTTCTATTTTTCTCCTTGAACATACTGCATCGTTTCAAAAAAATCTCTAGCTTTTGCAGCCAACG
ACATCACCAACTCCAACACCAACCCCCTCTTCTTTCCTTGCTCTTGCCAACTGATTCTAAGTCAAGGGGGACGAT
AATATTATTATGTGGAGGCTTAAGATTGCAGAGGGTGACAAAAATAGCCCATACATTTTTACAACAAACAATTT
TGTGGGAAGGCAAATATGGGAATTTGATCCAAATTATGCTGCCTCGCCGGAAGAGCTAGCTGAAGTTGAAGA
GGCTCGCCAGAAGTTTCACAAAAATCGCCACAAGGTCAAGCCTGCCAGTGATCTTATGTGGCGGCTACAGTTC
CTTAGAGAGAAAAACTTCAAGCAAACAATCCCTCCAGTAAAGGTTAAGGATGAGGAGGAAATCACGTATGAA
ACGGCAACCAAGGCAGTGAAGAGGGCTGCTAGCTATTTTTCAGCCATACAGGCTAACGATGGCCACTGGCCTG
CTGAAAATGCTGGCCCTATGTACTTCCTTCCTCCATTTGTCTTCTGCCTGTACATTACAGGGCATCTTGATGCTGT
ATTTACAGCTGAGCACAAAAAAGAAATCCTTCGCTATTTGTACAATCATCAGCATGAAGATGGTGGATGGGGA
ATACACATAGAAGGCCACAGCAGCATGTTTGGCACAGTTTACGGCTACATTACTATGCGTTTACTTGGATTAGG
ACCCAATGATGGTGAAAACAATGCTTGTGCAAGAGCACGAAAATGGATTCGCGACAATGGTGGTGTCACATAC
ATACCCTCCTGGGGAAAGAATTGGCTGTCGATACTTGGATTGTTTGAATGGGCTGGAACCCACCCAATGCCCC
CTGAATTCTGGTTGCTCCCTTCTTGTTTTCCATTGCATCCAGCACAAATGTGGTGCTATTGCCGACTTGTTTACAT
GCCATTATCTTATTTGTATGGGAAAAGATTTGTTGGTCCAATTACTCCACTTATTCAACAATTGAGGAAAGAACT
TCACACTCAGCCTTACAATGAAATAAATTGGAGGAAAGTTCGTCATTTATGTGCAAAGCCGGATCTCTACTATC
CCCACACCGTGGTACAGAACATACTTTGGGATGGTATGTACATGGCAACAGAGCCTCTCCTAACTCGTTGGCCT
TTGAACAAGTATCTTAGACAGAAGGCTTTAAAAGAAACAATGAAGATCATTCATTATGAAGACCAAAGTAGTA
GATACATTACCATAGGAAGTGTAGAAAAGCCTTTATGTATGCTTGCTTGTTGGGTTGAAGATCCCGATGGTGTT
GCCTTTAAGAAGCATCTTGCTAGAGTTTCAGATTACTTCTGGCTTGGAGAAGATGGAATGAAAGCTCAGACTTT
TGGAAGTCAAACATGGGATACTGCTCTTGGCCTTCAAGCTTTGCTTGCTTGCGATCTCGTCGATGAAATTGCAC
CTACTCTTGCAAAAGGACACGACTACTTAAAGAAAGCTCAGGTGAGGGATAATCCAATAGGCGATTATACAAG
CAATTTCCGTCACTTTTCTAAAGGAGCGTGGACTTTCTCTGATCAAGATCATGGATGGCAAGTTTCGGACTGTA
CAGCAGAAAGTTTGAAGTGTTGCCTAAATTTCTCAATGATGTCACCTGAAATCGTTGGCGAGAAAATCGAACCT
GAGAGGTTATATGATGCTGTCAATTTCATACTCTCTCTTCAGGACAAAACTACTGGTGGATTAGCAGTTTGGGA
AAAAGCCGGTGCCTCGTTGTTATTGGAGTGGCTCAATCCTGTCGAGTTTCTTGAGGACCTTATTGTCGAGCATA
CGTACGTCGAATGCACTGCTTCAGCAATTGAAGCATTTGTTTTGTTTAGGAAATTATACCCACATCACCGAAAG
AAGGAGATTGATAATTTCATTGTAAAAGCTGTACAGTATATTGAACACGAACAAACTGCCGATGGTTCATGGT
ATGGAAATTGGGGAATTTGCTTCCTATACGGTTCATGTTTTGCACTTGGAGGCTTGGCTGCTGCTGGCAAAACT
TACCACAATTGTGAAGCCATTCGTAGAGGAGTTGATTTTCTGCTAAAAGCACAAAGTGATGATGGTGGCTGGG
GAGAGAGCTATCGATCGTGTCCAAATAAGATATATACACCACTTGATGGGAAGAGATCAACTGTGGTACACAC
TGCATTGGCTGTCCTTGGTTTAATTCATGCCGGGCAGGCAGAGAGAGACCCAACACCTATTCATCGTGGTGTA
AAGTATTTGATCAACTCTCAATTGGAAAAGGGAGACTTCCCGCAAGAGGAAATTATGGGAGTTTTCATGAGAA
ACTGCATGTTACATTATGCACTATACAAGAATACTTTTCCATTGTGGGCTTTAGCTGAATATCGACAGAAAGTTC
CATTGCCTAATTGAATGATCTATTCACTCTTGAAATCTATAAGAAGACGATAAAAGAATTGTGCATAGATACTTC
GTGCATGTCTCCATCCTGATCAATGGATTTACAACATTGTAATTGACGGAGCAAAAGGCGAAGATGATAATTTA
CAGTAATAATGATAATAATATTCTTGAATCAAGTATTTGTTGCAACTTATCACATCTTTCTCTCAAGCTTACGTTT
GCGCAAGATTTAGCTCATGTTTGATATTATGTTGAATTTTCAAATAATTAATTTTAGTCTAGATTTTGAATTAAA
A

>TRINITY_DN3249_c0_g1_i6 >TRINITY_DN3249_c0_g1_i6 len=2736 path=[0:0-802 2:803-837 3:838-881 5:882-908 6:909-1001 8:1002-1053 9:1054-1620 11:1621-1794 12:1795-1838 13:1839-1881 15:1882-1899 17:1900-1938 19:1939-1942 20:1943-1968 21:1969-2021 22:2022-2027 24:2028-2046 26:2047-2078 27:2079-2100 29:2101-2157 32:2158-2160 33:2161-2252 34:2253-2332 36:2333-2408 38:2409-2420 39:2421-2527 41:2528-2735]

ATAAATATGATGTTCTATTTTTCTCCTTGAACATACTGCATCGTTTCAAAAAAATCTCTAGCTTTTGCAGCCAACG
ACATCACCAACTCCAACACCAACCCCCTCTTCTTTCCTTGCTCTTGCCAACTGATTCTAAGTCAAGGGGGACGAT
AATATTATTATGTGGAGGCTTAAGATTGCAGAGGGTGACAAAAATAGCCCATACATTTTTACAACAAACAATTT
TGTGGGAAGGCAAATATGGGAATTTGATCCAAATTATGCTGCCTCGCCGGAAGAGCTAGCTGAAGTTGAAGA
GGCTCGCCAGAAGTTTCACAAAAATCGCCACAAGGTCAAGCCTGCCAGTGATCTTATGTGGCGGCTACAGTTC
CTTAGAGAGAAAAACTTCAAGCAAACAATCCCTCCAGTAAAGGTTAAGGATGAGGAGGAAATCACGTATGAA
ACGGCAACCAAGGCAGTGAAGAGGGCTGCTAGCTATTTTTCAGCCATACAGGCTAACGATGGCCACTGGCCTG
CTGAAAATGCTGGCCCTATGTACTTCCTTCCTCCATTTGTCTTCTGCCTGTACATTACAGGGCATCTTGATGCTGT
ATTTACAGCTGAGCACAAAAAAGAAATCCTTCGCTATTTGTACAATCATCAGCATGAAGATGGTGGATGGGGA
ATACACATAGAAGGCCACAGCAGCATGTTTGGCACAGTTTACGGCTACATTACTATGCGTTTACTTGGATTAGG
ACCCAATGATGGTGAAAACAATGCTTGTGCAAGAGCACGAAAATGGATTCGCGACAATGGTGGTGTCACATAC
ATACCCTCCTGGGGAAAGAATTGGCTGTCGATACTTGGATTGTTTGAATGGGCTGGAACCCACCCAATGCCCC
CTGAATTCTGGTTGCTCCCTTCTTGTTTTCCATTGCATCCAGCACAAATGTGGTGCTATTGCCGACTTGTTTACAT
GCCATTATCTTATTTGTATGGGAAAAGATTTGTTGGTCCAATCACTCCGCTTATTCAACAATTGAGGAATGAACT
TCACACTCAGCCGTACAAGGAAATAAATTGGAGGAAAGTTCGTCATTTATGTGCAAAGCCGGATCTCTACTATC
CCCACACCGTGGTACAGAACATACTTTGGGATGGTATGTACATGGCAACAGAGCCTCTCCTAACTCGTTGGCCT
TTGAACAAGTATCTTAGACAGAAGGCTTTAAAAGAAACAATGAAGATCATTCATTATGAAGACCAAAGTAGTA
GATACATTACCATAGGAAGTGTAGAAAAGCCTTTATGTATGCTTGCTTGTTGGGTTGAAGATCCCGATGGTGTT
GCCTTTAAGAAGCATCTTGCTAGAGTTTCAGATTACTTCTGGCTTGGAGAAGATGGAATGAAAGCTCAGACTTT
TGGAAGTCAAACATGGGATACTGCTCTTGGCCTTCAAGCTTTGCTTGCTTGCGATCTCGTCGATGAAATTGCAC
CTACTCTTGCAAAAGGACACGACTACTTAAAGAAAGCTCAGGTGAGGGATAATCCAATAGGCGATTATACAAG
CAATTTCCGTCACTTTTCTAAAGGAGCGTGGACTTTCTCTGATCAAGATCATGGATGGCAAGTTTCGGACAGTA
CAGCAGAGAGTTTGAAGTGTGTCCTACATTTCTCAATGATGCCACCTGAAATCGTTGGCGAAAAAATCGAGCC
TAAGAAGTTATACGATACTGTCAATTTCCTACTCTCTCTTCAGGATAAAACTACTGGCGGATTAGCAATTTGGG
AGAAAGCCGGTGCCTCATTGTTGTTGGAGTGGCTCAATCCTGTCGAGTTTCTTGAGGACCTTATTGTTGAGCAT
ACGTACGTTGAGTGCACTGCTTCAGCAATTGAGGCACTTATTTTCTTTAAGAAGTTATACCCACATCACCGAAA
GAAGGAGATTGATAATTTCCTCATTAAAGCTGTACAGTATATTGAACATGAACAAACTGCCGATGGTTCATGGT
ATGGAAATTGGGGAATTTGCTTCCTATATGGTTCATGTTTTGCTCTAGGAGGTTTGGCTGCTGCTGGCAAAACT
TACCACAATTGCGAAGCAGTTCGTAAAGGAGTCGATTTTCTGTTAAAAGCACAAAGTGGTGATGGCGGTTGGG
GAGAGAGCTACCGATCGTGCCCAAATAAGATATATACACCACTTGATGGGAAGAGATCAACTGTGGTACACAC
TGCATTGGCTGTCCTTGGTTTAATTCATGCCGGGCAGGCAGAGAGAGACCCAACACCTATTCATCGTGGTGTA
AAGTATTTGATCAACTCTCAATTGGAAAAGGGAGACTTCCCGCAAGAGGAAATTATGGGAGTTTTCATGAGAA
ACTGCATGTTACATTATGCACTATACAAGAATACTTTTCCATTGTGGGCTTTAGCTGAATATCGACAGAAAGTTC
CATTGCCTAATTGAATGATCTATTCACTCTTGAAATCTATAAGAAGACGATAAAAGAATTGTGCATAGATACTTC
GTGCATGTCTCCATCCTGATCAATGGATTTACAACATTGTAATTGACGGAGCAAAAGGCGAAGATGATAATTTA
CAGTAATAATGATAATAATATTCTTGAATCAAGTATTTGTTGCAACTTATCACATCTTTCTCTCAAGCTTACGTTT
GCGCAAGATTTAGCTCATGTTTGATATTATGTTGAATTTTCAAATAATTAATTTTAGTCTAGATTTTGAATTAAA
A

>TRINITY_DN3249_c0_g1_i9 >TRINITY_DN3249_c0_g1_i9 len=2640 path=[0:0-802 2:803-837 3:838-881 4:882-908 6:909-1001 8:1002-1053 9:1054-1620 10:1621-1794 12:1795-1838 14:1839-1899 17:1900-1938 18:1939-1968 21:1969-2021 23:2022-2046 26:2047-2078 28:2079-2150 31:2151-2157 32:2158-2160 33:2161-2252 35:2253-2332 36:2333-2408 38:2409-2420 40:2421-2639]

ATAAATATGATGTTCTATTTTTCTCCTTGAACATACTGCATCGTTTCAAAAAAATCTCTAGCTTTTGCAGCCAACG
ACATCACCAACTCCAACACCAACCCCCTCTTCTTTCCTTGCTCTTGCCAACTGATTCTAAGTCAAGGGGGACGAT
AATATTATTATGTGGAGGCTTAAGATTGCAGAGGGTGACAAAAATAGCCCATACATTTTTACAACAAACAATTT
TGTGGGAAGGCAAATATGGGAATTTGATCCAAATTATGCTGCCTCGCCGGAAGAGCTAGCTGAAGTTGAAGA
GGCTCGCCAGAAGTTTCACAAAAATCGCCACAAGGTCAAGCCTGCCAGTGATCTTATGTGGCGGCTACAGTTC
CTTAGAGAGAAAAACTTCAAGCAAACAATCCCTCCAGTAAAGGTTAAGGATGAGGAGGAAATCACGTATGAA
ACGGCAACCAAGGCAGTGAAGAGGGCTGCTAGCTATTTTTCAGCCATACAGGCTAACGATGGCCACTGGCCTG
CTGAAAATGCTGGCCCTATGTACTTCCTTCCTCCATTTGTCTTCTGCCTGTACATTACAGGGCATCTTGATGCTGT
ATTTACAGCTGAGCACAAAAAAGAAATCCTTCGCTATTTGTACAATCATCAGCATGAAGATGGTGGATGGGGA
ATACACATAGAAGGCCACAGCAGCATGTTTGGCACAGTTTACGGCTACATTACTATGCGTTTACTTGGATTAGG
ACCCAATGATGGTGAAAACAATGCTTGTGCAAGAGCACGAAAATGGATTCGCGACAATGGTGGTGTCACATAC
ATACCCTCCTGGGGAAAGAATTGGCTGTCGATACTTGGATTGTTTGAATGGGCTGGAACCCACCCAATGCCGC
CTGAGTTCTGGCTGCTCCCTTCTTATTTTCCATTGCATCCAGCACAAATGTGGTGCTATTGCCGACTTGTTTACAT
GCCATTATCTTATTTGTATGGGAAAAGATTTGTTGGTCCAATCACTCCGCTTATTCAACAATTGAGGAATGAACT
TCACACTCAGCCGTACAAGGAAATAAATTGGAGGAAAGTTCGTCATTTATGTGCAAAGCCGGATCTCTACTATC
CCCACACCGTGGTACAGAACATACTTTGGGATGGTATGTACATGGCAACAGAGCCTCTCCTAACTCGTTGGCCT
TTGAACAAGTATCTTAGACAGAAGGCTTTAAAAGAAACAATGAAGATCATTCATTATGAAGACCAAAGTAGTA
GATACATTACCATAGGAAGTGTAGAAAAGCCTTTATGTATGCTTGCTTGTTGGGTTGAAGATCCCGATGGTGTT
GCCTTTAAGAAGCATCTTGCTAGAGTTTCAGATTACTTCTGGCTTGGAGAAGATGGAATGAAAGCTCAGACTTT
TGGAAGTCAAACATGGGATACTGCTCTTGGCCTTCAAGCTTTGCTTGCTTGCGATCTCGTCGATGAAATTGCAC
CTACTCTTGCAAAAGGACACGACTACTTAAAGAAAGCTCAGGTGAGGGATAATCCAATAGGCGATTATACAAG
CAATTTCCGTCACTTTTCTAAAGGAGCGTGGACTTTCTCTGATCAAGATCATGGATGGCAAGTTTCGGACTGTA
CAGCAGAAAGTTTGAAGTGTTGCCTAAATTTCTCAATGATGTCACCTGAAATCGTTGGCGAGAAAATCGAACCT
GAGAGGTTATATGATGCTGTCAATTTCATACTCTCTCTTCAGGACAAAACTACTGGTGGATTAGCAGTTTGGGA
AAAAGCCGGTGCCTCGTTGTTATTGGAGTGGCTCAATCCTGTCGAGTTTCTTGAGGACCTTATTGTCGAGCATA
CGTACGTCGAATGCACTGCTTCAGCAATTGAAGCATTTGTTTTGTTTAGGAAATTATACCCACATCACCGAAAG
AAGGAGATTGATAATTTCATTGTAAAAGCTGTACAGTATATTGAACACGAACAAACTGCCGATGGTTCATGGT
ATGGAAATTGGGGAATTTGCTTCCTATACGGTTCATGTTTTGCACTTGGAGGCTTGGCTGCTGCTGGCAAAACT
TACCACAATTGTGAAGCCATTCGTAGAGGAGTTGATTTTCTGCTAAAAGCACAAAGTGATGATGGTGGCTGGG
GAGAGAGCTACCAGTCATGCCCAAATAAGATATATACACCACTTGATGGGAAGAGATCAACTGTGGTACACAC
TGCATTGGCTGTCCTTGGTTTAATTCATGCCGGGCAGGCTGAGAGAGACGCCACTCCTATTCATCGCGGTGTCA
AGTTTTTGATCAACTCTCATTTGGAAAATGGAGACTTCCCACAACAGGAAATTATGGGAGTTTTCATGAGAAAC
TGCATGTTACATTATGCACTATACAAGAATACTTTTCCATTGTGGGCTTTAGCTGAATACCGAAGGAAAGTTCC
ATTGCCTAATTGAAAAGACAATTAAAAAAAATGTGCATAGAGATTTCGTGCATCATGGATTCATGCTGATCACT
TGCTCTACTACATTAATGTAATCGACGGAGCAAATGCTATGATGTTTTTTTATTTTATTTTTATAATAATAATAAA
AAATAAGTAATATTCCGGAATCAAAAAAAAAAAAAAAAAAAAAAAAAAAAAAA

>TRINITY_DN88_c0_g1_i10 >TRINITY_DN88_c0_g1_i10 len=2475 path=[0:0-303 4:304-448 6:449-451 9:452-507 12:508-541 15:542-732 18:733-845 21:846-967 28:968-993 29:994-1130 31:1131-1154 33:1155-1184 34:1185-1209 36:1210-1395 38:1396-1471 39:1472-1950 40:1951-1986 42:1987-2474]

CAAAGTTTGTCATTATCACGTAAGATTTAAAAATACATAATGCGGTGTTCGCCAGCTGCAGCTTTACAAAGCTA
CGGCCATTCAGCCGCTAGCCAGGCGCGCCCCACATATTGACTGGGTTACGCCACGTGCGCGACTGTCGTTTAC
ATTCACTGCTACTACTCGCCTGATTTTCACATCAAAACACATTAGCAAACTTAACATCCACAAACATTTCAGGAA
AAAAAAAAAATAAAACTAAACATCCACAAAAGTGCCGTTGCACTCAAGTACCAGTCTACTCTTTTCTCCCTAATT
ATTAGGTAATGATCTCGCTTTCTCCTCGATTCCTCATCACTTTCTGAATCGGATATTCGAAAAGCTTTCACTCATG
TCTGTTAATGCGTCAATAAAGCGTGTGAAAAATTAGCCTACGTGTCTCGCCGTTGTGTTATTTCTGTTTTGAAGG
TTTAAAGATGTGGAAGCTGAAGATCGCCGAGGGAGGGAATGCGTGGCTGCGAACGACGAATAATCACGTCG
GAAGACAAGTTTGGGAGTTCGACCCGAAGCTCGGATCGCCGGAGGAGCTGGCGGAGATCGAAAAAGCTCGT
CAAAATTTCTACAATAATCGCTTCGAGATGAAGCACAGCGCCGATCTACTCATGCGCATTCAGTTTGCTAAGGA
GAATCCAGGTGTTACAGTTATACCTCAAGTCAAAGTAAAAGATACAGAAGATGTTACTGAGGAGATAGTGAAA
ACCACGTTAAGAAGGGCTATAAGTTACTATTCAACCATCCAGGCCGATGATGGGCACTGGCCTGGCGATTATG
GAGGTCCTATGTTTCTCTTACCTGGTTTAGTGATTGCTCTGTCTATCACTGGAGCACTGAATGCAGTTCTATCTG
ACGTACATCAAAAGGAGATGTGCCGTTATCTCTACAATCACCAGAACAGAGACGGTGGGTGGGGTTTGCACAT
CGAGGGCCCAAGCACTATGTTTGGTACAGTTTTGAGTTATATTACTTTAAGGTTGCTTGGTGAAGGAGCTAATG
ATGGGCAAGGAGCAATGGAGAGAGGGCGTAACTGGATTCTAAGTCATGGTAGTGCAACTGCGATAACATCAT
GGGGAAAAATGTGGCTCTCAGTACTTGGAGCTTTTGAATGGTCTGGCAATAATCCTCTTCCCCCTGAGATATGG
CTTCTTACATATATGCTCCCATTCCATCCAGGAAGGATGTGGTGTCACTGTCGCATGGTCTATTTGCCAATGTCT
TACTTGTATGGAAAGAGGTTTGTTGGTCCAATCACCCCAACAGTATTGTCTTTGAGAAAGGAACTTTACAATGT
CCCATATCATGAAATAGATTGGAGTGAAGCACGTAACCTATGTGCAAAGGAAGATTTGTACTACCCACATCCAC
TAATCCAGGATGTACTTTGGACAACACTTGACAAAGTCCTTGAACCTATTTTATTGCACTGGCCTGGAAAAAAG
TTGAGAGAAAAGGCTCTCCGCACTGTAATGGATCACATACATTATGAGGATGAGAATACTCGTTACATTTGCAT
AGGACCTGTGAACAAGGTTTTGAATATGCTTTGCTGTTGGGTGGAAGATCCGAACTCAGAGGCTTTCAAGTTA
CATATACCAAGAATCTATGATTATCTGTGGATTGCTGAAGATGGCATGAAAATGCAGGGTTATAATGGAAGTC
AATTATGGGATACTGCTTTTGCTGTTCAAGCAATAATATCAACCAATCTTGTTGAAGAATATGGTGCAACTCTG
AAGAAAGCACATATGTACATAAAGAACTCACAGGTTTTAGAAGATTGCCCCGGTGATCTTGATTTTTGGTACCG
TCACATTTCAAAAGGTGCTTGGCCCTTCTCAACTGCAGATCACGGATGGCCTATCTCAGACTGTACAGCAGAGG
GTTTGAAAGCTGCTTTGTTATTATCAAAATTACCATCAGAAGTTGTTGGCGAACCGTTAGAGGCAAAACAGTTA
TATGATGCTGTGAATGTTATTCTTTCGTTACAGAATGCTGATGGTGGCTTTGCAACGTATGAGCTCACTAGATC
CTATCCTTGGTTGGAGTTCATCAATCCTGCTGAAACTTTTGGTGATATCGTGATTGATTATCCTTATGTGGAATG
TACCTCAGCTGCAATACAAGCTTTGACATCCTTTAAGAAATTATATCCTGGGCATCGACGGGAAGAAATAGATG
ATTGTATCAGAAAAGCTGTCATGTTTATCGAAAAGATTCAAGAATCAGATGGCTCATGGTATGGCTCATGGGG
TGTATGCTTCACTTATGGTGCATGGTTTGGGGTGAGAGGCTTAATGGCTGCTGGAATGAACTACAGTAATTGC
TCTAGCATCCGTAAAGCTTGTGATTTTCTGCTGTCAAAACAGCTTCCATCTGGTGGCTGGGGAGAGAGTTACCT
TTCATGTCAAAATAAGGTTTATTCAAATCTTGAAGGGGACA

>TRINITY_DN88_c0_g1_i13 >TRINITY_DN88_c0_g1_i13 len=3207 path=[0:0-303 4:304-448 6:449-451 9:452-507 11:508-541 15:542-732 17:733-845 21:846-967 28:968-993 29:994-1130 31:1131-1154 33:1155-1184 34:1185-1209 36:1210-1395 38:1396-1471 39:1472-1950 41:1951-1986 42:1987-2474 44:2475-2608 45:2609-2762 47:2763-2917 48:2918-2946 49:2947-3206]

CAAAGTTTGTCATTATCACGTAAGATTTAAAAATACATAATGCGGTGTTCGCCAGCTGCAGCTTTACAAAGCTA
CGGCCATTCAGCCGCTAGCCAGGCGCGCCCCACATATTGACTGGGTTACGCCACGTGCGCGACTGTCGTTTAC
ATTCACTGCTACTACTCGCCTGATTTTCACATCAAAACACATTAGCAAACTTAACATCCACAAACATTTCAGGAA
AAAAAAAAAATAAAACTAAACATCCACAAAAGTGCCGTTGCACTCAAGTACCAGTCTACTCTTTTCTCCCTAATT
ATTAGGTAATGATCTCGCTTTCTCCTCGATTCCTCATCACTTTCTGAATCGGATATTCGAAAAGCTTTCACTCATG
TCTGTTAATGCGTCAATAAAGCGTGTGAAAAATTAGCCTACGTGTCTCGCCGTTGTGTTATTTCTGTTTTGAAGG
TTTAAAGATGTGGAAGCTGAAGATCGCCGAGGGAGGGAATGCGTGGCTGCGAACGACGAACAATCATGTTG
GAAGACAAGTCTGGGAGTTCGATCCGAAGCTCGGATCGCCGGAGGAGCTGGCGGAGATCGAAAAAGCTCGT
CAAAATTTCTACAATAATCGCTTCGAGATGAAGCACAGCGCCGATCTACTCATGCGCATTCAGTTTGCTAAGGA
GAATCCAGGTGTTACAGTTATACCTCAAGTCAAAGTAAAAGATACAGAAGATGTTACTGAGGAGATAGTAAAA
AACACATTAAGAAAGGCTATAAGTTACTATTCAACTATCCAGGCCCATGATGGGCACTGGCCTGGGGATTATG
GAGGTCCTATGTTTCTTTTGCCTGGTTTAGTGATTACTCTGTCTATCACTGGAGCACTGAATGCAGTTCTATCTG
ACGTACATCAAAAGGAGATGTGCCGTTATCTCTACAATCACCAGAACAGAGACGGTGGGTGGGGTTTGCACAT
CGAGGGCCCAAGCACTATGTTTGGTACAGTTTTGAGTTATATTACTTTAAGGTTGCTTGGTGAAGGAGCTAATG
ATGGGCAAGGAGCAATGGAGAGAGGGCGTAACTGGATTCTAAGTCATGGTAGTGCAACTGCGATAACATCAT
GGGGAAAAATGTGGCTCTCAGTACTTGGAGCTTTTGAATGGTCTGGCAATAATCCTCTTCCCCCTGAGATATGG
CTTCTTACATATATGCTCCCATTCCATCCAGGAAGGATGTGGTGTCACTGTCGCATGGTCTATTTGCCAATGTCT
TACTTGTATGGAAAGAGGTTTGTTGGTCCAATCACCCCAACAGTATTGTCTTTGAGAAAGGAACTTTACAATGT
CCCATATCATGAAATAGATTGGAGTGAAGCACGTAACCTATGTGCAAAGGAAGATTTGTACTACCCACATCCAC
TAATCCAGGATGTACTTTGGACAACACTTGACAAAGTCCTTGAACCTATTTTATTGCACTGGCCTGGAAAAAAG
TTGAGAGAAAAGGCTCTCCGCACTGTAATGGATCACATACATTATGAGGATGAGAATACTCGTTACATTTGCAT
AGGACCTGTGAACAAGGTTTTGAATATGCTTTGCTGTTGGGTGGAAGATCCGAACTCAGAGGCTTTCAAGTTA
CATATACCAAGAATCTATGATTATCTGTGGATTGCTGAAGATGGCATGAAAATGCAGGGTTATAATGGAAGTC
AATTATGGGATACTGCTTTTGCTGTTCAAGCAATAATATCAACCAATCTTGTTGAAGAATATGGTGCAACTCTG
AAGAAAGCACATATGTACATAAAGAACTCACAGGTTTTAGAAGATTGCCCCGGTGATCTTGATTTTTGGTACCG
TCACATTTCAAAAGGTGCTTGGCCCTTCTCAACTGCAGATCACGGATGGCCTATCTCAGACTGTACAGCAGAGG
GTTTGAAAGCTGCTTTGTTATTATCAAAATTACCTTCGGAAGTTGTTGGTGAACCATTAGAGGCAAAACGGTTA
TATGATGCTGTGAATGTTATTCTTTCGTTACAGAATGCTGATGGTGGCTTTGCAACGTATGAGCTCACTAGATC
CTATCCTTGGTTGGAGTTCATCAATCCTGCTGAAACTTTTGGTGATATCGTGATTGATTATCCTTATGTGGAATG
TACCTCAGCTGCAATACAAGCTTTGACATCCTTTAAGAAATTATATCCTGGGCATCGACGGGAAGAAATAGATG
ATTGTATCAGAAAAGCTGTCATGTTTATCGAAAAGATTCAAGAATCAGATGGCTCATGGTATGGCTCATGGGG
TGTATGCTTCACTTATGGTGCATGGTTTGGGGTGAGAGGCTTAATGGCTGCTGGAATGAACTACAGTAATTGC
TCTAGCATCCGTAAAGCTTGTGATTTTCTGCTGTCAAAACAGCTTCCATCTGGTGGCTGGGGAGAGAGTTACCT
TTCATGTCAAAATAAGGTTTATTCAAATCTTGAAGGGGACAAGCCCCATTTAGTAAATACTGGATGGGCTATGC
TGGCTCTTATCGATGCTGAGCAGGCTGAGAGAGATCCAACACCATTGCACCGTGCTGCAAGATATTTGATAAA
TTCACAAGTGGAGAATGGAGATTTCCTGCAGCAGGAAATCATGGGAGTCTTCAATAAGAATTGTATGATCACT
TACGCTGCATATAGAAACATTTTCCCTATTTGGGCACTAGGAGAGTACCGATGTCGGGTATTGCAGGTTTCCAA
GTAAACATTTTTATCATTTCAAATGCAATCGTCCATATGTTTTATTATTTTTCTACTTAAAAAGACTTTTAAGCCTT
TTCATTCCCCGTTCATTTTGAGCATTACCAGTAATGCATGTAGAAGTTTCCAGCTAATAGACCAGCAGATAAAA
AAGCTGTAGAAGTGGACTTTGTATCAAAAGAATAGTCCGGTGTATGTTTTGTTTTGCATGAATGAATTGAGAAC
TCCGGAACTCAGGTTCGGGGATCCCTTTTCTTAATGATGGCCGACAGGATTGGTTGATTGAAATTTATATTCCC
TTTTAAGCGCGTATTCATTTGTGGAAATTAAAGATGACACTTGACTTTTCTACATTACAAGTCTCGGTAACGATC
ATCAGAAGTAGATACCAGATAGAATAAATAAATAAAATAGCTTTGCAGAAGTAGAAAAGTTGAAATGATTGTG
AACGAGAACGACGCTTGACAAATTAATAAACC

>TRINITY_DN88_c0_g1_i14 >TRINITY_DN88_c0_g1_i14 len=3062 path=[0:0-303 6:304-306 9:307-362 11:363-396 15:397-587 17:588-700 21:701-822 28:823-848 29:849-985 31:986-1009 33:1010-1039 34:1040-1064 36:1065-1250 38:1251-1326 39:1327-1805 41:1806-1841 42:1842-2329 44:2330-2463 45:2464-2617 47:2618-2772 48:2773-2801 49:2802-3061]

CAAAGTTTGTCATTATCACGTAAGATTTAAAAATACATAATGCGGTGTTCGCCAGCTGCAGCTTTACAAAGCTA
CGGCCATTCAGCCGCTAGCCAGGCGCGCCCCACATATTGACTGGGTTACGCCACGTGCGCGACTGTCGTTTAC
ATTCACTGCTACTACTCGCCTGATTTTCACATCAAAACACATTAGCAAACTTAACATCCACAAACATTTCAGGAA
AAAAAAAAAATAAAACTAAACATCCACAAAAGTGCCGTTGCACTCAAGTACCAGTCTACTCTTTTCTCCCTAATT
ATTAGGTTTAAAGATGTGGAAGCTGAAGATCGCCGAGGGAGGGAATGCGTGGCTGCGAACGACGAACAATC
ATGTTGGAAGACAAGTCTGGGAGTTCGATCCGAAGCTCGGATCGCCGGAGGAGCTGGCGGAGATCGAAAAA
GCTCGTCAAAATTTCTACAATAATCGCTTCGAGATGAAGCACAGCGCCGATCTACTCATGCGCATTCAGTTTGC
TAAGGAGAATCCAGGTGTTACAGTTATACCTCAAGTCAAAGTAAAAGATACAGAAGATGTTACTGAGGAGATA
GTAAAAAACACATTAAGAAAGGCTATAAGTTACTATTCAACTATCCAGGCCCATGATGGGCACTGGCCTGGGG
ATTATGGAGGTCCTATGTTTCTTTTGCCTGGTTTAGTGATTACTCTGTCTATCACTGGAGCACTGAATGCAGTTC
TATCTGACGTACATCAAAAGGAGATGTGCCGTTATCTCTACAATCACCAGAACAGAGACGGTGGGTGGGGTTT
GCACATCGAGGGCCCAAGCACTATGTTTGGTACAGTTTTGAGTTATATTACTTTAAGGTTGCTTGGTGAAGGAG
CTAATGATGGGCAAGGAGCAATGGAGAGAGGGCGTAACTGGATTCTAAGTCATGGTAGTGCAACTGCGATAA
CATCATGGGGAAAAATGTGGCTCTCAGTACTTGGAGCTTTTGAATGGTCTGGCAATAATCCTCTTCCCCCTGAG
ATATGGCTTCTTACATATATGCTCCCATTCCATCCAGGAAGGATGTGGTGTCACTGTCGCATGGTCTATTTGCCA
ATGTCTTACTTGTATGGAAAGAGGTTTGTTGGTCCAATCACCCCAACAGTATTGTCTTTGAGAAAGGAACTTTA
CAATGTCCCATATCATGAAATAGATTGGAGTGAAGCACGTAACCTATGTGCAAAGGAAGATTTGTACTACCCA
CATCCACTAATCCAGGATGTACTTTGGACAACACTTGACAAAGTCCTTGAACCTATTTTATTGCACTGGCCTGGA
AAAAAGTTGAGAGAAAAGGCTCTCCGCACTGTAATGGATCACATACATTATGAGGATGAGAATACTCGTTACA
TTTGCATAGGACCTGTGAACAAGGTTTTGAATATGCTTTGCTGTTGGGTGGAAGATCCGAACTCAGAGGCTTTC
AAGTTACATATACCAAGAATCTATGATTATCTGTGGATTGCTGAAGATGGCATGAAAATGCAGGGTTATAATG
GAAGTCAATTATGGGATACTGCTTTTGCTGTTCAAGCAATAATATCAACCAATCTTGTTGAAGAATATGGTGCA
ACTCTGAAGAAAGCACATATGTACATAAAGAACTCACAGGTTTTAGAAGATTGCCCCGGTGATCTTGATTTTTG
GTACCGTCACATTTCAAAAGGTGCTTGGCCCTTCTCAACTGCAGATCACGGATGGCCTATCTCAGACTGTACAG
CAGAGGGTTTGAAAGCTGCTTTGTTATTATCAAAATTACCTTCGGAAGTTGTTGGTGAACCATTAGAGGCAAAA
CGGTTATATGATGCTGTGAATGTTATTCTTTCGTTACAGAATGCTGATGGTGGCTTTGCAACGTATGAGCTCAC
TAGATCCTATCCTTGGTTGGAGTTCATCAATCCTGCTGAAACTTTTGGTGATATCGTGATTGATTATCCTTATGT
GGAATGTACCTCAGCTGCAATACAAGCTTTGACATCCTTTAAGAAATTATATCCTGGGCATCGACGGGAAGAA
ATAGATGATTGTATCAGAAAAGCTGTCATGTTTATCGAAAAGATTCAAGAATCAGATGGCTCATGGTATGGCT
CATGGGGTGTATGCTTCACTTATGGTGCATGGTTTGGGGTGAGAGGCTTAATGGCTGCTGGAATGAACTACAG
TAATTGCTCTAGCATCCGTAAAGCTTGTGATTTTCTGCTGTCAAAACAGCTTCCATCTGGTGGCTGGGGAGAGA
GTTACCTTTCATGTCAAAATAAGGTTTATTCAAATCTTGAAGGGGACAAGCCCCATTTAGTAAATACTGGATGG
GCTATGCTGGCTCTTATCGATGCTGAGCAGGCTGAGAGAGATCCAACACCATTGCACCGTGCTGCAAGATATT
TGATAAATTCACAAGTGGAGAATGGAGATTTCCTGCAGCAGGAAATCATGGGAGTCTTCAATAAGAATTGTAT
GATCACTTACGCTGCATATAGAAACATTTTCCCTATTTGGGCACTAGGAGAGTACCGATGTCGGGTATTGCAGG
TTTCCAAGTAAACATTTTTATCATTTCAAATGCAATCGTCCATATGTTTTATTATTTTTCTACTTAAAAAGACTTTT
AAGCCTTTTCATTCCCCGTTCATTTTGAGCATTACCAGTAATGCATGTAGAAGTTTCCAGCTAATAGACCAGCAG
ATAAAAAAGCTGTAGAAGTGGACTTTGTATCAAAAGAATAGTCCGGTGTATGTTTTGTTTTGCATGAATGAATT
GAGAACTCCGGAACTCAGGTTCGGGGATCCCTTTTCTTAATGATGGCCGACAGGATTGGTTGATTGAAATTTAT
ATTCCCTTTTAAGCGCGTATTCATTTGTGGAAATTAAAGATGACACTTGACTTTTCTACATTACAAGTCTCGGTA
ACGATCATCAGAAGTAGATACCAGATAGAATAAATAAATAAAATAGCTTTGCAGAAGTAGAAAAGTTGAAATG
ATTGTGAACGAGAACGACGCTTGACAAATTAATAAACC

>TRINITY_DN88_c0_g1_i2 >TRINITY_DN88_c0_g1_i2 len=2875 path=[0:0-303 6:304-306 9:307-362 11:363-396 15:397-587 17:588-700 21:701-822 28:823-848 29:849-985 31:986-1009 33:1010-1039 34:1040-1064 36:1065-1250 38:1251-1326 39:1327-1805 41:1806-1841 42:1842-2329 43:2330-2463 45:2464-2617 46:2618-2745 48:2746-2774 50:2775-2874]

CAAAGTTTGTCATTATCACGTAAGATTTAAAAATACATAATGCGGTGTTCGCCAGCTGCAGCTTTACAAAGCTA
CGGCCATTCAGCCGCTAGCCAGGCGCGCCCCACATATTGACTGGGTTACGCCACGTGCGCGACTGTCGTTTAC
ATTCACTGCTACTACTCGCCTGATTTTCACATCAAAACACATTAGCAAACTTAACATCCACAAACATTTCAGGAA
AAAAAAAAAATAAAACTAAACATCCACAAAAGTGCCGTTGCACTCAAGTACCAGTCTACTCTTTTCTCCCTAATT
ATTAGGTTTAAAGATGTGGAAGCTGAAGATCGCCGAGGGAGGGAATGCGTGGCTGCGAACGACGAACAATC
ATGTTGGAAGACAAGTCTGGGAGTTCGATCCGAAGCTCGGATCGCCGGAGGAGCTGGCGGAGATCGAAAAA
GCTCGTCAAAATTTCTACAATAATCGCTTCGAGATGAAGCACAGCGCCGATCTACTCATGCGCATTCAGTTTGC
TAAGGAGAATCCAGGTGTTACAGTTATACCTCAAGTCAAAGTAAAAGATACAGAAGATGTTACTGAGGAGATA
GTAAAAAACACATTAAGAAAGGCTATAAGTTACTATTCAACTATCCAGGCCCATGATGGGCACTGGCCTGGGG
ATTATGGAGGTCCTATGTTTCTTTTGCCTGGTTTAGTGATTACTCTGTCTATCACTGGAGCACTGAATGCAGTTC
TATCTGACGTACATCAAAAGGAGATGTGCCGTTATCTCTACAATCACCAGAACAGAGACGGTGGGTGGGGTTT
GCACATCGAGGGCCCAAGCACTATGTTTGGTACAGTTTTGAGTTATATTACTTTAAGGTTGCTTGGTGAAGGAG
CTAATGATGGGCAAGGAGCAATGGAGAGAGGGCGTAACTGGATTCTAAGTCATGGTAGTGCAACTGCGATAA
CATCATGGGGAAAAATGTGGCTCTCAGTACTTGGAGCTTTTGAATGGTCTGGCAATAATCCTCTTCCCCCTGAG
ATATGGCTTCTTACATATATGCTCCCATTCCATCCAGGAAGGATGTGGTGTCACTGTCGCATGGTCTATTTGCCA
ATGTCTTACTTGTATGGAAAGAGGTTTGTTGGTCCAATCACCCCAACAGTATTGTCTTTGAGAAAGGAACTTTA
CAATGTCCCATATCATGAAATAGATTGGAGTGAAGCACGTAACCTATGTGCAAAGGAAGATTTGTACTACCCA
CATCCACTAATCCAGGATGTACTTTGGACAACACTTGACAAAGTCCTTGAACCTATTTTATTGCACTGGCCTGGA
AAAAAGTTGAGAGAAAAGGCTCTCCGCACTGTAATGGATCACATACATTATGAGGATGAGAATACTCGTTACA
TTTGCATAGGACCTGTGAACAAGGTTTTGAATATGCTTTGCTGTTGGGTGGAAGATCCGAACTCAGAGGCTTTC
AAGTTACATATACCAAGAATCTATGATTATCTGTGGATTGCTGAAGATGGCATGAAAATGCAGGGTTATAATG
GAAGTCAATTATGGGATACTGCTTTTGCTGTTCAAGCAATAATATCAACCAATCTTGTTGAAGAATATGGTGCA
ACTCTGAAGAAAGCACATATGTACATAAAGAACTCACAGGTTTTAGAAGATTGCCCCGGTGATCTTGATTTTTG
GTACCGTCACATTTCAAAAGGTGCTTGGCCCTTCTCAACTGCAGATCACGGATGGCCTATCTCAGACTGTACAG
CAGAGGGTTTGAAAGCTGCTTTGTTATTATCAAAATTACCTTCGGAAGTTGTTGGTGAACCATTAGAGGCAAAA
CGGTTATATGATGCTGTGAATGTTATTCTTTCGTTACAGAATGCTGATGGTGGCTTTGCAACGTATGAGCTCAC
TAGATCCTATCCTTGGTTGGAGTTCATCAATCCTGCTGAAACTTTTGGTGATATCGTGATTGATTATCCTTATGT
GGAATGTACCTCAGCTGCAATACAAGCTTTGACATCCTTTAAGAAATTATATCCTGGGCATCGACGGGAAGAA
ATAGATGATTGTATCAGAAAAGCTGTCATGTTTATCGAAAAGATTCAAGAATCAGATGGCTCATGGTATGGCT
CATGGGGTGTATGCTTCACTTATGGTGCATGGTTTGGGGTGAGAGGCTTAATGGCTGCTGGAATGAACTACAG
TAATTGCTCTAGCATCCGTAAAGCTTGTGATTTTCTGCTGTCAAAACAGCTTCCATCTGGTGGCTGGGGAGAGA
GTTACCTTTCATGTCAAAATAAGGTTTATTCAAATCTTGAAGGGGACAGGCCTCATTTAGTAAATACTGGATGG
GCATTATTGGCTCTTATTGATGCTGGGCAGGCTGAGAGAGATCCAGCACCATTGCATCTTGCTGCAAGATATTT
GATAGATTCACAAATGGATAATGGAGATTTCCCACAGCAGGAAATCATGGGAGTCTTCAATAAGAATTGTATG
ATCACTTACGCTGCATATAGAAACATTTTCCCTATTTGGGCACTAGGAGAGTACCGATGTCGGGTATTGCAGGT
TTCCAAGTAAACATTTTTATCATTTCAAATGCAATCGTCCACATGTTTTCTGATTTTTAAGCGTTTTTGTTTCCCTA
TTCATTTTCAGCATTACTAGTAGTGACTTGTAGAAGTTTCCAACTGAAATTGAAAGAGATAAAACAGCTGTTTA
TATCAAAAGAATAGTCCCGTGTATGTTTTGTTTTGCATGAATGAATTTGAAAAGTCCAGAACACAGGTTCAGGA
TCACCTTTCTTAATGATGGCCGACAGCATTGATTGATTGAAATTTATATCTCCTTTTATGCTTATTGATTTGA

>TRINITY_DN88_c0_g1_i3_ >TRINITY_DN88_c0_g1_i3 len=3020 path=[0:0-303 4:304-448 6:449-451 9:452-507 11:508-541 15:542-732 17:733-845 21:846-967 28:968-993 29:994-1130 31:1131-1154 33:1155-1184 34:1185-1209 36:1210-1395 38:1396-1471 39:1472-1950 41:1951-1986 42:1987-2474 43:2475-2608 45:2609-2762 46:2763-2890 48:2891-2919 50:2920-3019]

CAAAGTTTGTCATTATCACGTAAGATTTAAAAATACATAATGCGGTGTTCGCCAGCTGCAGCTTTACAAAGCTA
CGGCCATTCAGCCGCTAGCCAGGCGCGCCCCACATATTGACTGGGTTACGCCACGTGCGCGACTGTCGTTTAC
ATTCACTGCTACTACTCGCCTGATTTTCACATCAAAACACATTAGCAAACTTAACATCCACAAACATTTCAGGAA
AAAAAAAAAATAAAACTAAACATCCACAAAAGTGCCGTTGCACTCAAGTACCAGTCTACTCTTTTCTCCCTAATT
ATTAGGTAATGATCTCGCTTTCTCCTCGATTCCTCATCACTTTCTGAATCGGATATTCGAAAAGCTTTCACTCATG
TCTGTTAATGCGTCAATAAAGCGTGTGAAAAATTAGCCTACGTGTCTCGCCGTTGTGTTATTTCTGTTTTGAAGG
TTTAAAGATGTGGAAGCTGAAGATCGCCGAGGGAGGGAATGCGTGGCTGCGAACGACGAACAATCATGTTG
GAAGACAAGTCTGGGAGTTCGATCCGAAGCTCGGATCGCCGGAGGAGCTGGCGGAGATCGAAAAAGCTCGT
CAAAATTTCTACAATAATCGCTTCGAGATGAAGCACAGCGCCGATCTACTCATGCGCATTCAGTTTGCTAAGGA
GAATCCAGGTGTTACAGTTATACCTCAAGTCAAAGTAAAAGATACAGAAGATGTTACTGAGGAGATAGTAAAA
AACACATTAAGAAAGGCTATAAGTTACTATTCAACTATCCAGGCCCATGATGGGCACTGGCCTGGGGATTATG
GAGGTCCTATGTTTCTTTTGCCTGGTTTAGTGATTACTCTGTCTATCACTGGAGCACTGAATGCAGTTCTATCTG
ACGTACATCAAAAGGAGATGTGCCGTTATCTCTACAATCACCAGAACAGAGACGGTGGGTGGGGTTTGCACAT
CGAGGGCCCAAGCACTATGTTTGGTACAGTTTTGAGTTATATTACTTTAAGGTTGCTTGGTGAAGGAGCTAATG
ATGGGCAAGGAGCAATGGAGAGAGGGCGTAACTGGATTCTAAGTCATGGTAGTGCAACTGCGATAACATCAT
GGGGAAAAATGTGGCTCTCAGTACTTGGAGCTTTTGAATGGTCTGGCAATAATCCTCTTCCCCCTGAGATATGG
CTTCTTACATATATGCTCCCATTCCATCCAGGAAGGATGTGGTGTCACTGTCGCATGGTCTATTTGCCAATGTCT
TACTTGTATGGAAAGAGGTTTGTTGGTCCAATCACCCCAACAGTATTGTCTTTGAGAAAGGAACTTTACAATGT
CCCATATCATGAAATAGATTGGAGTGAAGCACGTAACCTATGTGCAAAGGAAGATTTGTACTACCCACATCCAC
TAATCCAGGATGTACTTTGGACAACACTTGACAAAGTCCTTGAACCTATTTTATTGCACTGGCCTGGAAAAAAG
TTGAGAGAAAAGGCTCTCCGCACTGTAATGGATCACATACATTATGAGGATGAGAATACTCGTTACATTTGCAT
AGGACCTGTGAACAAGGTTTTGAATATGCTTTGCTGTTGGGTGGAAGATCCGAACTCAGAGGCTTTCAAGTTA
CATATACCAAGAATCTATGATTATCTGTGGATTGCTGAAGATGGCATGAAAATGCAGGGTTATAATGGAAGTC
AATTATGGGATACTGCTTTTGCTGTTCAAGCAATAATATCAACCAATCTTGTTGAAGAATATGGTGCAACTCTG
AAGAAAGCACATATGTACATAAAGAACTCACAGGTTTTAGAAGATTGCCCCGGTGATCTTGATTTTTGGTACCG
TCACATTTCAAAAGGTGCTTGGCCCTTCTCAACTGCAGATCACGGATGGCCTATCTCAGACTGTACAGCAGAGG
GTTTGAAAGCTGCTTTGTTATTATCAAAATTACCTTCGGAAGTTGTTGGTGAACCATTAGAGGCAAAACGGTTA
TATGATGCTGTGAATGTTATTCTTTCGTTACAGAATGCTGATGGTGGCTTTGCAACGTATGAGCTCACTAGATC
CTATCCTTGGTTGGAGTTCATCAATCCTGCTGAAACTTTTGGTGATATCGTGATTGATTATCCTTATGTGGAATG
TACCTCAGCTGCAATACAAGCTTTGACATCCTTTAAGAAATTATATCCTGGGCATCGACGGGAAGAAATAGATG
ATTGTATCAGAAAAGCTGTCATGTTTATCGAAAAGATTCAAGAATCAGATGGCTCATGGTATGGCTCATGGGG
TGTATGCTTCACTTATGGTGCATGGTTTGGGGTGAGAGGCTTAATGGCTGCTGGAATGAACTACAGTAATTGC
TCTAGCATCCGTAAAGCTTGTGATTTTCTGCTGTCAAAACAGCTTCCATCTGGTGGCTGGGGAGAGAGTTACCT
TTCATGTCAAAATAAGGTTTATTCAAATCTTGAAGGGGACAGGCCTCATTTAGTAAATACTGGATGGGCATTAT
TGGCTCTTATTGATGCTGGGCAGGCTGAGAGAGATCCAGCACCATTGCATCTTGCTGCAAGATATTTGATAGAT
TCACAAATGGATAATGGAGATTTCCCACAGCAGGAAATCATGGGAGTCTTCAATAAGAATTGTATGATCACTTA
CGCTGCATATAGAAACATTTTCCCTATTTGGGCACTAGGAGAGTACCGATGTCGGGTATTGCAGGTTTCCAAGT
AAACATTTTTATCATTTCAAATGCAATCGTCCACATGTTTTCTGATTTTTAAGCGTTTTTGTTTCCCTATTCATTTT
CAGCATTACTAGTAGTGACTTGTAGAAGTTTCCAACTGAAATTGAAAGAGATAAAACAGCTGTTTATATCAAAA
GAATAGTCCCGTGTATGTTTTGTTTTGCATGAATGAATTTGAAAAGTCCAGAACACAGGTTCAGGATCACCTTT
CTTAATGATGGCCGACAGCATTGATTGATTGAAATTTATATCTCCTTTTATGCTTATTGATTTGA

>TRINITY_DN88_c0_g1_i5 >TRINITY_DN88_c0_g1_i5 len=2373 path=[1:0-349 9:350-405 12:406-439 15:440-630 18:631-743 21:744-865 28:866-891 29:892-1028 31:1029-1052 33:1053-1082 34:1083-1107 36:1108-1293 37:1294-1369 39:1370-1848 40:1849-1884 42:1885-2372]

TCAATTTTTTTTTTTTTTAAAGGAAAAATTGCCTCTCTCTCTGTCTCTCGATTGAATCCTTAATCCTTCTAGATTCA
AATTTTGTCACTATCACGTAAAAGATTTAAAAAATGATATGGTGCAGATTTCGCCAGCTGCAGCTTTGCAAAGC
TACGACCATTCAGCCGCCAGCCAGCGTCACATATTGACCGGGCGATGCCACATGCGTGATCGTCGCTTACATTT
TCAATACTACCACTACCACTATTCTCTTTTTTTCACATCAAAACACATTGACAAGCTAAACATCCAGCAAAGCGA
CGTTTCACTCAAAATCAGTTCACTCTTTTTCTCCCTAATTTTCAGGTTTTAAGATGTGGAAGCTGAAGATCGCCG
AGGGAGGGAATGCGTGGCTGCGAACGACGAATAATCACGTCGGAAGACAAGTTTGGGAGTTCGACCCGAAG
CTCGGATCGCCGGAGGAGCTGGCGGAGATCGAAAAAGCTCGTCAAAATTTCTACAATAATCGCTTCGAGATGA
AGCACAGCGCCGATCTACTCATGCGCATTCAGTTTGCTAAGGAGAATCCAGGTGTTACAGTTATACCTCAAGTC
AAAGTAAAAGATACAGAAGATGTTACTGAGGAGATAGTGAAAACCACGTTAAGAAGGGCTATAAGTTACTAT
TCAACCATCCAGGCCGATGATGGGCACTGGCCTGGCGATTATGGAGGTCCTATGTTTCTCTTACCTGGTTTAGT
GATTGCTCTGTCTATCACTGGAGCACTGAATGCAGTTCTATCTGACGTACATCAAAAGGAGATGTGCCGTTATC
TCTACAATCACCAGAACAGAGACGGTGGGTGGGGTTTGCACATCGAGGGCCCAAGCACTATGTTTGGTACAGT
TTTGAGTTATATTACTTTAAGGTTGCTTGGTGAAGGAGCTAATGATGGGCAAGGAGCAATGGAGAGAGGGCG
TAACTGGATTCTAAGTCATGGTAGTGCAACTGCGATAACATCATGGGGAAAAATGTGGCTCTCAGTACTTGGA
GCTTTTGAATGGTCTGGCAATAATCCTCTTCCCCCTGAGATATGGCTTCTTACATATATGCTCCCATTCCATCCAG
GAAGGATGTGGTGTCACTGTCGCATGGTCTATTTGCCAATGTCTTACTTGTATGGAAAGAGGTTTGTTGGTCCA
ATCACCCCAACAGTATTGTCTTTGAGAAAGGAACTTTACAATGTCCCATATCATGAAATAGATTGGAGTGAAGC
ACGTAACCTATGTGCAAAGGAAGATTTGTACTACCCACACCCAATGATCCAGGATATACTTTGGGCAACACTTG
ACAAGGCCGTTGAACCTTTTCTATTGCACTGGCCTGGAAAGAAGTTGAGAGAAAAGGCTCTCCGCACTGTAAT
GGATCACATACATTATGAGGATGAGAATACTCGTTACATTTGCATAGGACCTGTGAACAAGGTTTTGAATATGC
TTTGCTGTTGGGTGGAAGATCCGAACTCAGAGGCTTTCAAGTTACATATACCAAGAATCTATGATTATCTGTGG
ATTGCTGAAGATGGCATGAAAATGCAGGGTTATAATGGAAGTCAATTATGGGATACTGCTTTTGCTGTTCAAG
CAATAATATCAACCAATCTTGTTGAAGAATATGGTGCAACTCTGAAGAAAGCACATATGTACATAAAGAACTCA
CAGGTTTTAGAAGATTGCCCCGGTGATCTTGATTTTTGGTACCGTCACATTTCAAAAGGTGCTTGGCCCTTCTCA
ACTGCAGATCACGGATGGCCTATCTCAGACTGTACAGCAGAGGGTTTGAAAGCTGCTTTGTTATTATCAAAATT
ACCATCAGAAGTTGTTGGCGAACCGTTAGAGGCAAAACAGTTATATGATGCTGTGAATGTTATTCTTTCGTTAC
AGAATGCTGATGGTGGCTTTGCAACGTATGAGCTCACTAGATCCTATCCTTGGTTGGAGTTCATCAATCCTGCT
GAAACTTTTGGTGATATCGTGATTGATTATCCTTATGTGGAATGTACCTCAGCTGCAATACAAGCTTTGACATCC
TTTAAGAAATTATATCCTGGGCATCGACGGGAAGAAATAGATGATTGTATCAGAAAAGCTGTCATGTTTATCG
AAAAGATTCAAGAATCAGATGGCTCATGGTATGGCTCATGGGGTGTATGCTTCACTTATGGTGCATGGTTTGG
GGTGAGAGGCTTAATGGCTGCTGGAATGAACTACAGTAATTGCTCTAGCATCCGTAAAGCTTGTGATTTTCTGC
TGTCAAAACAGCTTCCATCTGGTGGCTGGGGAGAGAGTTACCTTTCATGTCAAAATAAGGTTTATTCAAATCTT
GAAGGGGACA
